# Prenatal Urban Environment and Blood Pressure Trajectories From Childhood to Early Adulthood

**DOI:** 10.1016/j.jacadv.2023.100808

**Published:** 2024-01-08

**Authors:** Ana Gonçalves Soares, Susana Santos, Emie Seyve, Rozenn Nedelec, Soile Puhakka, Aino-Maija Eloranta, Santtu Mikkonen, Wen Lun Yuan, Deborah A. Lawlor, Jon Heron, Martine Vrijheid, Johanna Lepeule, Mark Nieuwenhuijsen, Serena Fossati, Vincent W.V. Jaddoe, Timo Lakka, Sylvain Sebert, Barbara Heude, Janine F. Felix, Ahmed Elhakeem, Nicholas J. Timpson

**Affiliations:** a MRC Integrative Epidemiology Unit, University of Bristol, Bristol, United Kingdom; b Population Health Sciences, Bristol Medical School, University of Bristol, Bristol, United Kingdom; c The Generation R Study Group, Erasmus Medical Center, University Medical Center Rotterdam, Rotterdam, The Netherlands; d Department of Pediatrics, Erasmus Medical Center, University Medical Center Rotterdam, Rotterdam, The Netherlands; e EPIUnit - Instituto de Saúde Pública, Universidade do Porto, Porto, Portugal; f Laboratório para a Investigação Integrativa e Translacional em Saúde Populacional (ITR), Universidade do Porto, Porto, Portugal; g Inserm, CNRS, Institute for Advanced Biosciences, Grenoble Alpes University, Grenoble, France; h Université Paris Cité and Université Sorbonne Paris Nord, Inserm, INRAE, Center for Research in Epidemiology and StatisticS (CRESS), Paris, France; i Faculty of Medicine, Research Unit of Population Health, University of Oulu, Oulu, Finland; j Department of Sports and Exercise Medicine, Oulu Deaconess Institute, Oulu, Finland; k Institute of Public Health and Clinical Nutrition, School of Medicine, University of Eastern Finland, Kuopio, Finland; l Institute of Biomedicine, School of Medicine, University of Eastern Finland, Kuopio, Finland; m Department of Medicine, Endocrinology and Clinical Nutrition, Kuopio University Hospital, Kuopio, Finland; n Department of Technical Physics, University of Eastern Finland, Kuopio, Finland; o Department of Environmental and Biological Sciences, University of Eastern Finland, Kuopio, Finland; p Singapore Institute for Clinical Sciences, Agency for Science, Technology, and Research (A∗STAR), Singapore, Singapore; q Barcelona Institute for Global Health (ISGlobal), Barcelona, Spain; r Universitat Pompeu Fabra (UPF), Barcelona, Spain; s CIBER Epidemiología y Salud Pública (CIBERESP), Madrid, Spain; t Department of Clinical Physiology and Nuclear Medicine, Kuopio University Hospital, Kuopio, Finland; u Foundation for Research in Health Exercise and Nutrition, Kuopio Research Institute of Exercise Medicine, Kuopio, Finland

**Keywords:** ALSPAC, blood pressure, cohorts, LongITools, trajectories, urban environment

## Abstract

**Background:**

Prenatal urban environmental exposures have been associated with blood pressure in children. The dynamic of these associations across childhood and later ages is unknown.

**Objectives:**

The purpose of this study was to assess associations of prenatal urban environmental exposures with blood pressure trajectories from childhood to early adulthood.

**Methods:**

Repeated measures of systolic blood pressure (SBP) and diastolic blood pressure (DBP) were collected in up to 7,454 participants from a UK birth cohort. Prenatal urban exposures (n = 43) covered measures of noise, air pollution, built environment, natural spaces, traffic, meteorology, and food environment. An exposome-wide association study approach was used. Linear spline mixed-effects models were used to model associations of each exposure with trajectories of blood pressure. Replication was sought in 4 independent European cohorts (up to 9,261).

**Results:**

In discovery analyses, higher humidity was associated with a faster increase (mean yearly change in SBP for an interquartile range increase in humidity: 0.29 mm Hg/y, 95% CI: 0.20-0.39) and higher temperature with a slower increase (mean yearly change in SBP per interquartile range increase in temperature: −0.17 mm Hg/y, 95% CI: −0.28 to −0.07) in SBP in childhood. Higher levels of humidity and air pollution were associated with faster increase in DBP in childhood and slower increase in adolescence. There was little evidence of an association of other exposures with change in SBP or DBP. Results for humidity and temperature, but not for air pollution, were replicated in other cohorts.

**Conclusions:**

Replicated findings suggest that higher prenatal humidity and temperature could modulate blood pressure changes across childhood.

Blood pressure is an important modifiable risk factor for cardiovascular disease (CVD)^1^^,^^2^ and it tracks from childhood into adulthood.^3^ Elevated blood pressure in childhood or adolescence is associated with several intermediate markers of CVD and with CVD morbidity and mortality in adulthood.^4^ There is a growing body of evidence showing that urban environmental exposures, such as air pollution,^5^ noise,^6^ temperature,^7^ and some characteristics of the built environment^8^ are associated with elevated blood pressure/hypertension in adulthood,^9^, ^10^, ^11^ and some associations have also been observed in children.^12^, ^13^, ^14^, ^15^

Early life, especially the prenatal and early postnatal periods, is a phase of rapid development and particularly vulnerable to environmental factors, during which adverse exposures may lead to higher risk of CVD.^16^^,^^17^ Some studies have shown associations of prenatal urban environmental exposures with blood pressure levels in children.^13^, ^14^, ^15^ Previous studies have mostly measured blood pressure at a single time point, and none have sought replication of the findings.

Studies exploring early-life urban environmental exposures and later blood pressure have predominantly focused on single exposures, or a specific, related group of exposures, particularly air pollution,^14^^,^^18^^,^^19^ and very few have used a more holistic view of the urban environment.^13^^,^^15^ To the best of our knowledge, no study has systematically explored a range of urban environmental exposures and their longitudinal association with blood pressure using repeated measures. Understanding whether associations vary over time is needed to determine the potential impact of the prenatal environment on future CVD.

Our aim was to assess the association of a range of prenatal urban environmental exposures with changes in systolic blood pressure (SBP) and diastolic blood pressure (DBP) from childhood to early adulthood.

## Methods

Data from the ALSPAC (Avon Longitudinal Study of Parents and Children) were used for the discovery analysis. ALSPAC is a prospective population-based cohort that recruited pregnant women living in the former Avon area of the United Kingdom who were due to give birth between April 1991 and December 1992.^20^, ^21^, ^22^ In total, 14,541 pregnancies were enrolled, which resulted in 14,062 live births. Children, mothers, and their partners have been followed up repeatedly ever since.^20^, ^21^, ^22^ The study website contains details of all the data that are available through a fully searchable data dictionary and variable search tool.

Ethical approval for the study was obtained from the ALSPAC Ethics and Law Committee and the Local Research Ethics Committees. Informed consent for the use of data collected via questionnaires and clinics was obtained from participants following the recommendations of the ALSPAC Ethics and Law Committee at the time. Consent for biological samples has been collected in accordance with the Human Tissue Act (2004).

### Urban environmental exposures

In total, 43 urban environmental exposures were included in this study (Supplemental Table 1). All exposures were derived as part of the LifeCycle Project^23^^,^^24^ and cover noise, air pollution, built environment, natural spaces, traffic, meteorology, and unhealthy food environment. The only exception was particulate matter <10 μm (PM_10_), which in ALSPAC was not modeled as part of LifeCycle.^25^

Briefly, exposures were assigned within geographic information system tools to the geocoded addresses of the participants at birth. Exposures correspond to average exposure levels during pregnancy. Continuous exposures were rescaled for analysis to represent changes by interquartile range (IQR) increase. Further details on the exposures can be found in Supplemental Appendix.

Pairwise Pearson correlation coefficients across all exposures were estimated; for any r >0.9, only one of the pair was included in subsequent analyses, based on variable properties (eg, continuous preferred to categorical variables, mean preferred to minimum and maximum).

### Blood pressure

Blood pressure was measured in all ALSPAC participants at clinic assessments at average ages 7, 9, 10, 11, 13, 15, 18, and 24 years, and at ages 3, 4, and 5 years in a 10% subsample.^20^ In all clinic assessments, SBP and DBP were measured twice with the individual sitting at rest with the arm supported, using a cuff size appropriate for the child’s upper arm circumference. The mean of the 2 measures was recorded and used in the analyses. More details on blood pressure measurement are described in the Supplemental Appendix.

### Confounders

Analyses were adjusted for a set of confounders defined as known or plausible determinants of urban environment and blood pressure (ie, maternal education, age at delivery, ethnicity, and area deprivation). Sex, although not a confounder, was also included in the adjustment to reduce residual variance. We did not adjust for birthweight or height, as these are possible mediators of the association between prenatal environmental exposures and blood pressure trajectories.

All confounders were assessed during pregnancy and obtained through questionnaires, except for area deprivation, which was based on data from the UK Ministry of Housing, Communities & Local Government and linked to the geocoded addresses at birth. More details on the confounders are presented in the Supplemental Appendix.

### Statistical analysis

We included participants who had information on at least one prenatal urban environmental exposure and at least one blood pressure measurement between mean ages 3 and 24 years, and who had complete data on the confounders. This resulted in the inclusion of 5,095 (for noise exposures) to 7,454 (for average greenness exposures) participants with between 28,132 and 41,216 blood pressure observations (Supplemental Figure 1).

An exposome-wide association study (ExWAS) approach^26^ was used to assess the association of each exposure individually with SBP and DBP trajectories from childhood to early adulthood. Associations were examined using linear mixed-effects models with linear splines for age (as a fixed effect) to allow for nonlinear change in blood pressure with age.^27^ Knots were placed at approximate ages 10 and 18 years to summarize linear change in blood pressure across childhood (3-10 years), adolescence (10-18 years), and young adulthood (18-26 years) (more details in the Supplemental Appendix). Models included random intercept and random linear slope for age to allow for between-individual differences in blood pressure at baseline and in change with age. Age was centered at 3.1 (mean age at first clinic assessment). An interaction term between each exposure and age was included in the models to assess the association of each urban environmental exposure with change in blood pressure. All models were adjusted for the confounders defined above.

To limit false positive results, a Bonferroni correction was used to account for multiple testing; an alpha level of 5% was divided by the number of exposures assessed (39 after removing one of highly correlated pairs, ie, 0.05/39 = *P* threshold 0.0013). For associations that reached our multiple-testing *P* value threshold, average predicted means for SBP and DBP from childhood to early adulthood in the 25th and 75th percentile of the exposure were calculated from the linear mixed-effects models.

Within each exposure-blood pressure trajectory, complete case analyses were undertaken, which gives unbiased results when the chance of being a complete case is independent of the outcome after taking the covariates into consideration.^28^ To explore the potential impact of missing data, we compared characteristics of those included and not included in the analysis due to missing data on one or more confounders. The analyses were performed in the software Stata 17.0 (Statacorp, College Station, TX, USA) and the software MLwiN 3.05 was accessed from within Stata using *runmlwin*.^29^ Data visualization was also performed in R 4.3.0 using the package *ggplot2*.

### Sensitivity analyses

To explore the sensitivity of our results to including all participants with at least one blood pressure measurement, we also repeated analyses with only participants who had at least 3 repeated measures (up to 6,128 with 39,358 observations).

We explored possible sex differences in the associations between prenatal urban environmental exposures and blood pressure trajectories in those exposures found to be associated with blood pressure in the main analysis after multiple-testing correction. For that, we included in the models a 3-way interaction term between exposure, age and sex, along with an interaction between age and sex, and assessed the significance of the 3-way interaction (*P* < 0.05). We also performed sex-stratified analyses.

### Replication analysis

To assess robustness in the results found in ALSPAC, we sought replication, for associations that reached our multiple-testing *P* value threshold, in 4 independent European cohorts participating in the LongITools Project.^30^ These were EDEN (Etude des Déterminants pré et post natals précoces du développement psychomoteur et de la santé de l’ENfant),^31^ the GenR (Generation R Study),^32^ the PANIC (Physical Activity and Nutrition in Children Study),^33^ and the NFBC1986 (Northern Finland Birth Cohort 1986).^34^ Identical methods to those used in ALSPAC for deriving exposures were used in EDEN and GenR, with the methods used in NFBC1986 and PANIC described in the Supplemental Appendix. Blood pressure measurements were available at average ages (years) 2.1, 6.1, and 9.8 in GenR, 3.1 and 5.6 in EDEN, 7.6, 9.8, and 15.8 in PANIC, and 16.0 and 34.1 in NFBC1986 (Supplemental Figure 2). Information on blood pressure measurement in each study is available in the Supplemental Appendix.

Linear mixed-effects models with random intercept and a random linear slope for age were used to model blood pressure trajectories in each study. Limited numbers of repeated measurements meant we had to use linear mixed-effects models without splines. In each study, age was centered at the mean age at baseline (which differed in each cohort). The set of available confounders may vary across studies; hence, the adjustment model may differ from the initial analysis in ALSPAC (more details in the Supplemental Appendix). Results were considered to be replicated if the direction of association was consistent with the ALSPAC results and *P* < 0.05.

## Results

In ALSPAC, up to 7,454 individuals with 41,216 blood pressure observations were included in the analysis. Those included were more likely to have mothers with higher education and older age at delivery, to have White ethnicity and to live in a less deprived area at birth than those not included in the analysis due to missing data on one or more of the confounders (Supplemental Table 2). Up to 11 measures of blood pressure were used (mean 6.7 ± 2.1), the mean age of the participants ranged from 3.1 ± 0.02 at baseline to 24.4 ± 0.79 years at the last follow-up, and the number of individuals with blood pressure measure in each assessment varied from 800 (at age 5 years) to 6,056 (at age 7.5 years) (Supplemental Table 3).

Out of the 43 environmental exposures, 4 were excluded due to very high correlation (Supplemental Figure 3). The distribution of the environmental exposures (in their original scale) is presented in Supplemental Table 4.

The average trajectories of SBP and DBP from childhood to early adulthood are presented in Supplemental Figure 4 and Supplemental Table 5. The mean SBP at baseline (mean age 3.1 y) was 94.3 mm Hg (95% CI: 89.9-92.7 mm Hg), and it increased on average 1.56 mm Hg/y (95% CI: 1.50-1.62 mm Hg/y) in childhood (3-10 years) and 2.57 mm Hg/y (95% CI: 2.53-2.61 mm Hg/y) in adolescence (age 10-18 years), and decreased on average 1.02 mm Hg/y (95% CI: −1.08 to −0.97 mm Hg/y) in early adulthood (age 18-26 years). The mean DBP at baseline was 54.9 mm Hg (95% CI: 53.9-55.9 mm Hg), and it increased on average 0.17 mm Hg/y (95% CI: 0.12-0.21 mm Hg/y) in childhood, 1.06 mm Hg/y (95% CI: 1.03-1.09 mm Hg/y) in adolescence, and 0.14 mm Hg/y (95% CI: 0.09-0.18 mm Hg/y) in early adulthood.

### Associations of environmental exposures with blood pressure trajectories

**Figure 1:**
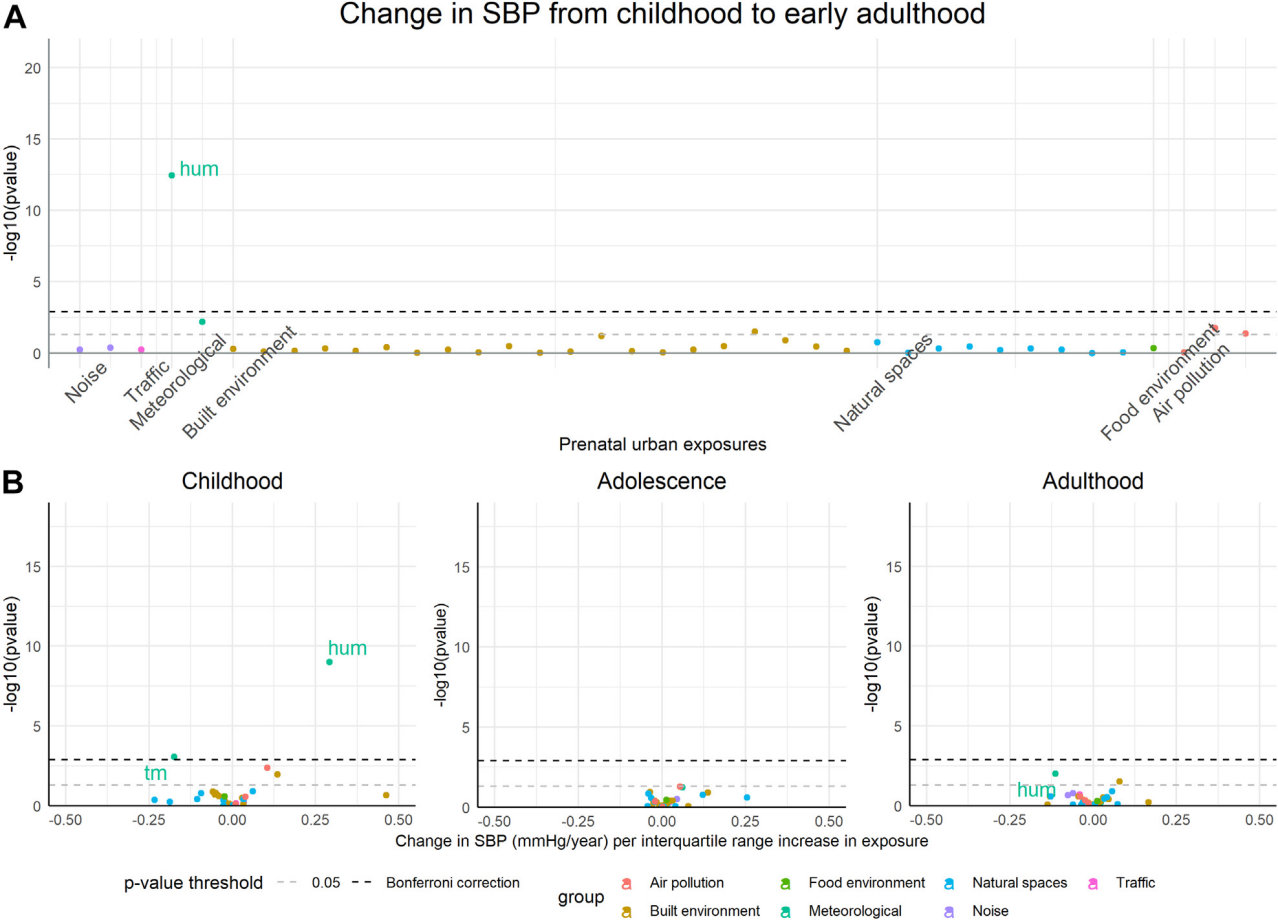
Associations of Urban Environmental Exposures With Changes in SBP in ALSPAC (A) Associations of prenatal urban environmental exposures with SBP from childhood to early adulthood, and (B) volcano plots showing the associations of urban exposures with changes in SBP in childhood, adolescence and early adulthood. Beta corresponds to mean changes in SBP per interquartile range (IQR) increase/change in category in the exposure; the IQR for each exposure is presented in Supplemental Table 4. Models are adjusted for maternal education, age at delivery, ethnicity, area deprivation, and sex. ALSPAC = Avon Longitudinal Study of Parents and Children; SBP = systolic blood pressure.

**Figure 2:**
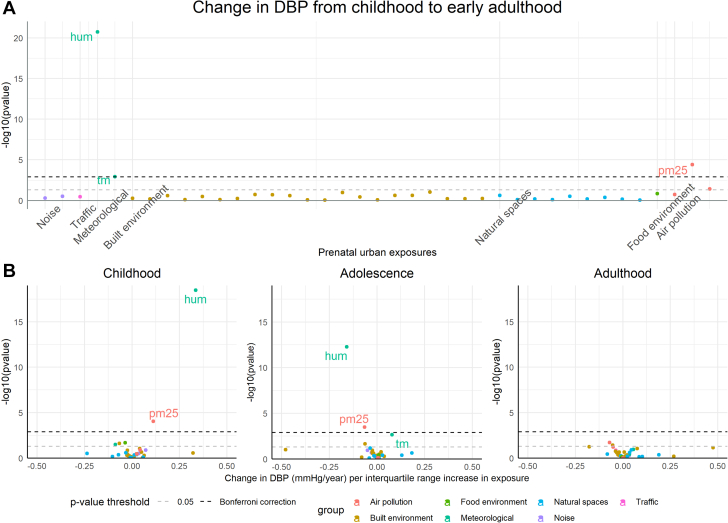
Associations of Urban Environmental Exposures With Changes in DBP in ALSPAC (A) Associations of prenatal urban environmental exposures with DBP from childhood to early adulthood, and (B) volcano plots showing the associations of urban exposures with changes in DBP in childhood, adolescence and early adulthood. Beta corresponds to mean changes in DBP per IQR increase/change in category in the exposure; the IQR for each exposure is presented in Supplemental Table 4. Models are adjusted for maternal education, age at delivery, ethnicity, area deprivation, and sex. ALSPAC = Avon Longitudinal Study of Parents and Children; DBP = diastolic blood pressure.

The associations of urban environmental exposures (n = 39) with change in SBP and DBP from childhood to early adulthood are presented in Figures 1 and 2 and Supplemental Tables 6 and 7.

After Bonferroni correction, prenatal humidity and temperature were associated with change in SBP from childhood to early adulthood (Figure 1A). Higher relative humidity was associated with a faster increase in SBP in childhood (mean change in SBP for an IQR increase in humidity (which corresponds to 2% increase in humidity [Supplemental Table 4]): 0.29 mm Hg/y, 95% CI: 0.20-0.39 mm Hg/y), and slower decrease in SBP in adulthood (mean change in SBP for an IQR increase in humidity: −0.11 mm Hg/y, 95% CI: −0.20 to −0.03 mm Hg/y) (Figure 1B, Supplemental Table 6). Higher mean temperature was associated with a slower increase in SBP in childhood (mean change in SBP for an IQR increase in temperature: −0.18 mm Hg/y, 95% CI: −0.28 to −0.08 mm Hg/y). There was little evidence for an association between the other urban environmental exposures and change in SBP from childhood to early adulthood.

**Figure 3:**
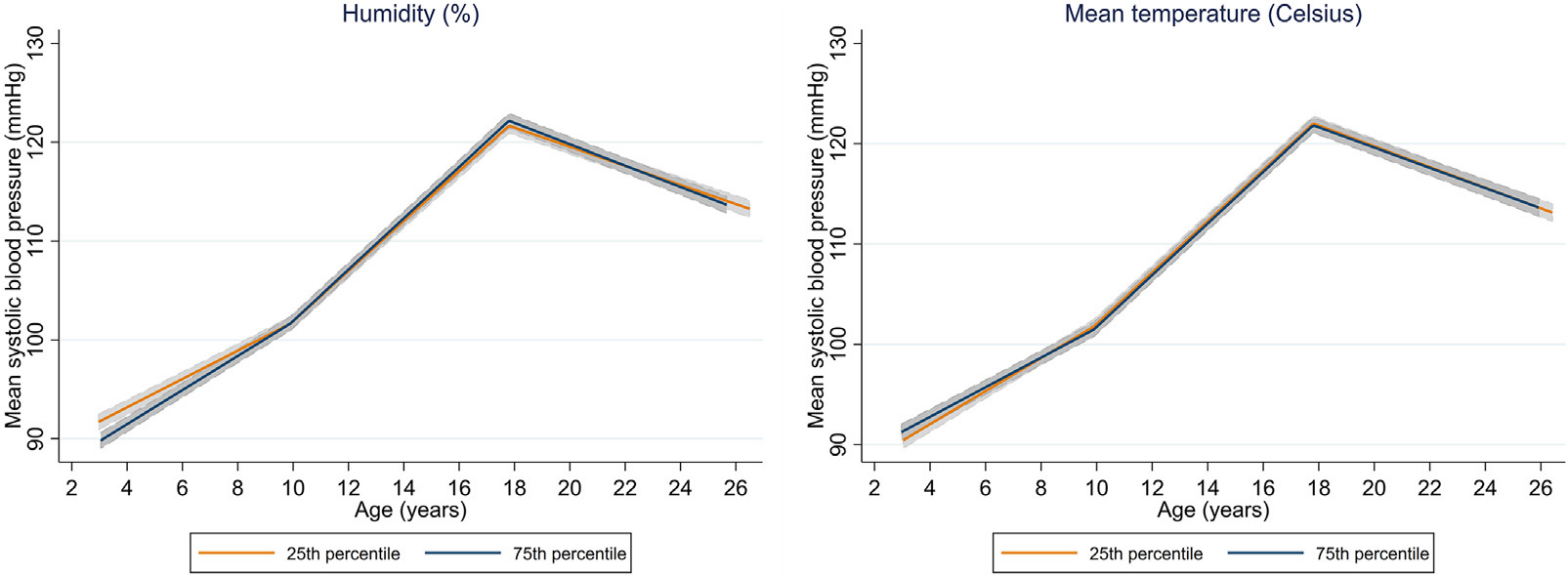
Average Predicted Population Mean for Systolic Blood Pressure From Childhood to Early Adulthood in the 25th and 75th Percentiles of Relative Humidity and Mean Temperature

The average predicted trajectory of SBP from childhood to early adulthood in the 25th and 75th percentiles of humidity and mean temperature is presented in Figure 3 and Supplemental Table 8. For example, the average SBP in those in the 25th percentile of humidity (average relative humidity 81.1%) increased from 91.9 mm Hg (95% CI: 91.1-92.7 mm Hg) at baseline to 101.8 mm Hg (95% CI: 101.1-102.5 mm Hg) at age 10, while in the 75th percentile of humidity (relative humidity 83.1%) the increase was faster, from 89.9 mm Hg 95% CI: 89.1-90.7 mm Hg) to 101.8 mm Hg (95% CI: 101.1-102.5 mm Hg) in the same period.

**Figure 4:**
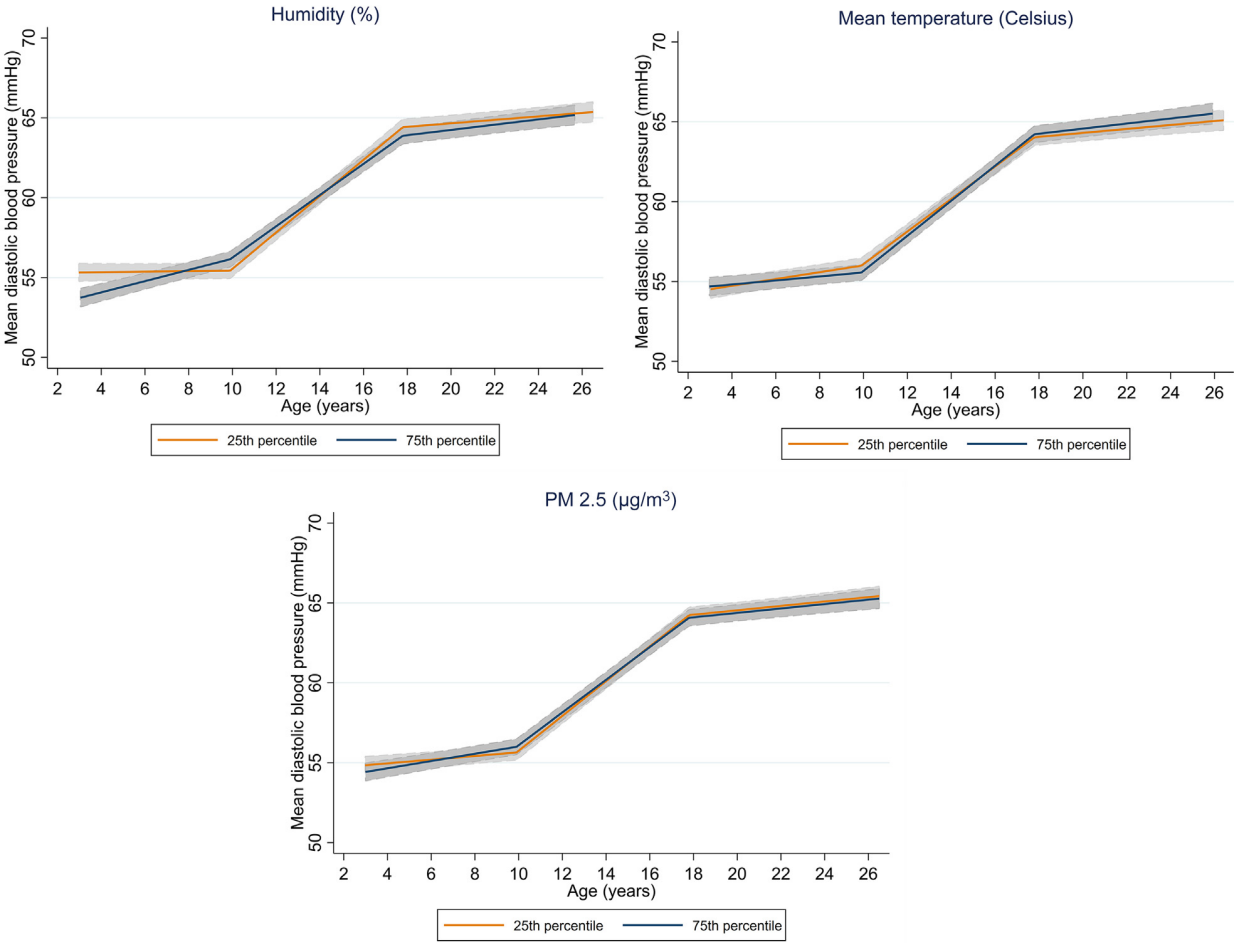
Average Predicted Population Mean for Diastolic Blood Pressure From Childhood to Early Adulthood in the 25th and 75th Percentiles of Relative Humidity, Mean Temperature, and PM_2.5_

Prenatal humidity, temperature, and PM_2.5_ were associated with change in DBP from childhood to early adulthood, after multiple-testing correction (Figure 2A). Higher humidity was associated with faster increase in DBP in childhood and slower increase in adolescence (mean change in DBP for an IQR increase in humidity: 0.34 mm Hg/y, 95% CI: 0.26, 0.41, and −0.16 mm Hg/y, 95% CI: −0.20 to −0.11 mm Hg/y, respectively) (Figure 2B, Supplemental Table 7). Associations in the opposite direction were observed for temperature (mean change in DBP for an IQR increase in temperature: −0.09 mm Hg/y in childhood, 95% CI: −0.17 to −0.01 mm Hg/y, and 0.08 mm Hg/y in adolescence, 95% CI: 0.03-0.13 mm Hg/y). Higher PM_2.5_ was associated with a faster increase in DBP in childhood and a slower increase in adolescence (mean change in DBP for an IQR increase in PM_2.5_: 0.11 mm Hg/y, 95% CI: 0.06-0.17 mm Hg/y, and −0.07 mm Hg/y, 95% CI: −0.10 to −0.03 mm Hg/y, respectively). Little evidence for an association between the other urban environmental exposures and change in DBP from childhood to early adulthood was observed. The average predicted trajectory of DBP from childhood to young adulthood in the 25th and 75th percentiles of humidity, temperature, and PM_2.5_ is presented in Figure 4 and Supplemental Table 8.

Results for the associations of the urban environmental exposures with SBP and DBP trajectories remained similar when analyses were restricted to individuals with 3 or more repeated measures of blood pressure (Supplemental Tables 9 and 10).

Overall associations of humidity, mean temperature, and PM_2.5_ with blood pressure trajectories were similar in males and females, and there was little evidence for sex differences (Supplemental Table 11).

### Replication in other cohorts

The distribution of the baseline characteristics, environmental exposures, and outcomes at each follow-up in GenR, EDEN, PANIC, and NFBC1986 are presented in Supplemental Table 12. The association of the confounders with the environmental exposures in all the cohorts is presented in Supplemental Table 13. While some associations between the confounders and the environmental exposures were similar across the cohorts (eg, those living in the most deprived areas had higher levels of PM_2.5_), some associations differed (eg, low maternal education was positively associated with PM_2.5_ in ALSPAC and EDEN (Poitiers), while no association was evident in EDEN (Nancy) and an inverse association was observed in GenR).

**Figure 5:**
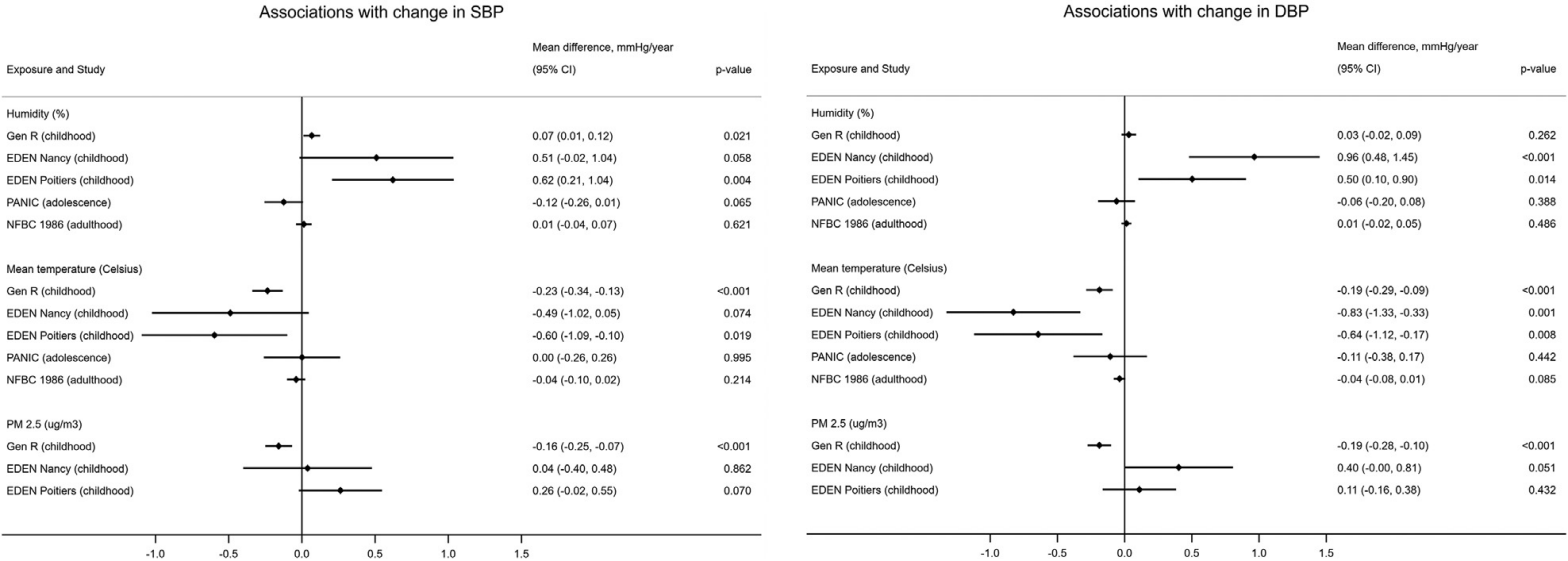
Associations of Humidity, Mean Temperature, and PM2.5 With Change in SBP and DBP in GenR, EDEN, PANIC, and NFBC1986 DBP = diastolic blood pressure; EDEN = Etude des Déterminants pré et post natals précoces du développement psychomoteur et de la santé de l'Enfant; GenR = Generation R Study; NFBC1986 = Northern Finland Birth Cohort 1986; PANIC = Physical Activity and Nutrition in Children Study; SBP = systolic blood pressure.

The associations of humidity, mean temperature, and PM_2.5_ with SBP and DBP at baseline in each study are presented in Supplemental Figure 5, and associations with change in SBP and DBP are presented in Figure 5. The association of humidity with faster increase in SBP in childhood replicated in GenR and EDEN (Poitiers), and with faster increase in DBP replicated in EDEN. An association between humidity and change in SBP in adulthood was not observed in NFBC1986. The associations of temperature with slower increase in SBP and DBP in childhood were also observed in GenR and EDEN, but the association of temperature with faster increase in DBP in adolescence did not replicate in PANIC. The association between PM_2.5_ and faster increase in DBP in childhood was also observed in EDEN (Nancy), though CI spanned the null, and in GenR associations between PM_2.5_ and slower increase in both SBP and DBP in childhood were observed. No data were available for PM_2.5_ in adolescence.

## Discussion

We assessed the association of 39 prenatal urban environmental exposures with blood pressure trajectories from childhood to early adulthood in ALSPAC (Central Illustration) and sought replication of the findings in 4 independent European cohorts. After accounting for multiple testing, we found that humidity was associated with faster increase in SBP and DBP in childhood, slower decrease in SBP in adulthood, and slower increase in DBP in adolescence. Temperature was associated with slower increase in SBP and DBP in childhood and faster increase in DBP in adolescence. PM_2.5_ was associated with faster increase in DBP in childhood and slower increase in adolescence. Little evidence for sex differences in these associations was observed. Analyses in independent cohorts replicated results for associations of humidity and temperature with change in blood pressure in childhood but not for PM_2.5_.

**Central Illustration:**
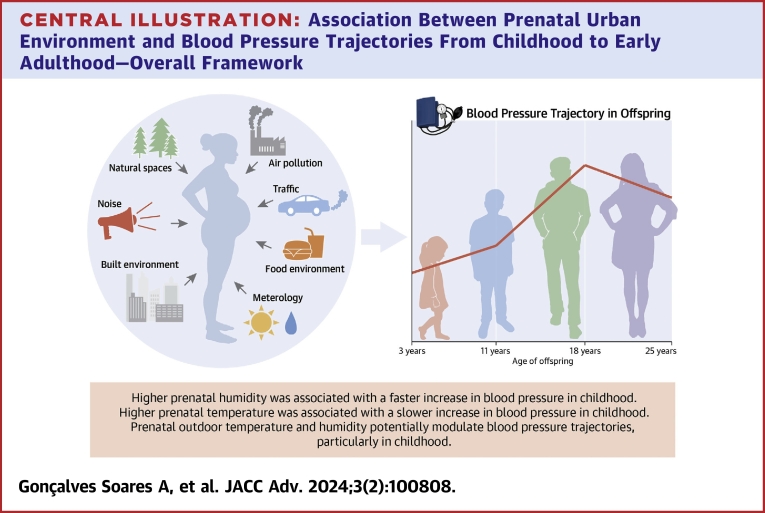
Association Between Prenatal Urban Environment and Blood Pressure Trajectories From Childhood to Early Adulthood: Overall Framework

Many studies have assessed the association of outdoor temperature with blood pressure,^7^ but less evidence is available for humidity. In adults, humidity has been positively associated with blood pressure levels,^35^ and a positive association with DBP has also been observed in children.^15^ We were able to identify only one study which assessed the association of humidity during pregnancy with offspring blood pressure. This study, which included 1,277 European children did not find an association between prenatal humidity and blood pressure in children aged 6 to 11 years (mean difference per IQR increase in humidity: −0.76 mm Hg, 95% CI: −3.3 to 1.77 mm Hg for SBP, and −0.44 mm Hg, 95% CI: −2.79 to 1.92 mm Hg for DBP).^13^

Findings from the same study cited above showed that outdoor temperature during pregnancy was associated with higher SBP and DBP, though CI for the latter spanned the null (mean difference per IQR increase in temperature: 1.6 mm Hg, 95% CI: 0.2-2.9 mm Hg, and 1.2 mm Hg, 95% CI: −0.1 to 2.4 mm Hg, respectively).^13^ In 4,279 European children aged 4 to 5 years, the association of prenatal temperature with SBP and DBP was more imprecise and CIs included zero (mean difference per IQR increase in temperature: 1.16 mm Hg, 95% CI: −0.09 to 2.41 mm Hg, and 0.45 mm Hg, 95% CI: −0.68 to 1.58 mm Hg, respectively).^15^ Our results corroborate these findings and extend research in those studies by showing that higher prenatal temperature was associated with slower increase in SBP and DBP in childhood. This slower change in blood pressure during childhood could suggest that these associations might no longer be evident in the long-term, or that inverse associations between prenatal temperature and blood pressure might be observed in older ages. A study in China with 6,158 individuals (mean age 40 ± 9.0 years) showed an inverse association between temperature during pregnancy and blood pressure in adulthood.^36^ Those born between April and August (and therefore with longer exposure to cold spells during pregnancy) had higher DBP (and also SBP in males only) than those born between September and March.^36^

Outdoor temperature and relative humidity are often correlated (r = −0.50 in our study) and vary across seasons, and associations with blood pressure were similar when these exposures were mutually adjusted and further adjusted for month of birth (Supplemental Table 14). The mechanisms underlying the association between outdoor temperature and blood pressure are not fully understood, but it is suggested that lower ambient temperature activates the sympathetic nervous system and increases vasoconstriction leading to increase in heart rate and blood pressure.^7^^,^^35^ However, the mechanisms through which outdoor temperature (and humidity) during pregnancy might influence offspring blood pressure require further investigation.

Although PM_2.5_ was associated with faster increase in DBP in childhood, this finding did not replicate in the independent cohorts. In GenR, an inverse association between PM_2.5_ and blood pressure changes was found. It is possible that these contrasting results are driven by the different levels of exposure and different confounding structure across the cohorts; for example, air pollution levels are higher in GenR than in the other studies, and the association between socioeconomic factors and air pollution differed across cohorts. Previous studies have shown that prenatal exposure to PM_2.5_ and PM_10_ was associated with higher blood pressure in newborns,^19^^,^^37^ particularly exposure in the third trimester of pregnancy.^19^ Prenatal NO_2_ was associated with higher DBP in children aged 4 to 5 years (mean difference per IQR increase in NO_2_: 0.85 mm Hg, 95% CI: 0.38-1.33 mm Hg), but less evidence was observed for PM_2.5_ and PM_10_ (mean difference per IQR increase in PM_2.5_ and PM_10_: 0.45, 95% CI: −0.11 to 1.02 mm Hg, and −0.08, 95% CI: −0.76 to 0.61 mm Hg, respectively).^15^ There are numerous postulated biological mechanisms linking prenatal exposure to air pollution to children’s adverse cardiometabolic health, including direct placental translocation of ultrafine particles, placental and systemic maternal oxidative stress and inflammation elicited by both fine and ultrafine particulate matter, epigenetic changes, and potential endocrine effects.^38^

In our study, most associations with change in blood pressure were observed in childhood. It is possible that any effects of prenatal environmental exposures on blood pressure trajectory are no longer evident at later ages. However, it is important to highlight that contemporaneous exposure to urban environmental factors can affect blood pressure levels,^5^^,^^6^^,^^9^^,^^10^^,^^12^^,^^38^ and the long-term exposure to adverse environments might affect blood pressure trajectories across the life course.

Very few studies have explored whether the association between prenatal urban environment and child’s health differs by sex. A study with 822 American children aged 4 to 6 years showed that associations between prenatal exposure to air pollution and child’s DBP were stronger in females.^18^ In adults, there is suggestion that the association between air pollution and blood pressure is stronger in males.^5^ In our study, we found little evidence of sex differences in the associations between prenatal environmental exposures and blood pressure trajectories.

### Strengths and limitations

We explored the longitudinal association between urban environment in pregnancy and offspring blood pressure using repeated data spanning from childhood to early adulthood. We used an ExWAS approach to assess the association of a range of urban environmental exposures with blood pressure trajectories, taking account of multiple testing and attempting to replicate findings in independent cohorts were possible, in order to minimize chance findings. A limitation of this approach is that it did not consider between-exposure confounding, potential interactions or nonlinear associations, which are methodologically challenging. Our analyses included all participants with at least one blood pressure measure, under the missing at random assumption (ie, the probability that an outcome value is missing depends on observed values of the outcome, conditional on the covariates in the model), which may have reduced selection bias due to missing outcome data. Violation of this assumption could bias our results, but when we repeated analyses only including those with at least 3 measures we found similar results. We restricted the analyses to those with complete data on confounders, which gives unbiased estimates if having complete data is independent of the outcome, after taking the covariates into consideration.^28^ It is not possible to test this assumption in our data, but having complete data is likely to depend on SBP (Supplemental Table 15). While analyses for DBP are likely to be unbiased, analyses for SBP might be overestimated, since those included in the analysis had a higher increase in SBP in childhood. The exposures were assigned at geocoded addresses at birth, and it is possible that this differs from the address during pregnancy. However, in ALSPAC about 90% of the participants were living in the same address during pregnancy than the one at birth. Misclassification is expected to be differential across exposures and different sample sizes might result in different statistical power. The age range of the cohorts included in the replication differed, and although we attempted replication of the results found in ALSPAC, power was limited in some age ranges. The lack of some of our predefined confounders in some of the replication cohorts might have influenced the replication results.

## Conclusions

Using an ExWAS approach to systematically assess a range of prenatal urban environmental exposures, this study showed that prenatal outdoor temperature and humidity potentially modulate blood pressure trajectories, particularly in childhood. Our study contributes to the growing body of evidence on the longitudinal associations of prenatal environmental exposures with blood pressure later in life.

PERSPECTIVES **COMPETENCY IN MEDICAL KNOWLEDGE:** Prenatal outdoor temperature and relative humidity can potentially modulate blood pressure trajectories, especially in childhood.

**TRANSLATIONAL OUTLOOK:** More understanding on how meteorological conditions during pregnancy might affect offspring blood pressure is needed to inform strategies to prevent cardiovascular disease in adulthood related to prenatal urban exposures.

## Funding support and author disclosures

The data that support the findings of this study are available from the authors upon reasonable request and approval from the respective cohorts executive committees. Restrictions apply to the availability of these data, which were used under license for the current study, and are not publicly available. Requests for access to ALSPAC data may be sent to the ALSPAC Executive Committee at https://proposals.epi.bristol.ac.uk/. Requests for access to the Generation R Study data can be addressed to the Director of the Core Facility Generation R (generationr@erasmusmc.nl) and will be evaluated by the Generation R Management Team taking into account local rules and regulations. Researchers interested in exploring EDEN data should contact directly barbara.heude@inserm.fr to complete a dedicated project form for evaluation by the EDEN steering committee. Requests to access NFBC data may be sent to the NFBC project centre (NFBCprojectcenter@oulu.fi). Requests for access to the PANIC study data can be addressed to timo.lakka@uef.fi. This project received funding from the European Union’s 10.13039/100010661Horizon 2020 research and innovation programme (874739 LongITools; 733206 LifeCycle; 874583 ATHLETE; 824989 EUCAN-Connect; 101021566 ART-HEALTH). Drs Gonclaves Soares, Elhakeem, Lawlor, Heron, and Timpson work in a Unit that is funded by the 10.13039/501100000265UK Medical Research Council (MC_UU_00011/1&6) and the 10.13039/501100000883University of Bristol. Dr Lawlor is a National Institute of Health Research Senior Investigator (NF-0616-10102) and is also supported by a 10.13039/501100000274British Heart Foundation Chair (CH/F/20/90003). The 10.13039/501100000265UK Medical Research Council and 10.13039/100004440Wellcome (Grant ref: 217065/Z/19/Z), and the 10.13039/501100000883University of Bristol provide core support for ALSPAC. A comprehensive list of grants funding is available on the ALSPAC website (http://www.bristol.ac.uk/alspac/external/documents/grant-acknowledgments.pdf). The general design of the Generation R Study is made possible by financial support from 10.13039/501100003061Erasmus MC, University Medical Center Rotterdam, 10.13039/501100022550Erasmus University Rotterdam, the Netherlands Organization for Health Research and Development and the Ministry of Health, Welfare and Sport. Dr Mikkonen acknowledges the Academy of Finland competitive funding to strengthen university research profiles (PROFI) for the 10.13039/100007753University of Eastern Finland (grant no. 325022). The funders had no role in the design and conduct of the study; collection, management, analysis, and interpretation of the data; preparation, review, or approval of the manuscript; and decision to submit the manuscript for publication. Dr Lawlor has received grants from national and international government and charity funders, Roche Diagnostics, and Medtronic Ltd for work unrelated to this publication. All other authors have reported that they have no relationships relevant to the contents of this paper to disclose.
