# Supplementary material for: Prenatal Urban Environment and Blood Pressure Trajectories From Childhood to Early Adulthood
Source: JACC Adv. 2024 Jan 8;3(2):100808. doi: 10.1016/j.jacadv.2023.100808 (PMC11198279; doi:10.1016/j.jacadv.2023.100808)
Supplement: Supplemental methods, Figures 1-5 and Tables 1-15 [file mmc1.pdf]

Prenatal urban environment and blood pressure trajectories from childhood to early adulthood

Supplemental material

Table of contents

Supplemental methods 3

Supplemental results 10

Supplemental Table 1. Description of the urban environmental exposures assessed in the Avon Longitudinal Study of Parents and Children (ALSPAC) 10

Supplemental Table 2. Baseline characteristics of the ALSPAC participants included (n=7,454) and not included in the analysis (n=1,780) due to missing data on one or more of the confounders 13

Supplemental Table 3. Distribution of age, systolic and diastolic blood pressure in each assessment in the ALSPAC participants included in the analysis (n=7,454) 14

Supplemental Table 4. Distribution of the urban environmental exposures in ALSPAC 15

Supplemental Table 5. Adjusted mean trajectories of systolic and diastolic blood pressure from childhood to early adulthood in ALSPAC 18

Supplemental Table 6. Associations between urban environmental exposures and changes in systolic blood pressure (SBP) in ALSPAC 19

Supplemental Table 7. Associations between urban environmental exposures and changes in diastolic blood pressure (DBP) in ALSPAC 26

Supplemental Table 8. Average predicted systolic and diastolic blood pressure according to different levels of urban environmental exposures in ALSPAC 33

Supplemental Table 9. Associations between urban environmental exposures and changes in systolic blood pressure (SBP) in ALSPAC, restricted to those with 3 or more blood pressure measurements. 34

Supplemental Table 10. Associations between urban environmental exposures and changes in diastolic blood pressure (DBP) in ALSPAC, restricted to those with 3 or more blood pressure measurements 41

Supplemental Table 11. Associations of urban environmental exposures with systolic (SBP) and diastolic blood pressure (DBP) in males and females in ALSPAC. 48

Supplemental Table 12. Distribution of baseline characteristics, environmental exposures and outcomes in the participants included in the analysis in GenR, EDEN, PANIC and NFBC1986 50

Supplemental Table 13. Association between confounders and urban environmental exposures in each cohort 52

Supplemental Table 14. Associations of humidity and temperature, mutually adjusted, with changes in systolic (SBP) and diastolic blood pressure (DBP) in ALSPAC 54

Supplemental Table 15. Association between being a complete case and trajectory of systolic and diastolic blood pressure from childhood to early adulthood in ALSPAC (n=9,234)............................55
Supplemental Figure 1. Flow of participants in ALSPAC 56
Supplemental Figure 2. Average age at the time of blood pressure measurements available in each study...57
Supplemental Figure 3. Correlation matrix of the urban environmental exposures in ALSPAC..........58
Supplemental Figure 4. Observed and average predicted (A) systolic blood pressure and (B) diastolic blood pressure trajectories in the ALSPAC. 59
Supplemental Figure 5. Associations of humidity, mean temperature, and $PM_{2.5}$ with SBP and DBP at baseline in GenR, EDEN, PANIC and NFBC1986. 60

Supplemental methods

Environmental exposures

The environmental exposures were derived as part of the LifeCycle Project. The protocol for the exposures in the urban environment can be found in <https://lifecycle-project.eu/wp-content/uploads/2021/07/Protocol_v4_2021_06_25.pdf>.

A Geographic Information System (GIS) environment for all study areas within the LifeCycle Project was set up at ISGLOBAL, in Barcelona, for centralised processing of the data, in collaboration with each of the cohorts involved. Details on geocoding and linkage to third party physical environment records in the Avon Longitudinal Study of Parents and Children (ALSPAC) have been previously published.[1] The residential geocodes of the participants address history were transferred to a central database held in ISGLOBAL.

<u>Air pollution</u>

For air pollution, exposure estimates for nitrogen dioxide (NO_2) and particulate matter < 2.5 µm ($PM_{2.5}$) were based on the land use regression (LUR) modelling approach developed in the ELAPSE Project.[2] Temporal adjustment was conducted using background routine monitoring stations. Temporally adjusted exposure levels to each pollutant were estimated by combining the LUR spatial estimates of pollutants for their geocode with a temporal adjusting factor obtained from the routine monitoring data. Specifically, the ratio of the concentration of the routine monitor of each day of the study period and the annual average during 2010 as the adjustment factor for that day were used. When data on a specific pollutant were not available from the routine network we did a back-extrapolation based on NO_2. Data on NO_2 was obtained from routine background stations.

For particulate matter < 10 µm (PM_{10}), a detailed description is available elsewhere.[3] ADMS-Urban model was used to estimate local traffic and non-traffic daily PM_{10} within the study area. The NAME-III air pollution model (Numerical Atmospheric-dispersion Modelling Environment) was used to estimate regional/long-range sources (i.e. outside the study area) of daily PM_{10} using meteorological data taken from the ERA-Interim meteorological reanalysis produced by European Centre for Medium-Range Weather Forecasts (ECMWF) and pollution data in the form of daily average concentrations of PM_{10} for 14 receptor locations. A constant was included in the model to account for local non-anthropogenic sources of PM_{10} (e.g. wind-blown soil and other crustal matter). Exposure estimates were assigned based on home address locations and averaged address-time-weighted PM_{10} exposure estimates in the whole pregnancy.[3]

Noise

Noise exposure was based on road traffic noise maps from the UK Department of Environment, Food and Rural Affairs (2006), which were generated under EC Directive 2002/49/EC (Assessment and Management of Environmental Noise) in the framework of the European Noise Directive (END). The primary noise indicators were Lden and Lnight, and these were obtained by doing an intersection between noise map and geocodes. Lden is the long-term average indicator designed to assess annoyance and defined by the END. It refers to an annual average of day, evening and night period of exposure. Lnight is the long-term average indicator designed to assess sleep disturbance and defined by the END. It refers to an annual average of night period of exposure.

Traffic

Traffic assessment was done using local layers of traffic from the UK Department of Environment, Food and Rural Affairs (DEFRA) from 2000. Inverse distance weighted (IDW) interpolation was used to predict values for any unmeasured locations. The inverse distance to nearest road was calculated.

Meteorological

The Long Ashton meteorological station was used to obtain all daily meteorological measurements for ALSPAC; this was part of the European Climate Assessment & Dataset project (ECAD). The meteorological data (temperature and relative humidity) were assigned to participants' geocodes inside a 50km buffer around the station. The average mean relative humidity, average mean temperature, average minimum temperature and average maximum temperature were calculated.

Built environment

Population density was characterised according to the Global Human Settlement Layer[4] from 1990, and corresponds to the number of inhabitants per square kilometre. Values were obtained by doing an intersection between population density grid maps and geocodes.

Building density was created based on the European Settlement Map 2017 and satellite images from 2010 to 2013, and corresponds to the sum of the built area divided by the buffer area around the residential address (both 100 and 300 metres were estimated). The values were generated as an intersection between a buffer geocode and the raster.

Street connectivity corresponds to the street network, which was obtained using the 2012 NAVTEQ. It was measured using the intersection density, which was defined as the number of intersections (that are not dead-ends) inside a buffer of 100 and 300 metres, divided by the area in

square kilometres of each buffer. A higher value indicates more intersections and a greater degree of connectivity enabling more direct travel between two points using existing streets and pathways.

Facilities were all points of interest for pedestrians as part of their daily life activities, like restaurants, shops, medical centres, schools, libraries etc, and were obtained using the 2012 NAVTEQ. A total of 100 different subcategories, grouped in 17 categories, were available in the NAVTEQ database. All categories, except Crossings, Auto Services and Parking were included. Facility richness index corresponds to the number of different facility types present divided by the maximum potential number of facility types specified in a buffer of 300 metres, and it ranges from 0 to 1. Facility density index corresponds to the number of facilities present divided by the area of 300 metres buffer (number of facilities/km^2). For both facility richness and facility density index, a higher value indicates a higher availability of different facility types.

Land use mix corresponds to the diversity of land use withing a given area, and it was derived from the Shannon's Evenness Index, using Urban Atlas database from 2006. The Shannon's Evenness Index is the degree of mixing of different types of land uses (such as residential, commercial, entertainment, and office development). A higher value indicates a more even distribution of land between the different types of land uses.

Main land use gives a percentage of all types of land use within an area of a buffer of 300 metres from each geocode. The following main land use categories were created, by grouping the land use categories available in the selected databases: "high density residential", "low density residential", "very low density residential", "industrial, commercial, public, military and private units", "transports", "port areas", "airport areas", "other", "urban green", "agricultural green", "natural green", "water".

Accessibility corresponds to public transport network and stops, and were obtained from Open Street Maps in 2019. Public transportation network density was calculated as meters of public transport lines (only bus lines) inside each 300 and 500 metre buffer, divided by the buffer area in square kilometres.

Walkability index was developed to quantify how "walkable" was a buffer of 300 metres around each geocode. The index included four components capturing differences in the physical environment (Land Use Shannon's Evenness Index; facility richness, population density, and connectivity index). Each component was converted to deciles before calculations. A higher value indicates a more walkable environment.

Natural spaces

Normalized Difference Vegetation Index (NDVI) was used to quantify vegetation by measuring the difference between near-infrared (which vegetation strongly reflects) and red light (which vegetation absorbs). NDVI derived from the Landsat 4–5 Thematic Mapper (TM), Landsat 7 Enhanced Thematic Mapper Plus (ETM+), and Landsat 8 Operational Land Imager (OLI)/Thermal Infrared Sensor (TIRS) with 30m x 30m resolution, from 1990, was used to determine the surrounding greenness. The imagery had been selected according to the following criteria: i) cloud cover less than 10 %, ii) Standard Terrain Correction (Level 1T) and iii) greenest period of the year. Two or more images were selected. Surrounding greenness was abstracted as the average of NDVI in buffers of 100, 300 and 500 meters around each geocode. Negative values in the images have been reclassified to null values. Furthermore, an indicator for residential proximity to major green spaces was created, and distance to the nearest green or blue major spaces and the area of this space were calculated. The Europe-wide "Urban Atlas" (prepared by European Environmental Protection Agency) from 2006 was used to extract maps of urban and natural green and blue spaces.

Unhealthy food environment

Unhealthy food environment exposure was generated based on the 2012 NAVTEQ database. The subcategories of facilities related to unhealthy food were selected among the 100 different subcategories in the NAVTEQ database. These were Auto Maintenance, Service, and Petrol (Petrol/Gasoline Station); Entertainment (Bar or Pub); Restaurants (Coffee Shop or Restaurant); Shopping (Convenience Store or Shopping). The unhealthy food environment corresponds to the number of unhealthy facilities present divided by the area of 300 meters buffer (number of facilities/m^2). A higher value indicates a higher availability of different unhealthy facilities.

Environmental exposures in replication cohorts

Environmental exposure data from GenR and EDEN were generated as part of the LifeCycle Project, and therefore identical methods to those used in ALSPAC were used for these cohorts. Details on the sources of data used can be found in the LifeCycle protocol <https://lifecycle-project.eu/wp-content/uploads/2021/07/Protocol_v4_2021_06_25.pdf>

In PANIC, measures of outdoor temperature, humidity and air pollution were available from the Finnish Meteorological Institute (FMI, https://en.ilmatieteenlaitos.fi/open-data). The participant's address at baseline was used as proxy of the address at birth. All the participants were linked to the same meteorological station close to the city centre of the city of Kuopio, the University of Eastern Finland and the Kuopio University Hospital.

In NFBC1986, measures of outdoor temperatures and humidity were available from the FMI. Using the Finnish Population Register, the addresses of the participants at birth were retrieved and whenever this was missing, the first address known during the first year of the child was selected. These geographical coordinates were then linked to the closest meteorological station and average temperature and humidity related to the month of birth, or the first month when an address was retrieved, were calculated.

Blood pressure (BP) measurement

In ALSPAC, a Dinamap 9300 Vital Signs Monitor was used at the 3-year clinic; a Dinamap 9301 Vital Signs Monitor (Morton Medical, London) was used at the 4-, 5-, 7-, 9-, and 11-year clinics; an Omron MI-5 was used at the 10-year clinic; a Dinamap 8100 Vital Signs Monitor (Morton Medical) was used at the 13-year clinic; and an Omron IntelliSense M6 (Omron Healthcare, Kyoto, Japan) was used at the 15-, and 18-year clinics; and an Omron 705 IT (Omron Electronic Components Europe BV) was used at the 24-year clinic. At age 24, study data were collected and managed using REDCap electronic data capture tools hosted at the University of Bristol.[5] REDCap (Research Electronic Data Capture) is a secure, web-based software platform designed to support data capture for research studies.

In EDEN, blood pressure was measured at 3 and 5 years of age by trained investigators following a standardised measurement protocol. SBP and DBP were measured using the COLIN 8800 oscillometer while the child was lying down after a five-minute rest period, three times spaced two minutes apart; and the last two measurements were averaged. The first measurement was discarded because a white coat effect was observed.

In GenR, child blood pressure was measured at the right brachial artery, four times with one-minute intervals, using a validated automatic sphygmomanometer Datascope Accutor Plus (Paramus, NJ, USA) with an appropriate cuff. We calculated the mean value for systolic (SBP) and diastolic blood pressure (DBP) using the last three measurements of each participant.

In PANIC, blood pressure was measured at baseline (6–8 years of age), at 2-year follow-up (8–10 years of age) and at 8-year follow-up (15–17 years of age). A research nurse measured SBP and DBP from the right arm using the Heine Gamma® G7 aneroid sphygmomanometer (Heine Optotechnik, Herrsching, Germany) to accuracy of 2 mmHg. The measurement protocol included, after a rest of 5 minutes, three measurements in the sitting position at 2-minute intervals. The mean of all three values was used.

In NFBC1986, blood pressure was measured with the participant sitting on a chair after 15 minutes of rest, a cuff of the appropriate size was placed on his/her right upper arm. Blood pressure was measured with an automatic oscillometer blood pressure meter (Omron 705CP). The measure

was repeated 2 minutes later; a third measure was done if the second measure failed. If this failed, the nurse used a mercury sphygmomanometer. The mean of first and second measures (or first and third measures, if second failed) were used.

Confounders

The confounders used in this study were harmonised as part of the LifeCycle Project. More information on the harmonisation protocol can be found in <https://lifecycle-project.eu/for-scientists/guides-manuals/>.

Maternal level of education was based on the highest ongoing or completed education and classified as low, medium and high according to the International Standard Classification of Education 97/2011 (ISCED-97/2011). High education corresponded to short cycle tertiary, Bachelor, Masters, Doctoral or equivalent (ISCED2011: 5-8, ISCED-97: 5-6); medium education corresponded to upper secondary, or post-secondary non-tertiary (ISCED-2011: 3-4, ISCED-97: 3-4); and low education corresponded to no education; early childhood; pre-primary; primary; lower secondary or second stage of basic education (ISCED-2011: 0-2, ISCED-97: 0-2).

Maternal age at delivery was calculated from the mother's date of birth (obtained at enrolment) and the date of delivery. Ethnicity was self-reported at enrolment and recoded into White and non-White, since 97.5% of women were White.

Area deprivation in ALSPAC was based on data from the Ministry of Housing, Communities & Local Government (UK Government) from 2015 and linked to geocoded addresses at birth. It combined weighted information from seven domains to produce an overall relative measure of deprivation. The domains and their weights were the following: income deprivation (22.5%), employment deprivation (22.5%), education, skills and training deprivation (13.5%), health deprivation and disability (13.5%), crime (9.3%), barriers to housing and services (9.3%), and living environment deprivation (9.3%). It was categorised in quintiles, where the first corresponds to the least deprived and the last to the most deprived.

Confounders used in the replication analysis

We tried to adjust the replication analyses for the same set of covariates used in the main analysis. However, not all cohorts had information for all confounders. In EDEN and GenR, all confounders were available. In PANIC, analyses were adjusted for sex, maternal education, maternal age at birth, and maternal ethnicity. In NFBC1986, analyses were adjusted for sex, maternal education and maternal age at birth.

In EDEN, given the different levels in the exposures and outcomes in both cities where the data were collected (Nancy and Poitiers), the cohort was stratified by city.

Selection of linear splines knots

We fitted the best-fitting curve for SBP and DBP trajectories using fractional polynomials to visualise the possible knots for the linear splines. The best-fitting function was selected from a family of flexible polynomial functions. In brief, with simple (single-level) linear regression, the model deviance of each of eight powers (–2, –1, –0.5, 0, 0.5, 1, 2, 3) was used to identify the best-fitting single polynomial. All possible combinations of pairs of these polynomials were then examined, and again the model deviance was used to select the best-fitting model containing two powers (the one with the lowest deviance). For both SBP and DBP models, the best-fitting curve had both powers of 3.

We aimed to fit knots which would represent three developmental periods: childhood, adolescence and emerging adulthood. The location was selected by comparing the Akaike Information Criterion (AIC) and Bayesian Information Criterion (BIC) of different models with knots placed around ages 10 and 18 years. The best model had knots placed at 9.9 and 17.9 years (Supplemental Figure 2).

References

1. Boyd A, Thomas R, Hansell AL et al. Data Resource Profile: The ALSPAC birth cohort as a platform to study the relationship of environment and health and social factors. Int J Epidemiol. 2019;48(4):1038-1039k.
2. de Hoogh K, Chen J, Gulliver J et al. Spatial PM(2.5), NO(2), O(3) and BC models for Western Europe - Evaluation of spatiotemporal stability. Environ Int. 2018;120:81-92.
3. Gulliver J, Elliott P, Henderson J et al. Local- and regional-scale air pollution modelling (PM10) and exposure assessment for pregnancy trimesters, infancy, and childhood to age 15years: Avon Longitudinal Study of Parents And Children (ALSPAC). Environ Int. 2018;113:10-19.
4. European Commission, Joint Research Centre; Columbia University, Center for International Earth Science Information Network (2015): GHS population grid, derived from GPW4, multitemporal (1975, 1990, 2000, 2015). European Commission, Joint Research Centre (JRC) [Dataset] PID: http://data.europa.eu/89h/jrc-ghsl-ghs_pop_gpw4_globe_r2015a.
5. Harris PA, Taylor R, Thielke R, Payne J, Gonzalez N, Conde JG. Research electronic data capture (REDCap)--a metadata-driven methodology and workflow process for providing translational research informatics support. J Biomed Inform. 2009;42(2):377-81.

Supplemental results

Supplemental Table 1. Description of the urban environmental exposures assessed in the Avon Longitudinal Study of Parents and Children (ALSPAC)

Type of exposure	Variable name	Definition (unit)	Type of variable
Noise	lden_c	Average noise exposure of day, evening and night period (Lden, dB)	Categorical
	ln_c	Average noise exposure of night period (Lnight, dB)	Categorical
Traffic	distinvnear1	Inverse distance to nearest road (1/m)	Continuous
Meteorological	hum	Relative humidity (%)	Continuous
	tm	Mean temperature (Celsius)	Continuous
	tmax	Maximum temperature (Celsius)	Continuous
	tmin	Minimum temperature (Celsius)	Continuous
Built environment	bdens100	Building density within 100m buffer (m^2 built/km^2)	Continuous
	bdens300	Building density within 300m buffer (m^2 built/km^2)	Continuous
	connind100	Connectivity within 100m buffer (N intersections/km^2)	Continuous
	connind300	Connectivity within 300m buffer (N intersections/km^2)	Continuous
	fdensity300	Facility density within 300m buffer (N facilities/km^2)	Continuous
	frichness300	Facility richness index within 300m buffer	Continuous
	landuseshan300	Land use within 300m buffer (%)	Continuous
	agrgr	Agricultural area within 300m buffer (%)	Continuous
	airpt	Airport within 300m buffer (%)	Binary
	hdres	Continuous urban fabric within 300m buffer (%)	Continuous
	indtr	Industrial, commercial, public, military and private units within 300m buffer (%)	Continuous

	ldres	Discontinuous low density urban fabric within 300m buffer (%)	Continuous
	natgr	Forests within 300m buffer (%)	Categorical
	other	Mineral extraction and dump sites, constructions within 300m buffer (%)	Categorical
	port	Port areas within 300m buffer (%)	Binary
	trans	Transport networks and other constructed hard-surfaced areas within 300m buffer (%)	Continuous
	urbgr	Green urban areas within 300m buffer (%)	Continuous
	vldres	Discontinuous very low density urban fabric within 300m buffer (%)	Binary
	water	Water land use within 300m buffer (%)	Binary
	popdens	Population density (inhabitants/km^2)	Continuous
	bus_lines_300	Length of bus lines in 300m buffer (m/km^2)	Continuous
	bus_lines_500	Length of bus lines in 500m buffer (m/km^2)	Continuous
	walkability_mean	Walkability index	Continuous
Natural spaces	blue_dist	Distance to closest blue space (m)	Continuous
	blue_size_quint	Area of closest blue space >5,000m^2 (quintiles)	Categorical
	blueyn300	Existence of blue space within 300m buffer	Binary
	green_dist	Distance to closest green space >5,000m^2 (m)	Continuous
	green_size	Area of closest green space >5,000m^2 (m^2)	Continuous
	greenyn300	Existence of green space >5,000m^2 within 300m buffer	Binary
	ndvi100	Average greenness within 100m buffer (normalized difference vegetation index [NDVI])	Categorical
	ndvi300	Average greenness within 300m buffer (NDVI)	Categorical

	ndvi500	Average greenness within 500m buffer (NDVI)	Categorical
Food environment	foodenvdens300	Food facilities density within 300m buffer (number of facilities related to unhealthy food/km^2)	Continuous
Air pollution	no2	Nitrogen dioxide (NO_2, µg/m^3)	Continuous
	pm25	Particulate matter <2.5µm ($PM_{2.5}$, µg/m^3)	Continuous
	pm10	Particulate matter <10µm (PM_{10}, µg/m^3)	Continuous

Supplemental Table 2. Baseline characteristics of the ALSPAC participants included (n=7,454) and not included in the analysis (n=1,780) due to missing data on one or more of the confounders

Variable	Included	Not included	p-value
	N (%) or mean (SD)		
Sex			0.593
Males	3,708 (49.7%)	898 (50.4%)	
Females	3,746 (50.3%)	882 (49.5%)	
Maternal education			<0.001
Low	1,080 (14.5%)	289 (22.7%)	
Medium	5,237 (70.3%)	837 (65.9%)	
High	1,137 (15.3%)	145 (11.4%)	
Ethnicity			0.023
White	7,302 (98.0%)	1,204 (96.9%)	
Non-White	152 (2.0%)	38 (3.1%)	
Area deprivation			<0.001
1st quintile (least deprived)	1,434 (19.2%)	105 (11.4%)	
2nd quintile	1,391 (18.7%)	110 (12.0%)	
3rd quintile	1,677 (22.5%)	179 (19.5%)	
4th quintile	1,462 (19.6%)	207 (22.5%)	
5th quintile (most deprived)	1,490 (20.0%)	318 (34.6%)	
Age of mother at birth (years), mean (SD)	28.9 (4.6)	28.4 (5.1)	0.005

Supplemental Table 3. Distribution of age, systolic and diastolic blood pressure in each assessment in the ALSPAC participants included in the analysis (n=7,454)

Assessment	N	Age (years) Mean (SD)	SBP (mmHg) Mean (SD)	DBP (mmHg) Mean (SD)
1	874	3.1 (0.02)	90.2 (8.02)	56.0 (6.29)
2	835	4.1 (0.03)	94.7 (7.83)	57.4 (6.12)
3	800	5.2 (0.06)	98.8 (7.88)	59.7 (6.45)
4	6,056	7.5 (0.18)	98.7 (9.11)	56.4 (6.61)
5	5,811	9.9 (0.31)	102.7 (9.27)	57.4 (6.37)
6	5,522	10.6 (0.25)	104.2 (9.02)	60.2 (7.91)
7	5,387	11.7 (0.23)	105.5 (9.50)	58.7 (6.36)
8	5,185	12.8 (0.23)	111.4 (9.64)	56.7 (7.85)
9	4,093	15.4 (0.33)	122.8 (10.93)	67.4 (8.77)
10	3,585	17.7 (0.37)	116.5 (9.86)	64.1 (5.86)
11	3,070	24.4 (0.79)	115.9 (11.40)	66.8 (8.01)

DBP: diastolic blood pressure; SBP: systolic blood pressure; SD: standard deviation

Supplemental Table 4. Distribution of the urban environmental exposures in ALSPAC

**Environmental exposure**	**N (%) or Median (IQR)**
Lden (dB)	
<55	1,561 (30.6%)
55-59.9	2,397 (47.0%)
60-64.9	1,046 (20.5%)
65-69.9	75 (1.5%)
≥70	16 (0.3%)
Lnight (dB)	
<55	3,463 (68.0%)
55-59.9	1,446 (28.4%)
60-64.9	161 (3.2%)
≥65	25 (0.5%)
Inverse distance to nearest road (inverse m), median (IQR)	0.06 (0.03)
Humidity (relative %), median (IQR)	81.9 (2.0)
Mean temperature (Celsius), median (IQR)	9.5 (2.4)
Building density within 100m buffer (m^2 built/km^2), median (IQR)	450000 (180000)
Building density within 300m buffer (m^2 built/km^2), median (IQR)	410000 (180000)
Connectivity within 100m buffer (N intersections/km^2), median (IQR)	130 (90)
Connectivity within 300m buffer (N intersections/km^2), median (IQR)	120 (70)
Facility density within 300m buffer (N facilities/km^2), median (IQR)	18 (32)
Land use within 300m buffer (%), median (IQR)	0.44 (0.13)
Agricultural area within 300m buffer (%), median (IQR)	0 (4)
Airport within 300m buffer (%)	
0	7,202 (99.4%)
>0	44 (0.6%)
Continuous Urban Fabric within 300m buffer (%)	
≤1	6,007 (82.9%)
2-5	578 (8.0%)
6-9	251 (3.5%)
10-14	131 (1.8%)
15+	279 (3.9%)
Industrial, commercial, public, military and private units within 300m buffer (%), median (IQR)	6 (10)
Discontinuous low density urban fabric within 300m buffer (%), median (IQR)	64 (24)
Forests within 300m buffer (%)	
0	6,607 (91.2%)
1-4	232 (3.2%)
5-10	227 (3.1%)
>10	180 (2.5%)
Mineral extraction and dump sites, constructions within 300m buffer (%)	
0	5,934 (81.9%)
1-4	927 (12.8%)
5-10	385 (5.3%)
Port areas within 300m buffer (%)	
0	7225 (99.7%)

>0	21 (0.3%)
Transport networks and other constructed hard-surfaced areas within 300m buffer (%), median (IQR)	10 (4)
Green urban areas within 300m buffer (%), median (IQR)	6 (12)
Discontinuous very low density urban fabric within 300m buffer (%)	
0	6,826 (94.2%)
>0	420 (5.8%)
Population density (inhabitants/km^2), median (IQR)	4300 (4300)
Bus lines in 300m buffer (m/km^2), median (IQR)	2900 (2000)
Bus lines in 500m buffer (m/km^2), median (IQR)	2800 (1800)
Walkability index, median (IQR)	0.28 (0.08)
Distance to closest blue space (m), median (IQR)	1300 (1200)
Area of closest blue space >5,000m^2 (m^2)	
1st quintile (lowest)	1,563 (21.6%)
2nd quintile	1,464 (20.2%)
3rd quintile	1,754 (24.2%)
4th quintile	1,411 (19.5%)
5th quintile (highest)	1,048 (14.5%)
Existence of blue space within 300m buffer	
No	6,769 (93.5%)
Yes	474 (6.5%)
Distance to closest green space >5,000m^2 (m), median (IQR)	162.17 (215.61)
Area of closest green space >5,000m^2 (m^2), median (IQR)	40000 (120000)
Existence of green space >5,000m^2 within 300m buffer	
No	1,761 (24.3%)
Yes	5,482 (75.7%)
Average greenness within 100m buffer (NDVI)	
0.2	567 (7.6%)
0.3	1,808 (24.3%)
0.4	3,506 (47.0%)
0.5	1,248 (16.7%)
0.6	291 (3.9%)
0.7	34 (0.5%)
Average greenness within 300m buffer (NDVI)	
0.2	316 (4.2%)
0.3	1,230 (16.5%)
0.4	3,724 (50.0%)
0.5	1,736 (23.3%)
0.6	395 (5.3%)
0.7	53 (0.7%)
Average greenness within 500m buffer (NDVI)	
0.2	227 (3.0%)
0.3	973 (13.1%)
0.4	3,606 (48.4%)
0.5	2,119 (28.4%)
0.6	466 (6.3%)
0.7	63 (0.8%)

Unhealthy food facilities density within 300m buffer (N facilities/km^2), median (IQR)	4 (7)
NO_2 ($\mu g/m^3$), median (IQR)	27.2 (5.2)
$PM_{2.5}$ ($\mu g/m^3$), median (IQR)	13.3 (1.1)
PM_{10} ($\mu g/m^3$), median (IQR)	20.7 (3.8)

Supplemental Table 5. Adjusted mean trajectories of systolic and diastolic blood pressure from childhood to early adulthood in ALSPAC

	**Mean (95%CI)**
**Systolic blood pressure (SBP)**	
Mean SBP at 3y, mmHg	94.3 (89.9, 92.7)
Change in SBP in childhood (3 to 10y), mmHg/y	1.56 (1.50, 1.62)
Mean SBP at 10y, mmHg	101.8 (101.1, 102.5)
Change in SBP in adolescence (10 to 18y), mmHg/y	2.57 (2.53, 2.61)
Mean SBP at 18y, mmHg	122.3 (121.6, 123.1)
Change in SBP in early adulthood (18 to 24y), mmHg/y	-1.02 (-1.08, -0.97)
Ma at 24y, mmHg	116.2 (115.5, 117.0)
**Diastolic blood pressure (DBP)**	
DBP at 3y, mmHg	54.9 (53.9, 55.9)
Change in DBP in childhood (3 to 10y), mmHg/y	0.17 (0.12, 0.21)
DBP at 10y, mmHg	55.8 (55.3, 56.3)
Change in DBP in adolescence (10 to 18y), mmHg/y	1.06 (1.03, 1.09)
Blood pressure at 18y, mmHg	64.3 (63.8, 64.7)
Change in DBP in early adulthood (18 to 24y), mmHg/y	0.14 (0.09, 0.18)
Blood pressure at 24y, mmHg	65.1 (64.6, 65.6)

Adjusted for maternal education, age at delivery, ethnicity, area deprivation, and sex. All covariates used in the adjustment were set to the mean value or the reference category: maternal education (high), age at delivery (28.9 years), ethnicity (White), area deprivation (least deprived), and sex (male).

Supplemental Table 6. Associations between urban environmental exposures and changes in systolic blood pressure (SBP) in ALSPAC

Exposure	Parameter	N	N obs	beta (95% CI)	p-value
Lden	mean difference in SBP at age 3y (mmHg)	5,095	28,132	0.14 (-0.39, 0.66)	0.610
	change in SBP in childhood (mmHg/y): 3 to 10y	5,095	28,132	-0.01 (-0.10, 0.08)	0.792
	change in SBP in adolescence (mmHg/y): 10 to 18y	5,095	28,132	0.02 (-0.04, 0.09)	0.517
	change in SBP in adulthood (mmHg/y): 18 to 26y	5,095	28,132	-0.06 (-0.15, 0.03)	0.170
	change in SBP from childhood to early adulthood				0.590
Lnight	mean difference in SBP at age 3y (mmHg)	5,095	28,132	-0.13 (-0.85, 0.59)	0.715
	change in SBP in childhood (mmHg/y): 3 to 10y	5,095	28,132	0.03 (-0.09, 0.16)	0.576
	change in SBP in adolescence (mmHg/y): 10 to 18y	5,095	28,132	0.05 (-0.04, 0.13)	0.320
	change in SBP in adulthood (mmHg/y): 18 to 26y	5,095	28,132	-0.08 (-0.20, 0.05)	0.218
	change in SBP from childhood to early adulthood				0.429
Inverse distance to nearest road	mean difference in SBP at age 3y (mmHg)	7,454	41,214	-0.13 (-0.49, 0.22)	0.470
	change in SBP in childhood (mmHg/y): 3 to 10y	7,454	41,214	0.01 (-0.05, 0.07)	0.718
	change in SBP in adolescence (mmHg/y): 10 to 18y	7,454	41,214	0.01 (-0.04, 0.05)	0.822
	change in SBP in adulthood (mmHg/y): 18 to 26y	7,454	41,214	-0.04 (-0.10, 0.02)	0.198
	change in SBP from childhood to early adulthood				0.599
Humidity	mean difference in SBP at age 3y (mmHg)	7,402	40,925	-1.97 (-2.53, -1.41)	4.73E-12
	change in SBP in childhood (mmHg/y): 3 to 10y	7,402	40,925	0.29 (0.20, 0.39)	9.94E-10
	change in SBP in adolescence (mmHg/y): 10 to 18y	7,402	40,925	0.06 (0.00, 0.13)	0.059
	change in SBP in adulthood (mmHg/y): 18 to 26y	7,402	40,925	-0.11 (-0.20, -0.03)	0.010
	change in SBP from childhood to early adulthood				3.51E-13
Mean temperature	mean difference in SBP at age 3y (mmHg)	7,413	40,988	0.89 (0.28, 1.50)	0.004
	change in SBP in childhood (mmHg/y): 3 to 10y	7,413	40,988	-0.17 (-0.28, -0.07)	8.55E-04
	change in SBP in adolescence (mmHg/y): 10 to 18y	7,413	40,988	0.01 (-0.06, 0.09)	0.698
	change in SBP in adulthood (mmHg/y): 18 to 26y	7,413	40,988	0.02 (-0.08, 0.12)	0.653
	change in SBP from childhood to early adulthood				0.007
Building density in 100m buffer	mean difference in SBP at age 3y (mmHg)	7,447	41,172	-0.06 (-0.49, 0.36)	0.768
	change in SBP in childhood (mmHg/y): 3 to 10y	7,447	41,172	-0.05 (-0.12, 0.02)	0.201
	change in SBP in adolescence (mmHg/y): 10 to 18y	7,447	41,172	0.02 (-0.04, 0.07)	0.559

	change in SBP in adulthood (mmHg/y): 18 to 26y	7,447	41,172	0.02 (-0.05, 0.09)	0.535
	change in SBP from childhood to early adulthood				0.511
Building density in 300m buffer	mean difference in SBP at age 3y (mmHg)	7,450	41,191	-0.13 (-0.55, 0.30)	0.559
	change in SBP in childhood (mmHg/y): 3 to 10y	7,450	41,191	-0.02 (-0.09, 0.05)	0.529
	change in SBP in adolescence (mmHg/y): 10 to 18y	7,450	41,191	0.01 (-0.04, 0.06)	0.743
	change in SBP in adulthood (mmHg/y): 18 to 26y	7,450	41,191	0.02 (-0.05, 0.08)	0.562
	change in SBP from childhood to early adulthood				0.823
Connectivity in 100m buffer	mean difference in SBP at age 3y (mmHg)	7,075	38,939	-0.06 (-0.43, 0.30)	0.730
	change in SBP in childhood (mmHg/y): 3 to 10y	7,075	38,939	-0.01 (-0.07, 0.05)	0.765
	change in SBP in adolescence (mmHg/y): 10 to 18y	7,075	38,939	-0.02 (-0.06, 0.03)	0.499
	change in SBP in adulthood (mmHg/y): 18 to 26y	7,075	38,939	0.03 (-0.03, 0.09)	0.308
	change in SBP from childhood to early adulthood				0.694
Connectivity in 300m buffer	mean difference in SBP at age 3y (mmHg)	7,432	41,071	0.11 (-0.30, 0.52)	0.600
	change in SBP in childhood (mmHg/y): 3 to 10y	7,432	41,071	-0.05 (-0.12, 0.02)	0.160
	change in SBP in adolescence (mmHg/y): 10 to 18y	7,432	41,071	0.02 (-0.03, 0.07)	0.442
	change in SBP in adulthood (mmHg/y): 18 to 26y	7,432	41,071	0.01 (-0.05, 0.08)	0.698
	change in SBP from childhood to early adulthood				0.487
Facility density within 300m buffer	mean difference in SBP at age 3y (mmHg)	7,453	41,213	-0.03 (-0.28, 0.22)	0.791
	change in SBP in childhood (mmHg/y): 3 to 10y	7,453	41,213	-0.02 (-0.07, 0.02)	0.265
	change in SBP in adolescence (mmHg/y): 10 to 18y	7,453	41,213	0.01 (-0.02, 0.04)	0.395
	change in SBP in adulthood (mmHg/y): 18 to 26y	7,453	41,213	0.00 (-0.04, 0.03)	0.812
	change in SBP from childhood to early adulthood				0.693
Land use within 300m buffer	mean difference in SBP at age 3y (mmHg)	7,246	39,975	0.07 (-0.39, 0.54)	0.758
	change in SBP in childhood (mmHg/y): 3 to 10y	7,246	39,975	-0.01 (-0.09, 0.07)	0.812
	change in SBP in adolescence (mmHg/y): 10 to 18y	7,246	39,975	-0.02 (-0.08, 0.04)	0.501
	change in SBP in adulthood (mmHg/y): 18 to 26y	7,246	39,975	-0.04 (-0.12, 0.03)	0.260
	change in SBP from childhood to early adulthood				0.390
Agricultural area within 300m buffer	mean difference in SBP at age 3y (mmHg)	7,246	39,975	-0.02 (-0.12, 0.08)	0.689
	change in SBP in childhood (mmHg/y): 3 to 10y	7,246	39,975	0.00 (-0.02, 0.02)	0.943
	change in SBP in adolescence (mmHg/y): 10 to 18y	7,246	39,975	0.00 (-0.01, 0.01)	0.838
	change in SBP in adulthood (mmHg/y): 18 to 26y	7,246	39,975	0.00 (-0.02, 0.01)	0.889

	change in SBP from childhood to early adulthood				0.995
Airport within 300m buffer	mean difference in SBP at age 3y (mmHg)	7,246	39,975	-2.09 (-6.58, 2.40)	0.362
	change in SBP in childhood (mmHg/y): 3 to 10y	7,246	39,975	0.46 (-0.29, 1.21)	0.226
	change in SBP in adolescence (mmHg/y): 10 to 18y	7,246	39,975	-0.03 (-0.56, 0.50)	0.911
	change in SBP in adulthood (mmHg/y): 18 to 26y	7,246	39,975	0.17 (-0.47, 0.80)	0.606
	change in SBP from childhood to early adulthood				0.579
Continuous Urban Fabric within 300m buffer	mean difference in SBP at age 3y (mmHg)	7,246	39,975	-0.29 (-0.67, 0.09)	0.136
	change in SBP in childhood (mmHg/y): 3 to 10y	7,246	39,975	0.01 (-0.06, 0.07)	0.855
	change in SBP in adolescence (mmHg/y): 10 to 18y	7,246	39,975	0.01 (-0.03, 0.06)	0.608
	change in SBP in adulthood (mmHg/y): 18 to 26y	7,246	39,975	0.00 (-0.06, 0.06)	0.972
	change in SBP from childhood to early adulthood				0.928
Industrial, commercial, public, military and private units within 300m buffer	mean difference in SBP at age 3y (mmHg)	7,246	39,975	-0.05 (-0.41, 0.31)	0.780
	change in SBP in childhood (mmHg/y): 3 to 10y	7,246	39,975	0.03 (-0.03, 0.09)	0.335
	change in SBP in adolescence (mmHg/y): 10 to 18y	7,246	39,975	-0.04 (-0.08, 0.01)	0.115
	change in SBP in adulthood (mmHg/y): 18 to 26y	7,246	39,975	-0.01 (-0.07, 0.05)	0.795
	change in SBP from childhood to early adulthood				0.330
Discontinuous Low Density Urban Fabric within 300m buffer	mean difference in SBP at age 3y (mmHg)	7,246	39,975	-0.12 (-0.60, 0.36)	0.622
	change in SBP in childhood (mmHg/y): 3 to 10y	7,246	39,975	0.01 (-0.07, 0.09)	0.822
	change in SBP in adolescence (mmHg/y): 10 to 18y	7,246	39,975	0.01 (-0.05, 0.07)	0.800
	change in SBP in adulthood (mmHg/y): 18 to 26y	7,246	39,975	0.01 (-0.06, 0.09)	0.743
	change in SBP from childhood to early adulthood				0.937
Forests within 300m buffer	mean difference in SBP at age 3y (mmHg)	7,246	39,975	-0.24 (-0.80, 0.33)	0.408
	change in SBP in childhood (mmHg/y): 3 to 10y	7,246	39,975	0.01 (-0.09, 0.10)	0.901
	change in SBP in adolescence (mmHg/y): 10 to 18y	7,246	39,975	0.03 (-0.04, 0.10)	0.386
	change in SBP in adulthood (mmHg/y): 18 to 26y	7,246	39,975	-0.01 (-0.10, 0.09)	0.864
	change in SBP from childhood to early adulthood				0.794
Mineral extraction and dump sites, constructions within 300m buffer	mean difference in SBP at age 3y (mmHg)	7,246	39,975	-0.56 (-1.18, 0.07)	0.079
	change in SBP in childhood (mmHg/y): 3 to 10y	7,246	39,975	0.14 (0.03, 0.24)	0.011
	change in SBP in adolescence (mmHg/y): 10 to 18y	7,246	39,975	-0.03 (-0.10, 0.05)	0.526
	change in SBP in adulthood (mmHg/y): 18 to 26y	7,246	39,975	0.05 (-0.06, 0.15)	0.398
	change in SBP from childhood to early adulthood				0.063

Port areas within 300m buffer	mean difference in SBP at age 3y (mmHg)	7,246	39,975	5.33 (-1.9, 12.56)	0.149
	change in SBP in childhood (mmHg/y): 3 to 10y	7,246	39,975	-0.65 (-1.87, 0.56)	0.291
	change in SBP in adolescence (mmHg/y): 10 to 18y	7,246	39,975	0.08 (-0.76, 0.91)	0.852
	change in SBP in adulthood (mmHg/y): 18 to 26y	7,246	39,975	-0.14 (-1.54, 1.27)	0.849
	change in SBP from childhood to early adulthood				0.751
Transport networks and other constructed hard-surfaced areas within 300m buffer	mean difference in SBP at age 3y (mmHg)	7,246	39,975	0.04 (-0.38, 0.45)	0.862
	change in SBP in childhood (mmHg/y): 3 to 10y	7,246	39,975	-0.02 (-0.09, 0.05)	0.500
	change in SBP in adolescence (mmHg/y): 10 to 18y	7,246	39,975	0.01 (-0.04, 0.06)	0.566
	change in SBP in adulthood (mmHg/y): 18 to 26y	7,246	39,975	-0.02 (-0.08, 0.05)	0.651
	change in SBP from childhood to early adulthood				0.882
Green urban areas within 300m buffer	mean difference in SBP at age 3y (mmHg)	7,246	39,975	0.35 (-0.06, 0.75)	0.095
	change in SBP in childhood (mmHg/y): 3 to 10y	7,246	39,975	-0.02 (-0.09, 0.05)	0.527
	change in SBP in adolescence (mmHg/y): 10 to 18y	7,246	39,975	-0.02 (-0.07, 0.03)	0.393
	change in SBP in adulthood (mmHg/y): 18 to 26y	7,246	39,975	0.00 (-0.07, 0.07)	0.984
	change in SBP from childhood to early adulthood				0.578
Discontinuous very low density urban fabric within 300m buffer	mean difference in SBP at age 3y (mmHg)	7,246	39,975	-0.61 (-2.15, 0.92)	0.433
	change in SBP in childhood (mmHg/y): 3 to 10y	7,246	39,975	0.03 (-0.22, 0.29)	0.797
	change in SBP in adolescence (mmHg/y): 10 to 18y	7,246	39,975	0.14 (-0.04, 0.31)	0.123
	change in SBP in adulthood (mmHg/y): 18 to 26y	7,246	39,975	-0.13 (-0.36, 0.10)	0.263
	change in SBP from childhood to early adulthood				0.322
Population density	mean difference in SBP at age 3y (mmHg)	7,308	40,347	-0.02 (-0.47, 0.43)	0.930
	change in SBP in childhood (mmHg/y): 3 to 10y	7,308	40,347	-0.06 (-0.13, 0.02)	0.140
	change in SBP in adolescence (mmHg/y): 10 to 18y	7,308	40,347	0.02 (-0.03, 0.08)	0.402
	change in SBP in adulthood (mmHg/y): 18 to 26y	7,308	40,347	0.08 (0.01, 0.15)	0.031
	change in SBP from childhood to early adulthood				0.032
Bus lines in 300m buffer	mean difference in SBP at age 3y (mmHg)	6,800	37,493	0.52 (0.08, 0.97)	0.022
	change in SBP in childhood (mmHg/y): 3 to 10y	6,800	37,493	-0.06 (-0.13, 0.02)	0.131
	change in SBP in adolescence (mmHg/y): 10 to 18y	6,800	37,493	-0.02 (-0.08, 0.04)	0.479
	change in SBP in adulthood (mmHg/y): 18 to 26y	6,800	37,493	-0.03 (-0.11, 0.05)	0.436
	change in SBP from childhood to early adulthood				0.124
Bus lines in 500m buffer	mean difference in SBP at age 3y (mmHg)	7,226	39,897	0.27 (-0.19, 0.73)	0.252

	change in SBP in childhood (mmHg/y): 3 to 10y	7,226	39,897	-0.05 (-0.13, 0.03)	0.193
	change in SBP in adolescence (mmHg/y): 10 to 18y	7,226	39,897	-0.01 (-0.07, 0.05)	0.803
	change in SBP in adulthood (mmHg/y): 18 to 26y	7,226	39,897	-0.03 (-0.10, 0.05)	0.499
	change in SBP from childhood to early adulthood				0.360
Walkability index	mean difference in SBP at age 3y (mmHg)	7,224	39,832	0.04 (-0.37, 0.46)	0.843
	change in SBP in childhood (mmHg/y): 3 to 10y	7,224	39,832	-0.04 (-0.11, 0.03)	0.244
	change in SBP in adolescence (mmHg/y): 10 to 18y	7,224	39,832	0.01 (-0.04, 0.06)	0.829
	change in SBP in adulthood (mmHg/y): 18 to 26y	7,224	39,832	0.01 (-0.06, 0.07)	0.859
	change in SBP from childhood to early adulthood				0.693
Distance to closest blue space	mean difference in SBP at age 3y (mmHg)	7,243	39,952	0.27 (-0.16, 0.70)	0.221
	change in SBP in childhood (mmHg/y): 3 to 10y	7,243	39,952	-0.03 (-0.1, 0.05)	0.467
	change in SBP in adolescence (mmHg/y): 10 to 18y	7,243	39,952	-0.04 (-0.09, 0.01)	0.146
	change in SBP in adulthood (mmHg/y): 18 to 26y	7,243	39,952	0.06 (-0.02, 0.13)	0.127
	change in SBP from childhood to early adulthood				0.172
Size of blue space	mean difference in SBP at age 3y (mmHg)	7,240	39,937	-0.05 (-0.30, 0.20)	0.718
	change in SBP in childhood (mmHg/y): 3 to 10y	7,240	39,937	0.00 (-0.04, 0.04)	0.955
	change in SBP in adolescence (mmHg/y): 10 to 18y	7,240	39,937	0.00 (-0.03, 0.03)	0.993
	change in SBP in adulthood (mmHg/y): 18 to 26y	7,240	39,937	0.01 (-0.04, 0.05)	0.788
	change in SBP from childhood to early adulthood				0.993
Existence blue space within 300m buffer	mean difference in SBP at age 3y (mmHg)	7,243	39,952	0.77 (-0.63, 2.17)	0.282
	change in SBP in childhood (mmHg/y): 3 to 10y	7,243	39,952	-0.11 (-0.34, 0.13)	0.383
	change in SBP in adolescence (mmHg/y): 10 to 18y	7,243	39,952	0.12 (-0.05, 0.30)	0.164
	change in SBP in adulthood (mmHg/y): 18 to 26y	7,243	39,952	-0.13 (-0.35, 0.09)	0.254
	change in SBP from childhood to early adulthood				0.468
Distance to closest green space	mean difference in SBP at age 3y (mmHg)	7,243	39,952	-0.46 (-0.93, 0.01)	0.053
	change in SBP in childhood (mmHg/y): 3 to 10y	7,243	39,952	0.06 (-0.02, 0.14)	0.127
	change in SBP in adolescence (mmHg/y): 10 to 18y	7,243	39,952	-0.02 (-0.08, 0.04)	0.474
	change in SBP in adulthood (mmHg/y): 18 to 26y	7,243	39,952	0.04 (-0.04, 0.12)	0.292
	change in SBP from childhood to early adulthood				0.354
Size of green space	mean difference in SBP at age 3y (mmHg)	7,243	39,952	-0.05 (-0.51, 0.42)	0.843
	change in SBP in childhood (mmHg/y): 3 to 10y	7,243	39,952	0.03 (-0.04, 0.11)	0.391

	change in SBP in adolescence (mmHg/y): 10 to 18y	7,243	39,952	-0.03 (-0.09, 0.02)	0.258
	change in SBP in adulthood (mmHg/y): 18 to 26y	7,243	39,952	0.03 (-0.04, 0.11)	0.373
	change in SBP from childhood to early adulthood				0.614
Existence green space within 300m buffer	mean difference in SBP at age 3y (mmHg)	7,243	39,952	0.66 (-0.13, 1.44)	0.101
	change in SBP in childhood (mmHg/y): 3 to 10y	7,243	39,952	-0.09 (-0.22, 0.04)	0.167
	change in SBP in adolescence (mmHg/y): 10 to 18y	7,243	39,952	0.01 (-0.09, 0.10)	0.917
	change in SBP in adulthood (mmHg/y): 18 to 26y	7,243	39,952	-0.03 (-0.16, 0.10)	0.635
	change in SBP from childhood to early adulthood				0.486
Average greenness within 100m buffer	mean difference in SBP at age 3y (mmHg)	7,454	41,214	2.94 (-0.63, 6.52)	0.107
	change in SBP in childhood (mmHg/y): 3 to 10y	7,454	41,214	-0.23 (-0.83, 0.36)	0.441
	change in SBP in adolescence (mmHg/y): 10 to 18y	7,454	41,214	0.25 (-0.18, 0.69)	0.250
	change in SBP in adulthood (mmHg/y): 18 to 26y	7,454	41,214	0.07 (-0.49, 0.64)	0.799
	change in SBP from childhood to early adulthood				0.584
Average greenness within 300m buffer	mean difference in SBP at age 3y (mmHg)	7,454	41,214	2.09 (-1.68, 5.85)	0.277
	change in SBP in childhood (mmHg/y): 3 to 10y	7,454	41,214	-0.03 (-0.65, 0.60)	0.935
	change in SBP in adolescence (mmHg/y): 10 to 18y	7,454	41,214	0.04 (-0.41, 0.49)	0.860
	change in SBP in adulthood (mmHg/y): 18 to 26y	7,454	41,214	-0.04 (-0.63, 0.55)	0.906
	change in SBP from childhood to early adulthood				0.998
Average greenness within 500m buffer	mean difference in SBP at age 3y (mmHg)	7,454	41,214	3.03 (-0.79, 6.86)	0.120
	change in SBP in childhood (mmHg/y): 3 to 10y	7,454	41,214	-0.19 (-0.82, 0.45)	0.562
	change in SBP in adolescence (mmHg/y): 10 to 18y	7,454	41,214	-0.04 (-0.50, 0.42)	0.856
	change in SBP in adulthood (mmHg/y): 18 to 26y	7,454	41,214	-0.06 (-0.66, 0.54)	0.845
	change in SBP from childhood to early adulthood				0.889
Food facilities density within 300m buffer	mean difference in SBP at age 3y (mmHg)	7,453	41,213	-0.03 (-0.26, 0.21)	0.829
	change in SBP in childhood (mmHg/y): 3 to 10y	7,453	41,213	-0.02 (-0.06, 0.02)	0.257
	change in SBP in adolescence (mmHg/y): 10 to 18y	7,453	41,213	0.01 (-0.01, 0.04)	0.351
	change in SBP in adulthood (mmHg/y): 18 to 26y	7,453	41,213	0.01 (-0.02, 0.05)	0.505
	change in SBP from childhood to early adulthood				0.451
NO_2	mean difference in SBP at age 3y (mmHg)	7,412	40,984	-0.24 (-0.67, 0.19)	0.271
	change in SBP in childhood (mmHg/y): 3 to 10y	7,412	40,984	0.01 (-0.06, 0.08)	0.793
	change in SBP in adolescence (mmHg/y): 10 to 18y	7,412	40,984	0.00 (-0.05, 0.05)	0.940

	change in SBP in adulthood (mmHg/y): 18 to 26y	7,412	40,984	-0.02 (-0.09, 0.04)	0.468
	change in SBP from childhood to early adulthood				0.888
$PM_{2.5}$	mean difference in SBP at age 3y (mmHg)	7,412	40,984	-0.52 (-0.95, -0.09)	0.017
	change in SBP in childhood (mmHg/y): 3 to 10y	7,412	40,984	0.11 (0.03, 0.18)	0.004
	change in SBP in adolescence (mmHg/y): 10 to 18y	7,412	40,984	-0.02 (-0.07, 0.03)	0.451
	change in SBP in adulthood (mmHg/y): 18 to 26y	7,412	40,984	-0.04 (-0.11, 0.03)	0.264
	change in SBP from childhood to early adulthood				0.018
PM_{10}	mean difference in SBP at age 3y (mmHg)	7,024	38,845	-0.53 (-0.95, -0.11)	0.013
	change in SBP in childhood (mmHg/y): 3 to 10y	7,024	38,845	0.04 (-0.03, 0.11)	0.271
	change in SBP in adolescence (mmHg/y): 10 to 18y	7,024	38,845	0.05 (0.00, 0.11)	0.052
	change in SBP in adulthood (mmHg/y): 18 to 26y	7,024	38,845	-0.01 (-0.09, 0.06)	0.716
	change in SBP from childhood to early adulthood				0.004

Adjusted for maternal education, age at delivery, ethnicity, area deprivation, and sex. Betas correspond to mean change in blood pressure by an interquartile range (IQR) increase in the exposure (for continuous exposures), or by change in category of exposure (for categorical exposures); the IQR and categories for each exposure are presented in Supplemental Table 4.

Supplemental Table 7. Associations between urban environmental exposures and changes in diastolic blood pressure (DBP) in ALSPAC

Exposure	Parameter	N	N obs	beta (95% CI)	p-value
Lden	mean difference in DBP at age 3y (mmHg)	5,095	28,134	-0.08 (-0.49, 0.32)	0.688
	change in DBP in childhood (mmHg/y): 3 to 10y	5,095	28,134	0.04 (-0.03, 0.10)	0.303
	change in DBP in adolescence (mmHg/y): 10 to 18y	5,095	28,134	-0.03 (-0.07, 0.02)	0.208
	change in DBP in adulthood (mmHg/y): 18 to 26y	5,095	28,134	0.00 (-0.07, 0.07)	0.946
	change in DBP from childhood to early adulthood				0.530
Lnight	mean difference in DBP at age 3y (mmHg)	5,095	28,134	-0.27 (-0.83, 0.30)	0.355
	change in DBP in childhood (mmHg/y): 3 to 10y	5,095	28,134	0.07 (-0.02, 0.17)	0.130
	change in DBP in adolescence (mmHg/y): 10 to 18y	5,095	28,134	-0.05 (-0.11, 0.01)	0.112
	change in DBP in adulthood (mmHg/y): 18 to 26y	5,095	28,134	0.03 (-0.06, 0.13)	0.503
	change in DBP from childhood to early adulthood				0.316
Inverse distance to nearest road	mean difference in DBP at age 3y (mmHg)	7,454	41,216	-0.14 (-0.42, 0.14)	0.325
	change in DBP in childhood (mmHg/y): 3 to 10y	7,454	41,216	0.02 (-0.02, 0.07)	0.341
	change in DBP in adolescence (mmHg/y): 10 to 18y	7,454	41,216	0.00 (-0.03, 0.04)	0.780
	change in DBP in adulthood (mmHg/y): 18 to 26y	7,454	41,216	-0.04 (-0.09, 0.01)	0.139
	change in DBP from childhood to early adulthood				0.342
Humidity	mean difference in DBP at age 3y (mmHg)	7,402	40,927	-1.57 (-2.01, -1.12)	5.27E-12
	change in DBP in childhood (mmHg/y): 3 to 10y	7,402	40,927	0.34 (0.26, 0.41)	3.27E-19
	change in DBP in adolescence (mmHg/y): 10 to 18y	7,402	40,927	-0.16 (-0.20, -0.12)	5.29E-13
	change in DBP in adulthood (mmHg/y): 18 to 26y	7,402	40,927	0.06 (-0.01, 0.13)	0.115
	change in DBP from childhood to early adulthood				1.80E-21
Mean temperature	mean difference in DBP at age 3y (mmHg)	7,413	40,990	0.16 (-0.32, 0.65)	0.508
	change in DBP in childhood (mmHg/y): 3 to 10y	7,413	40,990	-0.09 (-0.17, -0.01)	0.033
	change in DBP in adolescence (mmHg/y): 10 to 18y	7,413	40,990	0.08 (0.03, 0.13)	0.002
	change in DBP in adulthood (mmHg/y): 18 to 26y	7,413	40,990	0.03 (-0.04, 0.11)	0.390
	change in DBP from childhood to early adulthood				0.001
Building density in 100m buffer	mean difference in DBP at age 3y (mmHg)	7,447	41,174	0.14 (-0.19, 0.48)	0.408
	change in DBP in childhood (mmHg/y): 3 to 10y	7,447	41,174	-0.03 (-0.08, 0.03)	0.363
	change in DBP in adolescence (mmHg/y): 10 to 18y	7,447	41,174	0.01 (-0.03, 0.04)	0.638

	change in DBP in adulthood (mmHg/y): 18 to 26y	7,447	41,174	-0.03 (-0.09, 0.02)	0.249
	change in DBP from childhood to early adulthood				0.543
Building density in 300m buffer	mean difference in DBP at age 3y (mmHg)	7,450	41,193	0.09 (-0.25, 0.42)	0.606
	change in DBP in childhood (mmHg/y): 3 to 10y	7,450	41,193	-0.02 (-0.08, 0.03)	0.460
	change in DBP in adolescence (mmHg/y): 10 to 18y	7,450	41,193	0.01 (-0.02, 0.05)	0.432
	change in DBP in adulthood (mmHg/y): 18 to 26y	7,450	41,193	-0.03 (-0.08, 0.02)	0.269
	change in DBP from childhood to early adulthood				0.645
Connectivity in 100m buffer	mean difference in DBP at age 3y (mmHg)	7,075	38,941	-0.12 (-0.40, 0.17)	0.423
	change in DBP in childhood (mmHg/y): 3 to 10y	7,075	38,941	0.01 (-0.04, 0.05)	0.817
	change in DBP in adolescence (mmHg/y): 10 to 18y	7,075	38,941	-0.02 (-0.05, 0.01)	0.231
	change in DBP in adulthood (mmHg/y): 18 to 26y	7,075	38,941	-0.02 (-0.07, 0.02)	0.358
	change in DBP from childhood to early adulthood				0.250
Connectivity in 300m buffer	mean difference in DBP at age 3y (mmHg)	7,432	41,073	0.03 (-0.30, 0.35)	0.867
	change in DBP in childhood (mmHg/y): 3 to 10y	7,432	41,073	-0.01 (-0.06, 0.04)	0.742
	change in DBP in adolescence (mmHg/y): 10 to 18y	7,432	41,073	0.00 (-0.03, 0.03)	0.928
	change in DBP in adulthood (mmHg/y): 18 to 26y	7,432	41,073	-0.02 (-0.07, 0.03)	0.381
	change in DBP from childhood to early adulthood				0.791
Facility density within 300m buffer	mean difference in DBP at age 3y (mmHg)	7,453	41,215	0.10 (-0.09, 0.30)	0.307
	change in DBP in childhood (mmHg/y): 3 to 10y	7,453	41,215	-0.02 (-0.06, 0.01)	0.140
	change in DBP in adolescence (mmHg/y): 10 to 18y	7,453	41,215	0.01 (-0.01, 0.03)	0.319
	change in DBP in adulthood (mmHg/y): 18 to 26y	7,453	41,215	-0.02 (-0.05, 0.01)	0.223
	change in DBP from childhood to early adulthood				0.331
Land use within 300m buffer	mean difference in DBP at age 3y (mmHg)	7,246	39,977	-0.04 (-0.40, 0.33)	0.840
	change in DBP in childhood (mmHg/y): 3 to 10y	7,246	39,977	0.00 (-0.06, 0.06)	0.916
	change in DBP in adolescence (mmHg/y): 10 to 18y	7,246	39,977	0.02 (-0.02, 0.06)	0.396
	change in DBP in adulthood (mmHg/y): 18 to 26y	7,246	39,977	-0.02 (-0.09, 0.04)	0.431
	change in DBP from childhood to early adulthood				0.779
Agricultural area within 300m buffer	mean difference in DBP at age 3y (mmHg)	7,246	39,977	-0.01 (-0.09, 0.07)	0.759
	change in DBP in childhood (mmHg/y): 3 to 10y	7,246	39,977	0.00 (-0.02, 0.01)	0.724
	change in DBP in adolescence (mmHg/y): 10 to 18y	7,246	39,977	0.00 (-0.01, 0.00)	0.419
	change in DBP in adulthood (mmHg/y): 18 to 26y	7,246	39,977	0.01 (0.00, 0.02)	0.219

	change in DBP from childhood to early adulthood				0.579
Airport within 300m buffer	mean difference in DBP at age 3y (mmHg)	7,246	39,977	-0.73 (-4.30, 2.84)	0.688
	change in DBP in childhood (mmHg/y): 3 to 10y	7,246	39,977	0.32 (-0.27, 0.91)	0.285
	change in DBP in adolescence (mmHg/y): 10 to 18y	7,246	39,977	-0.08 (-0.45, 0.28)	0.665
	change in DBP in adulthood (mmHg/y): 18 to 26y	7,246	39,977	0.47 (-0.04, 0.99)	0.071
	change in DBP from childhood to early adulthood				0.186
Continuous Urban Fabric within 300m buffer	mean difference in DBP at age 3y (mmHg)	7,246	39,977	0.01 (-0.29, 0.30)	0.969
	change in DBP in childhood (mmHg/y): 3 to 10y	7,246	39,977	-0.01 (-0.06, 0.04)	0.605
	change in DBP in adolescence (mmHg/y): 10 to 18y	7,246	39,977	0.02 (-0.01, 0.05)	0.169
	change in DBP in adulthood (mmHg/y): 18 to 26y	7,246	39,977	-0.05 (-0.10, 0.00)	0.039
	change in DBP from childhood to early adulthood				0.205
Industrial, commercial, public, military and private units within 300m buffer	mean difference in DBP at age 3y (mmHg)	7,246	39,977	-0.20 (-0.48, 0.08)	0.166
	change in DBP in childhood (mmHg/y): 3 to 10y	7,246	39,977	0.04 (-0.01, 0.09)	0.089
	change in DBP in adolescence (mmHg/y): 10 to 18y	7,246	39,977	-0.02 (-0.05, 0.01)	0.114
	change in DBP in adulthood (mmHg/y): 18 to 26y	7,246	39,977	0.01 (-0.04, 0.06)	0.606
	change in DBP from childhood to early adulthood				0.264
Discontinuous Low Density Urban Fabric within 300m buffer	mean difference in DBP at age 3y (mmHg)	7,246	39,977	0.15 (-0.23, 0.52)	0.444
	change in DBP in childhood (mmHg/y): 3 to 10y	7,246	39,977	-0.02 (-0.08, 0.04)	0.577
	change in DBP in adolescence (mmHg/y): 10 to 18y	7,246	39,977	0.02 (-0.02, 0.06)	0.387
	change in DBP in adulthood (mmHg/y): 18 to 26y	7,246	39,977	-0.01 (-0.07, 0.05)	0.772
	change in DBP from childhood to early adulthood				0.843
Forests within 300m buffer	mean difference in DBP at age 3y (mmHg)	7,246	39,977	0.08 (-0.36, 0.52)	0.731
	change in DBP in childhood (mmHg/y): 3 to 10y	7,246	39,977	-0.03 (-0.10, 0.05)	0.481
	change in DBP in adolescence (mmHg/y): 10 to 18y	7,246	39,977	0.00 (-0.05, 0.05)	0.901
	change in DBP in adulthood (mmHg/y): 18 to 26y	7,246	39,977	0.01 (-0.07, 0.08)	0.898
	change in DBP from childhood to early adulthood				0.911
Mineral extraction and dump sites, constructions within 300m buffer	mean difference in DBP at age 3y (mmHg)	7,246	39,977	-0.26 (-0.75, 0.22)	0.288
	change in DBP in childhood (mmHg/y): 3 to 10y	7,246	39,977	0.05 (-0.03, 0.13)	0.224
	change in DBP in adolescence (mmHg/y): 10 to 18y	7,246	39,977	-0.06 (-0.12, -0.01)	0.022
	change in DBP in adulthood (mmHg/y): 18 to 26y	7,246	39,977	0.08 (-0.01, 0.16)	0.087
	change in DBP from childhood to early adulthood				0.103

Port areas within 300m buffer	mean difference in DBP at age 3y (mmHg)	7,246	39,977	0.85 (-4.91, 6.62)	0.772
	change in DBP in childhood (mmHg/y): 3 to 10y	7,246	39,977	0.06 (-0.90, 1.01)	0.908
	change in DBP in adolescence (mmHg/y): 10 to 18y	7,246	39,977	-0.48 (-1.05, 0.09)	0.099
	change in DBP in adulthood (mmHg/y): 18 to 26y	7,246	39,977	0.27 (-0.84, 1.38)	0.636
	change in DBP from childhood to early adulthood				0.374
Transport networks and other constructed hard-surfaced areas within 300m buffer	mean difference in DBP at age 3y (mmHg)	7,246	39,977	0.07 (-0.25, 0.39)	0.665
	change in DBP in childhood (mmHg/y): 3 to 10y	7,246	39,977	-0.01 (-0.07, 0.04)	0.608
	change in DBP in adolescence (mmHg/y): 10 to 18y	7,246	39,977	0.01 (-0.03, 0.04)	0.698
	change in DBP in adulthood (mmHg/y): 18 to 26y	7,246	39,977	-0.02 (-0.07, 0.03)	0.479
	change in DBP from childhood to early adulthood				0.869
Green urban areas within 300m buffer	mean difference in DBP at age 3y (mmHg)	7,246	39,977	-0.04 (-0.36, 0.28)	0.817
	change in DBP in childhood (mmHg/y): 3 to 10y	7,246	39,977	0.03 (-0.03, 0.08)	0.342
	change in DBP in adolescence (mmHg/y): 10 to 18y	7,246	39,977	0.02 (-0.01, 0.06)	0.239
	change in DBP in adulthood (mmHg/y): 18 to 26y	7,246	39,977	-0.04 (-0.09, 0.02)	0.180
	change in DBP from childhood to early adulthood				0.243
Discontinuous very low density urban fabric within 300m buffer	mean difference in DBP at age 3y (mmHg)	7,246	39,977	-0.68 (-1.89, 0.53)	0.268
	change in DBP in childhood (mmHg/y): 3 to 10y	7,246	39,977	0.06 (-0.14, 0.26)	0.528
	change in DBP in adolescence (mmHg/y): 10 to 18y	7,246	39,977	0.04 (-0.08, 0.16)	0.528
	change in DBP in adulthood (mmHg/y): 18 to 26y	7,246	39,977	-0.18 (-0.36, 0.01)	0.058
	change in DBP from childhood to early adulthood				0.236
Population density	mean difference in DBP at age 3y (mmHg)	7,308	40,349	0.24 (-0.11, 0.59)	0.186
	change in DBP in childhood (mmHg/y): 3 to 10y	7,308	40,349	-0.07 (-0.13, -0.01)	0.025
	change in DBP in adolescence (mmHg/y): 10 to 18y	7,308	40,349	0.02 (-0.02, 0.06)	0.313
	change in DBP in adulthood (mmHg/y): 18 to 26y	7,308	40,349	0.02 (-0.04, 0.08)	0.460
	change in DBP from childhood to early adulthood				0.095
Bus lines in 300m buffer	mean difference in DBP at age 3y (mmHg)	6,800	37,495	0.06 (-0.29, 0.41)	0.755
	change in DBP in childhood (mmHg/y): 3 to 10y	6,800	37,495	0.01 (-0.05, 0.07)	0.703
	change in DBP in adolescence (mmHg/y): 10 to 18y	6,800	37,495	-0.03 (-0.06, 0.01)	0.188
	change in DBP in adulthood (mmHg/y): 18 to 26y	6,800	37,495	0.02 (-0.04, 0.08)	0.505
	change in DBP from childhood to early adulthood				0.625
Bus lines in 500m buffer	mean difference in DBP at age 3y (mmHg)	7,226	39,899	0.03 (-0.34, 0.39)	0.892

	change in DBP in childhood (mmHg/y): 3 to 10y	7,226	39,899	-0.01 (-0.07, 0.05)	0.855
	change in DBP in adolescence (mmHg/y): 10 to 18y	7,226	39,899	-0.02 (-0.06, 0.02)	0.249
	change in DBP in adulthood (mmHg/y): 18 to 26y	7,226	39,899	0.01 (-0.05, 0.08)	0.671
	change in DBP from childhood to early adulthood				0.623
Walkability index	mean difference in DBP at age 3y (mmHg)	7,224	39,834	0.08 (-0.24, 0.41)	0.620
	change in DBP in childhood (mmHg/y): 3 to 10y	7,224	39,834	-0.03 (-0.08, 0.03)	0.334
	change in DBP in adolescence (mmHg/y): 10 to 18y	7,224	39,834	0.01 (-0.02, 0.04)	0.579
	change in DBP in adulthood (mmHg/y): 18 to 26y	7,224	39,834	-0.03 (-0.08, 0.02)	0.291
	change in DBP from childhood to early adulthood				0.582
Distance to closest blue space	mean difference in DBP at age 3y (mmHg)	7,243	39,954	0.21 (-0.13, 0.54)	0.225
	change in DBP in childhood (mmHg/y): 3 to 10y	7,243	39,954	-0.03 (-0.09, 0.02)	0.261
	change in DBP in adolescence (mmHg/y): 10 to 18y	7,243	39,954	-0.01 (-0.05, 0.02)	0.500
	change in DBP in adulthood (mmHg/y): 18 to 26y	7,243	39,954	0.05 (-0.01, 0.10)	0.128
	change in DBP from childhood to early adulthood				0.247
Size of blue space	mean difference in DBP at age 3y (mmHg)	7,240	39,939	-0.09 (-0.28, 0.11)	0.375
	change in DBP in childhood (mmHg/y): 3 to 10y	7,240	39,939	0.01 (-0.02, 0.04)	0.469
	change in DBP in adolescence (mmHg/y): 10 to 18y	7,240	39,939	0.00 (-0.02, 0.02)	0.840
	change in DBP in adulthood (mmHg/y): 18 to 26y	7,240	39,939	-0.01 (-0.04, 0.02)	0.569
	change in DBP from childhood to early adulthood				0.807
Existence blue space within 300m buffer	mean difference in DBP at age 3y (mmHg)	7,243	39,954	0.46 (-0.63, 1.56)	0.407
	change in DBP in childhood (mmHg/y): 3 to 10y	7,243	39,954	-0.07 (-0.25, 0.11)	0.446
	change in DBP in adolescence (mmHg/y): 10 to 18y	7,243	39,954	0.01 (-0.11, 0.13)	0.847
	change in DBP in adulthood (mmHg/y): 18 to 26y	7,243	39,954	-0.08 (-0.26, 0.09)	0.361
	change in DBP from childhood to early adulthood				0.662
Distance to closest green space	mean difference in DBP at age 3y (mmHg)	7,243	39,954	0.03 (-0.34, 0.39)	0.888
	change in DBP in childhood (mmHg/y): 3 to 10y	7,243	39,954	0.00 (-0.06, 0.06)	0.965
	change in DBP in adolescence (mmHg/y): 10 to 18y	7,243	39,954	-0.02 (-0.06, 0.02)	0.428
	change in DBP in adulthood (mmHg/y): 18 to 26y	7,243	39,954	0.00 (-0.06, 0.06)	0.970
	change in DBP from childhood to early adulthood				0.807
Size of green space	mean difference in DBP at age 3y (mmHg)	7,243	39,954	0.09 (-0.27, 0.45)	0.631
	change in DBP in childhood (mmHg/y): 3 to 10y	7,243	39,954	0.03 (-0.03, 0.09)	0.408

	change in DBP in adolescence (mmHg/y): 10 to 18y	7,243	39,954	-0.04 (-0.07, 0.00)	0.064
	change in DBP in adulthood (mmHg/y): 18 to 26y	7,243	39,954	0.04 (-0.02, 0.10)	0.247
	change in DBP from childhood to early adulthood				0.305
Existence green space within 300m buffer	mean difference in DBP at age 3y (mmHg)	7,243	39,954	0.08 (-0.54, 0.69)	0.801
	change in DBP in childhood (mmHg/y): 3 to 10y	7,243	39,954	-0.01 (-0.11, 0.09)	0.860
	change in DBP in adolescence (mmHg/y): 10 to 18y	7,243	39,954	0.03 (-0.04, 0.09)	0.403
	change in DBP in adulthood (mmHg/y): 18 to 26y	7,243	39,954	0.02 (-0.08, 0.13)	0.652
	change in DBP from childhood to early adulthood				0.665
Average greenness within 100m buffer	mean difference in DBP at age 3y (mmHg)	7,454	41,216	0.56 (-2.23, 3.35)	0.694
	change in DBP in childhood (mmHg/y): 3 to 10y	7,454	41,216	-0.24 (-0.70, 0.22)	0.313
	change in DBP in adolescence (mmHg/y): 10 to 18y	7,454	41,216	0.18 (-0.11, 0.48)	0.218
	change in DBP in adulthood (mmHg/y): 18 to 26y	7,454	41,216	0.09 (-0.37, 0.54)	0.709
	change in DBP from childhood to early adulthood				0.420
Average greenness within 300m buffer	mean difference in DBP at age 3y (mmHg)	7,454	41,216	0.30 (-2.64, 3.24)	0.841
	change in DBP in childhood (mmHg/y): 3 to 10y	7,454	41,216	-0.10 (-0.58, 0.38)	0.676
	change in DBP in adolescence (mmHg/y): 10 to 18y	7,454	41,216	0.13 (-0.17, 0.43)	0.398
	change in DBP in adulthood (mmHg/y): 18 to 26y	7,454	41,216	0.10 (-0.37, 0.58)	0.671
	change in DBP from childhood to early adulthood				0.686
Average greenness within 500m buffer	mean difference in DBP at age 3y (mmHg)	7,454	41,216	-0.42 (-3.41, 2.56)	0.781
	change in DBP in childhood (mmHg/y): 3 to 10y	7,454	41,216	0.06 (-0.43, 0.55)	0.812
	change in DBP in adolescence (mmHg/y): 10 to 18y	7,454	41,216	-0.04 (-0.35, 0.27)	0.797
	change in DBP in adulthood (mmHg/y): 18 to 26y	7,454	41,216	0.19 (-0.29, 0.67)	0.442
	change in DBP from childhood to early adulthood				0.885
Food facilities density within 300m buffer	mean difference in DBP at age 3y (mmHg)	7,453	41,215	0.17 (-0.02, 0.35)	0.074
	change in DBP in childhood (mmHg/y): 3 to 10y	7,453	41,215	-0.04 (-0.07, -0.01)	0.021
	change in DBP in adolescence (mmHg/y): 10 to 18y	7,453	41,215	0.01 (-0.01, 0.03)	0.373
	change in DBP in adulthood (mmHg/y): 18 to 26y	7,453	41,215	-0.01 (-0.03, 0.02)	0.726
	change in DBP from childhood to early adulthood				0.148
NO_2	mean difference in DBP at age 3y (mmHg)	7,412	40,986	-0.14 (-0.48, 0.19)	0.402
	change in DBP in childhood (mmHg/y): 3 to 10y	7,412	40,986	0.03 (-0.03, 0.08)	0.368
	change in DBP in adolescence (mmHg/y): 10 to 18y	7,412	40,986	0.00 (-0.03, 0.04)	0.882

	change in DBP in adulthood (mmHg/y): 18 to 26y	7,412	40,986	-0.05 (-0.10, 0.00)	0.060
	change in DBP from childhood to early adulthood				0.187
$PM_{2.5}$	mean difference in DBP at age 3y (mmHg)	7,412	40,986	-0.41 (-0.75, -0.07)	0.017
	change in DBP in childhood (mmHg/y): 3 to 10y	7,412	40,986	0.11 (0.06, 0.17)	8.85E-05
	change in DBP in adolescence (mmHg/y): 10 to 18y	7,412	40,986	-0.07 (-0.10, -0.03)	3.27E-04
	change in DBP in adulthood (mmHg/y): 18 to 26y	7,412	40,986	0.00 (-0.05, 0.06)	0.982
	change in DBP from childhood to early adulthood				3.95E-05
PM_{10}	mean difference in DBP at age 3y (mmHg)	7,024	38,846	-0.35 (-0.67, -0.02)	0.036
	change in DBP in childhood (mmHg/y): 3 to 10y	7,024	38,846	0.04 (-0.01, 0.10)	0.125
	change in DBP in adolescence (mmHg/y): 10 to 18y	7,024	38,846	0.01 (-0.03, 0.04)	0.742
	change in DBP in adulthood (mmHg/y): 18 to 26y	7,024	38,846	-0.07 (-0.13, -0.01)	0.020
	change in DBP from childhood to early adulthood				0.038

Adjusted for maternal education, age at delivery, ethnicity, area deprivation, and sex. Betas correspond to mean change in blood pressure by an interquartile range (IQR) increase in the exposure (for continuous exposures), or by change in category of exposure (for categorical exposures); the IQR and categories for each exposure are presented in Supplemental Table 4.

Supplemental Table 8. Average predicted systolic and diastolic blood pressure according to different levels of urban environmental exposures in ALSPAC

Exposure	Percentile/ average level of exposure	Age (years)	Predicted blood pressure (mmHg) Mean (95%CI)
**Systolic blood pressure**			
Humidity	25th (81.1%)	3	91.9 (91.1, 92.7)
		10	101.8 (101.1, 102.5)
		18	122.1 (121.3, 122.8)
		24	116.3 (115.5, 117.1)
	75th (83.1%)	3	89.9 (89.1, 90.7)
		10	101.8 (101.1, 102.5)
		18	122.6 (121.9, 123.3)
		24	116.1 (115.3, 116.9)
Temperature	25th (8.3 °C)	3	90.5 (89.7, 91.3)
		10	101.9 (101.2, 102.6)
		18	122.4 (121.7, 123.2)
		24	116.2 (115.5, 117.0)
	75th (10.7 °C)	3	91.4 (90.6, 92.2)
		10	101.6 (100.9, 102.3)
		18	122.2 (121.5, 123.0)
		24	116.2 (115.4, 117.0)
**Diastolic blood pressure**			
Humidity	25th (81.1%)	3	55.3 (54.8, 55.9)
		10	55.4 (54.9, 55.9)
		18	64.5 (64.0, 65.0)
		24	65.2 (64.6, 65.7)
	75th (83.1%)	3	53.8 (53.2, 54.3)
		10	56.2 (55.7, 56.7)
		18	64.0 (63.5, 64.5)
		24	65.0 (64.4, 65.6)
Temperature	25th (8.3 °C)	3	54.5 (54.0, 55.1)
		10	56.0 (55.5, 56.5)
		18	64.2 (63.6, 64.7)
		24	64.9 (64.3, 65.5)
	75th (10.7 °C)	3	54.7 (54.1, 55.3)
		10	55.6 (55.1, 56.1)
		18	64.3 (63.8, 64.9)
		24	65.3 (64.7, 65.9)

Adjusted for maternal education, age at delivery, ethnicity, area deprivation, and sex. All covariates used in the adjustment were set to the mean value or the reference category: maternal education (high), age at delivery (28.9 years), ethnicity (White), area deprivation (least deprived), and sex (male).

Supplemental Table 9. Associations between urban environmental exposures and changes in systolic blood pressure (SBP) in ALSPAC, restricted to those with 3 or more blood pressure measurements

Exposure	Parameter	N	N obs	beta (95% CI)	p-value
Lden	mean difference in SBP at age 3y (mmHg)	4,199	26,873	0.14 (-0.41, 0.68)	0.622
	change in SBP in childhood (mmHg/y): 3 to 10y	4,199	26,873	-0.01 (-0.10, 0.08)	0.832
	change in SBP in adolescence (mmHg/y): 10 to 18y	4,199	26,873	0.02 (-0.04, 0.09)	0.518
	change in SBP in adulthood (mmHg/y): 18 to 26y	4,199	26,873	-0.05 (-0.14, 0.04)	0.299
	change in SBP from childhood to early adulthood				0.762
Lnight	mean difference in SBP at age 3y (mmHg)	4,199	26,873	-0.18 (-0.94, 0.58)	0.642
	change in SBP in childhood (mmHg/y): 3 to 10y	4,199	26,873	0.04 (-0.09, 0.16)	0.549
	change in SBP in adolescence (mmHg/y): 10 to 18y	4,199	26,873	0.05 (-0.04, 0.14)	0.292
	change in SBP in adulthood (mmHg/y): 18 to 26y	4,199	26,873	-0.06 (-0.18, 0.07)	0.373
	change in SBP from childhood to early adulthood				0.476
Inverse distance to nearest road	mean difference in SBP at age 3y (mmHg)	6,129	39,356	-0.21 (-0.58, 0.16)	0.265
	change in SBP in childhood (mmHg/y): 3 to 10y	6,129	39,356	0.02 (-0.04, 0.08)	0.585
	change in SBP in adolescence (mmHg/y): 10 to 18y	6,129	39,356	0.00 (-0.04, 0.05)	0.899
	change in SBP in adulthood (mmHg/y): 18 to 26y	6,129	39,356	-0.04 (-0.10, 0.02)	0.217
	change in SBP from childhood to early adulthood				0.585
Humidity	mean difference in SBP at age 3y (mmHg)	6,087	39,081	-1.97 (-2.55, -1.39)	2.19E-11
	change in SBP in childhood (mmHg/y): 3 to 10y	6,087	39,081	0.29 (0.20, 0.39)	2.01E-09
	change in SBP in adolescence (mmHg/y): 10 to 18y	6,087	39,081	0.05 (-0.01, 0.12)	0.100
	change in SBP in adulthood (mmHg/y): 18 to 26y	6,087	39,081	-0.09 (-0.18, -0.01)	0.034
	change in SBP from childhood to early adulthood				5.06E-12
Mean temperature	mean difference in SBP at age 3y (mmHg)	6,096	39,141	0.93 (0.30, 1.56)	0.004
	change in SBP in childhood (mmHg/y): 3 to 10y	6,096	39,141	-0.19 (-0.30, -0.09)	3.34E-04
	change in SBP in adolescence (mmHg/y): 10 to 18y	6,096	39,141	0.04 (-0.04, 0.11)	0.364
	change in SBP in adulthood (mmHg/y): 18 to 26y	6,096	39,141	0.01 (-0.09, 0.11)	0.889
	change in SBP from childhood to early adulthood				0.004
Building density in 100m buffer	mean difference in SBP at age 3y (mmHg)	6,123	39,316	-0.09 (-0.53, 0.35)	0.701
	change in SBP in childhood (mmHg/y): 3 to 10y	6,123	39,316	-0.05 (-0.12, 0.03)	0.221

	change in SBP in adolescence (mmHg/y): 10 to 18y	6,123	39,316	0.02 (-0.04, 0.07)	0.537
	change in SBP in adulthood (mmHg/y): 18 to 26y	6,123	39,316	0.01 (-0.06, 0.08)	0.716
	change in SBP from childhood to early adulthood				0.608
Building density in 300m buffer	mean difference in SBP at age 3y (mmHg)	6,126	39,335	-0.17 (-0.60, 0.27)	0.457
	change in SBP in childhood (mmHg/y): 3 to 10y	6,126	39,335	-0.02 (-0.09, 0.05)	0.549
	change in SBP in adolescence (mmHg/y): 10 to 18y	6,126	39,335	0.01 (-0.04, 0.06)	0.707
	change in SBP in adulthood (mmHg/y): 18 to 26y	6,126	39,335	0.01 (-0.05, 0.08)	0.679
	change in SBP from childhood to early adulthood				0.875
Connectivity in 100m buffer	mean difference in SBP at age 3y (mmHg)	5,801	37,155	-0.15 (-0.53, 0.22)	0.424
	change in SBP in childhood (mmHg/y): 3 to 10y	5,801	37,155	-0.01 (-0.07, 0.05)	0.811
	change in SBP in adolescence (mmHg/y): 10 to 18y	5,801	37,155	-0.01 (-0.06, 0.03)	0.643
	change in SBP in adulthood (mmHg/y): 18 to 26y	5,801	37,155	0.03 (-0.02, 0.09)	0.260
	change in SBP from childhood to early adulthood				0.701
Connectivity in 300m buffer	mean difference in SBP at age 3y (mmHg)	6,108	39,215	0.01 (-0.42, 0.44)	0.962
	change in SBP in childhood (mmHg/y): 3 to 10y	6,108	39,215	-0.04 (-0.11, 0.03)	0.221
	change in SBP in adolescence (mmHg/y): 10 to 18y	6,108	39,215	0.02 (-0.03, 0.07)	0.396
	change in SBP in adulthood (mmHg/y): 18 to 26y	6,108	39,215	0.01 (-0.05, 0.08)	0.742
	change in SBP from childhood to early adulthood				0.563
Facility density within 300m buffer	mean difference in SBP at age 3y (mmHg)	6,129	39,356	0.00 (-0.26, 0.25)	0.988
	change in SBP in childhood (mmHg/y): 3 to 10y	6,129	39,356	-0.03 (-0.07, 0.01)	0.204
	change in SBP in adolescence (mmHg/y): 10 to 18y	6,129	39,356	0.01 (-0.02, 0.04)	0.507
	change in SBP in adulthood (mmHg/y): 18 to 26y	6,129	39,356	-0.01 (-0.05, 0.03)	0.614
	change in SBP from childhood to early adulthood				0.617
Land use within 300m buffer	mean difference in SBP at age 3y (mmHg)	5,949	38,155	0.19 (-0.29, 0.68)	0.433
	change in SBP in childhood (mmHg/y): 3 to 10y	5,949	38,155	-0.01 (-0.09, 0.07)	0.767
	change in SBP in adolescence (mmHg/y): 10 to 18y	5,949	38,155	-0.03 (-0.08, 0.03)	0.401
	change in SBP in adulthood (mmHg/y): 18 to 26y	5,949	38,155	-0.05 (-0.12, 0.03)	0.250
	change in SBP from childhood to early adulthood				0.298
Agricultural area within 300m buffer	mean difference in SBP at age 3y (mmHg)	5,949	38,155	0.00 (-0.11, 0.10)	0.941
	change in SBP in childhood (mmHg/y): 3 to 10y	5,949	38,155	0.00 (-0.02, 0.02)	0.892
	change in SBP in adolescence (mmHg/y): 10 to 18y	5,949	38,155	0.00 (-0.01, 0.01)	0.790

	change in SBP in adulthood (mmHg/y): 18 to 26y	5,949	38,155	0.00 (-0.02, 0.01)	0.881
	change in SBP from childhood to early adulthood				0.995
Airport within 300m buffer	mean difference in SBP at age 3y (mmHg)	5,949	38,155	-2.38 (-6.89, 2.13)	0.301
	change in SBP in childhood (mmHg/y): 3 to 10y	5,949	38,155	0.49 (-0.26, 1.24)	0.199
	change in SBP in adolescence (mmHg/y): 10 to 18y	5,949	38,155	0.00 (-0.53, 0.54)	0.990
	change in SBP in adulthood (mmHg/y): 18 to 26y	5,949	38,155	0.15 (-0.49, 0.78)	0.647
	change in SBP from childhood to early adulthood				0.513
Continuous Urban Fabric within 300m buffer	mean difference in SBP at age 3y (mmHg)	5,949	38,155	-0.36 (-0.74, 0.03)	0.072
	change in SBP in childhood (mmHg/y): 3 to 10y	5,949	38,155	0.02 (-0.04, 0.08)	0.565
	change in SBP in adolescence (mmHg/y): 10 to 18y	5,949	38,155	0.00 (-0.04, 0.05)	0.888
	change in SBP in adulthood (mmHg/y): 18 to 26y	5,949	38,155	-0.01 (-0.07, 0.06)	0.769
	change in SBP from childhood to early adulthood				0.914
Industrial, commercial, public, military and private units within 300m buffer	mean difference in SBP at age 3y (mmHg)	5,949	38,155	-0.02 (-0.39, 0.35)	0.904
	change in SBP in childhood (mmHg/y): 3 to 10y	5,949	38,155	0.03 (-0.04, 0.09)	0.418
	change in SBP in adolescence (mmHg/y): 10 to 18y	5,949	38,155	-0.03 (-0.08, 0.01)	0.144
	change in SBP in adulthood (mmHg/y): 18 to 26y	5,949	38,155	-0.01 (-0.07, 0.05)	0.692
	change in SBP from childhood to early adulthood				0.366
Discontinuous low density urban fabric within 300m buffer	mean difference in SBP at age 3y (mmHg)	5,949	38,155	-0.21 (-0.71, 0.28)	0.398
	change in SBP in childhood (mmHg/y): 3 to 10y	5,949	38,155	0.01 (-0.07, 0.09)	0.797
	change in SBP in adolescence (mmHg/y): 10 to 18y	5,949	38,155	0.01 (-0.05, 0.07)	0.716
	change in SBP in adulthood (mmHg/y): 18 to 26y	5,949	38,155	0.02 (-0.06, 0.09)	0.701
	change in SBP from childhood to early adulthood				0.885
Forests within 300m buffer	mean difference in SBP at age 3y (mmHg)	5,949	38,155	-0.29 (-0.88, 0.30)	0.336
	change in SBP in childhood (mmHg/y): 3 to 10y	5,949	38,155	0.01 (-0.08, 0.11)	0.774
	change in SBP in adolescence (mmHg/y): 10 to 18y	5,949	38,155	0.03 (-0.05, 0.10)	0.492
	change in SBP in adulthood (mmHg/y): 18 to 26y	5,949	38,155	-0.01 (-0.11, 0.08)	0.767
	change in SBP from childhood to early adulthood				0.845
Mineral extraction and dump sites, constructions within 300m buffer	mean difference in SBP at age 3y (mmHg)	5,949	38,155	-0.47 (-1.11, 0.18)	0.155
	change in SBP in childhood (mmHg/y): 3 to 10y	5,949	38,155	0.15 (0.04, 0.25)	0.008
	change in SBP in adolescence (mmHg/y): 10 to 18y	5,949	38,155	-0.04 (-0.12, 0.04)	0.369
	change in SBP in adulthood (mmHg/y): 18 to 26y	5,949	38,155	0.04 (-0.07, 0.15)	0.429

	change in SBP from childhood to early adulthood				0.054
Port areas within 300m buffer	mean difference in SBP at age 3y (mmHg)	5,949	38,155	7.15 (-0.66, 14.96)	0.073
	change in SBP in childhood (mmHg/y): 3 to 10y	5,949	38,155	-0.90 (-2.18, 0.39)	0.171
	change in SBP in adolescence (mmHg/y): 10 to 18y	5,949	38,155	0.23 (-0.61, 1.08)	0.590
	change in SBP in adulthood (mmHg/y): 18 to 26y	5,949	38,155	-0.25 (-1.71, 1.22)	0.740
	change in SBP from childhood to early adulthood				0.590
Transport networks and other constructed hard-surfaced areas within 300m buffer	mean difference in SBP at age 3y (mmHg)	5,949	38,155	-0.08 (-0.51, 0.34)	0.696
	change in SBP in childhood (mmHg/y): 3 to 10y	5,949	38,155	-0.01 (-0.08, 0.06)	0.755
	change in SBP in adolescence (mmHg/y): 10 to 18y	5,949	38,155	0.01 (-0.04, 0.06)	0.637
	change in SBP in adulthood (mmHg/y): 18 to 26y	5,949	38,155	-0.02 (-0.09, 0.05)	0.566
	change in SBP from childhood to early adulthood				0.931
Green urban areas within 300m buffer	mean difference in SBP at age 3y (mmHg)	5,949	38,155	0.42 (0.00, 0.84)	0.050
	change in SBP in childhood (mmHg/y): 3 to 10y	5,949	38,155	-0.02 (-0.09, 0.05)	0.509
	change in SBP in adolescence (mmHg/y): 10 to 18y	5,949	38,155	-0.02 (-0.08, 0.03)	0.370
	change in SBP in adulthood (mmHg/y): 18 to 26y	5,949	38,155	0.00 (-0.07, 0.08)	0.897
	change in SBP from childhood to early adulthood				0.567
Discontinuous very low density urban fabric within 300m buffer	mean difference in SBP at age 3y (mmHg)	5,949	38,155	-0.59 (-2.16, 0.97)	0.458
	change in SBP in childhood (mmHg/y): 3 to 10y	5,949	38,155	0.05 (-0.21, 0.31)	0.689
	change in SBP in adolescence (mmHg/y): 10 to 18y	5,949	38,155	0.13 (-0.05, 0.30)	0.150
	change in SBP in adulthood (mmHg/y): 18 to 26y	5,949	38,155	-0.13 (-0.36, 0.10)	0.274
	change in SBP from childhood to early adulthood				0.334
Population density	mean difference in SBP at age 3y (mmHg)	6,002	38,515	-0.02 (-0.49, 0.45)	0.927
	change in SBP in childhood (mmHg/y): 3 to 10y	6,002	38,515	-0.06 (-0.14, 0.02)	0.135
	change in SBP in adolescence (mmHg/y): 10 to 18y	6,002	38,515	0.02 (-0.04, 0.07)	0.584
	change in SBP in adulthood (mmHg/y): 18 to 26y	6,002	38,515	0.07 (0.00, 0.14)	0.067
	change in SBP from childhood to early adulthood				0.088
Bus lines in 300m buffer	mean difference in SBP at age 3y (mmHg)	5,590	35,800	0.46 (-0.01, 0.93)	0.053
	change in SBP in childhood (mmHg/y): 3 to 10y	5,590	35,800	-0.05 (-0.13, 0.03)	0.199
	change in SBP in adolescence (mmHg/y): 10 to 18y	5,590	35,800	-0.03 (-0.08, 0.03)	0.372
	change in SBP in adulthood (mmHg/y): 18 to 26y	5,590	35,800	-0.02 (-0.10, 0.05)	0.529
	change in SBP from childhood to early adulthood				0.165

Bus lines in 500m buffer	mean difference in SBP at age 3y (mmHg)	5,936	38,093	0.26 (-0.22, 0.75)	0.284
	change in SBP in childhood (mmHg/y): 3 to 10y	5,936	38,093	-0.05 (-0.13, 0.03)	0.193
	change in SBP in adolescence (mmHg/y): 10 to 18y	5,936	38,093	-0.01 (-0.07, 0.05)	0.829
	change in SBP in adulthood (mmHg/y): 18 to 26y	5,936	38,093	-0.02 (-0.10, 0.06)	0.663
	change in SBP from childhood to early adulthood				0.444
Walkability index	mean difference in SBP at age 3y (mmHg)	5,928	38,014	0.04 (-0.39, 0.47)	0.858
	change in SBP in childhood (mmHg/y): 3 to 10y	5,928	38,014	-0.04 (-0.11, 0.03)	0.279
	change in SBP in adolescence (mmHg/y): 10 to 18y	5,928	38,014	0.00 (-0.05, 0.05)	0.949
	change in SBP in adulthood (mmHg/y): 18 to 26y	5,928	38,014	0.00 (-0.07, 0.06)	0.941
	change in SBP from childhood to early adulthood				0.691
Distance to closest blue space	mean difference in SBP at age 3y (mmHg)	5,946	38,132	0.34 (-0.10, 0.79)	0.129
	change in SBP in childhood (mmHg/y): 3 to 10y	5,946	38,132	-0.04 (-0.11, 0.04)	0.345
	change in SBP in adolescence (mmHg/y): 10 to 18y	5,946	38,132	-0.05 (-0.10, 0.01)	0.103
	change in SBP in adulthood (mmHg/y): 18 to 26y	5,946	38,132	0.05 (-0.02, 0.13)	0.160
	change in SBP from childhood to early adulthood				0.108
Size of blue space	mean difference in SBP at age 3y (mmHg)	5,944	38,118	0.06 (-0.20, 0.32)	0.668
	change in SBP in childhood (mmHg/y): 3 to 10y	5,944	38,118	-0.01 (-0.05, 0.04)	0.796
	change in SBP in adolescence (mmHg/y): 10 to 18y	5,944	38,118	0.00 (-0.03, 0.03)	0.971
	change in SBP in adulthood (mmHg/y): 18 to 26y	5,944	38,118	0.01 (-0.03, 0.05)	0.678
	change in SBP from childhood to early adulthood				0.968
Existence blue space within 300m buffer	mean difference in SBP at age 3y (mmHg)	5,946	38,132	0.69 (-0.77, 2.14)	0.355
	change in SBP in childhood (mmHg/y): 3 to 10y	5,946	38,132	-0.08 (-0.32, 0.16)	0.519
	change in SBP in adolescence (mmHg/y): 10 to 18y	5,946	38,132	0.14 (-0.04, 0.32)	0.120
	change in SBP in adulthood (mmHg/y): 18 to 26y	5,946	38,132	-0.11 (-0.33, 0.11)	0.325
	change in SBP from childhood to early adulthood				0.454
Distance to closest green space	mean difference in SBP at age 3y (mmHg)	5,946	38,132	-0.52 (-1.00, -0.03)	0.037
	change in SBP in childhood (mmHg/y): 3 to 10y	5,946	38,132	0.06 (-0.02, 0.15)	0.116
	change in SBP in adolescence (mmHg/y): 10 to 18y	5,946	38,132	-0.02 (-0.08, 0.03)	0.420
	change in SBP in adulthood (mmHg/y): 18 to 26y	5,946	38,132	0.04 (-0.04, 0.12)	0.297
	change in SBP from childhood to early adulthood				0.342
Size of green space	mean difference in SBP at age 3y (mmHg)	5,946	38,132	-0.06 (-0.53, 0.42)	0.822

	change in SBP in childhood (mmHg/y): 3 to 10y	5,946	38,132	0.03 (-0.04, 0.11)	0.396
	change in SBP in adolescence (mmHg/y): 10 to 18y	5,946	38,132	-0.02 (-0.08, 0.03)	0.430
	change in SBP in adulthood (mmHg/y): 18 to 26y	5,946	38,132	0.03 (-0.05, 0.11)	0.439
	change in SBP from childhood to early adulthood				0.727
Existence green space within 300m buffer	mean difference in SBP at age 3y (mmHg)	5,946	38,132	0.73 (-0.08, 1.54)	0.079
	change in SBP in childhood (mmHg/y): 3 to 10y	5,946	38,132	-0.10 (-0.23, 0.03)	0.144
	change in SBP in adolescence (mmHg/y): 10 to 18y	5,946	38,132	0.01 (-0.09, 0.11)	0.791
	change in SBP in adulthood (mmHg/y): 18 to 26y	5,946	38,132	-0.03 (-0.16, 0.10)	0.634
	change in SBP from childhood to early adulthood				0.478
Average greenness within 100m buffer	mean difference in SBP at age 3y (mmHg)	6,129	39,356	3.28 (-0.40, 6.95)	0.081
	change in SBP in childhood (mmHg/y): 3 to 10y	6,129	39,356	-0.25 (-0.85, 0.35)	0.418
	change in SBP in adolescence (mmHg/y): 10 to 18y	6,129	39,356	0.31 (-0.13, 0.74)	0.172
	change in SBP in adulthood (mmHg/y): 18 to 26y	6,129	39,356	0.15 (-0.43, 0.73)	0.609
	change in SBP from childhood to early adulthood				0.381
Average greenness within 300m buffer	mean difference in SBP at age 3y (mmHg)	6,129	39,356	2.17 (-1.70, 6.04)	0.272
	change in SBP in childhood (mmHg/y): 3 to 10y	6,129	39,356	0.01 (-0.62, 0.64)	0.976
	change in SBP in adolescence (mmHg/y): 10 to 18y	6,129	39,356	0.05 (-0.41, 0.50)	0.834
	change in SBP in adulthood (mmHg/y): 18 to 26y	6,129	39,356	0.03 (-0.57, 0.63)	0.928
	change in SBP from childhood to early adulthood				0.993
Average greenness within 500m buffer	mean difference in SBP at age 3y (mmHg)	6,129	39,356	3.24 (-0.71, 7.19)	0.108
	change in SBP in childhood (mmHg/y): 3 to 10y	6,129	39,356	-0.16 (-0.80, 0.49)	0.634
	change in SBP in adolescence (mmHg/y): 10 to 18y	6,129	39,356	-0.04 (-0.51, 0.42)	0.858
	change in SBP in adulthood (mmHg/y): 18 to 26y	6,129	39,356	0.00 (-0.61, 0.61)	0.998
	change in SBP from childhood to early adulthood				0.943
Food facilities density within 300m buffer	mean difference in SBP at age 3y (mmHg)	6,129	39,356	-0.02 (-0.26, 0.23)	0.899
	change in SBP in childhood (mmHg/y): 3 to 10y	6,129	39,356	-0.03 (-0.07, 0.01)	0.201
	change in SBP in adolescence (mmHg/y): 10 to 18y	6,129	39,356	0.01 (-0.02, 0.04)	0.400
	change in SBP in adulthood (mmHg/y): 18 to 26y	6,129	39,356	0.01 (-0.03, 0.04)	0.740
	change in SBP from childhood to early adulthood				0.541
NO_2	mean difference in SBP at age 3y (mmHg)	6,095	39,137	-0.23 (-0.68, 0.22)	0.311
	change in SBP in childhood (mmHg/y): 3 to 10y	6,095	39,137	0.01 (-0.06, 0.09)	0.717

	change in SBP in adolescence (mmHg/y): 10 to 18y	6,095	39,137	0.00 (-0.05, 0.05)	0.954
	change in SBP in adulthood (mmHg/y): 18 to 26y	6,095	39,137	-0.02 (-0.09, 0.05)	0.525
	change in SBP from childhood to early adulthood				0.897
$PM_{2.5}$	mean difference in SBP at age 3y (mmHg)	6,095	39,137	-0.54 (-0.99, -0.10)	0.017
	change in SBP in childhood (mmHg/y): 3 to 10y	6,095	39,137	0.10 (0.02, 0.17)	0.009
	change in SBP in adolescence (mmHg/y): 10 to 18y	6,095	39,137	-0.02 (-0.07, 0.04)	0.529
	change in SBP in adulthood (mmHg/y): 18 to 26y	6,095	39,137	-0.03 (-0.10, 0.04)	0.364
	change in SBP from childhood to early adulthood				0.046
PM_{10}	mean difference in SBP at age 3y (mmHg)	5,777	37,099	-0.54 (-0.97, -0.10)	0.015
	change in SBP in childhood (mmHg/y): 3 to 10y	5,777	37,099	0.05 (-0.02, 0.12)	0.194
	change in SBP in adolescence (mmHg/y): 10 to 18y	5,777	37,099	0.05 (-0.01, 0.10)	0.091
	change in SBP in adulthood (mmHg/y): 18 to 26y	5,777	37,099	0.00 (-0.08, 0.07)	0.902
	change in SBP from childhood to early adulthood				0.052

Adjusted for maternal education, age at delivery, ethnicity, area deprivation, and sex. Betas correspond to mean change in blood pressure by an interquartile range (IQR) increase in the exposure (for continuous exposures), or by change in category of exposure (for categorical exposures); the IQR and categories for each exposure are presented in Supplemental Table 4.

Supplemental Table 10. Associations between urban environmental exposures and changes in diastolic blood pressure (DBP) in ALSPAC, restricted to those with 3 or more blood pressure measurements

Exposure	Parameter	N	N obs	beta (95% CI)	p-value
Lden	mean difference in DBP at age 3y (mmHg)	4,199	26,875	-0.15 (-0.57, 0.27)	0.487
	change in DBP in childhood (mmHg/y): 3 to 10y	4,199	26,875	0.05 (-0.02, 0.12)	0.185
	change in DBP in adolescence (mmHg/y): 10 to 18y	4,199	26,875	-0.03 (-0.07, 0.02)	0.217
	change in DBP in adulthood (mmHg/y): 18 to 26y	4,199	26,875	0.01 (-0.06, 0.08)	0.759
	change in DBP from childhood to early adulthood				0.483
Lnight	mean difference in DBP at age 3y (mmHg)	4,199	26,875	-0.33 (-0.92, 0.26)	0.272
	change in DBP in childhood (mmHg/y): 3 to 10y	4,199	26,875	0.08 (-0.02, 0.18)	0.102
	change in DBP in adolescence (mmHg/y): 10 to 18y	4,199	26,875	-0.04 (-0.11, 0.02)	0.168
	change in DBP in adulthood (mmHg/y): 18 to 26y	4,199	26,875	0.04 (-0.06, 0.14)	0.390
	change in DBP from childhood to early adulthood				0.313
Inverse distance to nearest road	mean difference in DBP at age 3y (mmHg)	6,129	39,358	-0.15 (-0.44, 0.14)	0.307
	change in DBP in childhood (mmHg/y): 3 to 10y	6,129	39,358	0.02 (-0.03, 0.07)	0.382
	change in DBP in adolescence (mmHg/y): 10 to 18y	6,129	39,358	0.00 (-0.03, 0.04)	0.768
	change in DBP in adulthood (mmHg/y): 18 to 26y	6,129	39,358	-0.04 (-0.09, 0.01)	0.102
	change in DBP from childhood to early adulthood				0.296
Humidity	mean difference in DBP at age 3y (mmHg)	6,087	39,083	-1.54 (-2.00, -1.08)	4.64E-11
	change in DBP in childhood (mmHg/y): 3 to 10y	6,087	39,083	0.33 (0.26, 0.41)	3.36E-18
	change in DBP in adolescence (mmHg/y): 10 to 18y	6,087	39,083	-0.16 (-0.20, -0.12)	8.29E-13
	change in DBP in adulthood (mmHg/y): 18 to 26y	6,087	39,083	0.06 (-0.01, 0.13)	0.076
	change in DBP from childhood to early adulthood				1.59E-20
Mean temperature	mean difference in DBP at age 3y (mmHg)	6,096	39,143	0.20 (-0.29, 0.70)	0.420
	change in DBP in childhood (mmHg/y): 3 to 10y	6,096	39,143	-0.10 (-0.18, -0.02)	0.020
	change in DBP in adolescence (mmHg/y): 10 to 18y	6,096	39,143	0.08 (0.03, 0.13)	0.001
	change in DBP in adulthood (mmHg/y): 18 to 26y	6,096	39,143	0.03 (-0.05, 0.11)	0.508
	change in DBP from childhood to early adulthood				9.30E-04
Building density in 100m buffer	mean difference in DBP at age 3y (mmHg)	6,123	39,318	0.16 (-0.18, 0.50)	0.353
	change in DBP in childhood (mmHg/y): 3 to 10y	6,123	39,318	-0.03 (-0.08, 0.03)	0.316

	change in DBP in adolescence (mmHg/y): 10 to 18y	6,123	39,318	0.01 (-0.03, 0.04)	0.774
	change in DBP in adulthood (mmHg/y): 18 to 26y	6,123	39,318	-0.03 (-0.08, 0.03)	0.363
	change in DBP from childhood to early adulthood				0.583
Building density in 300m buffer	mean difference in DBP at age 3y (mmHg)	6,126	39,337	0.12 (-0.22, 0.47)	0.477
	change in DBP in childhood (mmHg/y): 3 to 10y	6,126	39,337	-0.03 (-0.08, 0.03)	0.366
	change in DBP in adolescence (mmHg/y): 10 to 18y	6,126	39,337	0.01 (-0.02, 0.05)	0.509
	change in DBP in adulthood (mmHg/y): 18 to 26y	6,126	39,337	-0.03 (-0.08, 0.03)	0.351
	change in DBP from childhood to early adulthood				0.666
Connectivity in 100m buffer	mean difference in DBP at age 3y (mmHg)	5,801	37,157	-0.18 (-0.47, 0.12)	0.236
	change in DBP in childhood (mmHg/y): 3 to 10y	5,801	37,157	0.01 (-0.04, 0.06)	0.658
	change in DBP in adolescence (mmHg/y): 10 to 18y	5,801	37,157	-0.02 (-0.05, 0.01)	0.246
	change in DBP in adulthood (mmHg/y): 18 to 26y	5,801	37,157	-0.02 (-0.07, 0.03)	0.418
	change in DBP from childhood to early adulthood				0.326
Connectivity in 300m buffer	mean difference in DBP at age 3y (mmHg)	6,108	39,217	0.05 (-0.28, 0.39)	0.748
	change in DBP in childhood (mmHg/y): 3 to 10y	6,108	39,217	-0.01 (-0.06, 0.04)	0.708
	change in DBP in adolescence (mmHg/y): 10 to 18y	6,108	39,217	0.00 (-0.03, 0.03)	0.986
	change in DBP in adulthood (mmHg/y): 18 to 26y	6,108	39,217	-0.02 (-0.07, 0.03)	0.484
	change in DBP from childhood to early adulthood				0.847
Facility density within 300m buffer	mean difference in DBP at age 3y (mmHg)	6,129	39,358	0.12 (-0.08, 0.32)	0.230
	change in DBP in childhood (mmHg/y): 3 to 10y	6,129	39,358	-0.03 (-0.06, 0.00)	0.086
	change in DBP in adolescence (mmHg/y): 10 to 18y	6,129	39,358	0.01 (-0.01, 0.03)	0.358
	change in DBP in adulthood (mmHg/y): 18 to 26y	6,129	39,358	-0.02 (-0.05, 0.01)	0.267
	change in DBP from childhood to early adulthood				0.268
Land use within 300m buffer	mean difference in DBP at age 3y (mmHg)	5,949	38,157	0.02 (-0.36, 0.40)	0.927
	change in DBP in childhood (mmHg/y): 3 to 10y	5,949	38,157	0.00 (-0.06, 0.06)	0.972
	change in DBP in adolescence (mmHg/y): 10 to 18y	5,949	38,157	0.02 (-0.02, 0.06)	0.390
	change in DBP in adulthood (mmHg/y): 18 to 26y	5,949	38,157	-0.02 (-0.08, 0.04)	0.537
	change in DBP from childhood to early adulthood				0.829
Agricultural area within 300m buffer	mean difference in DBP at age 3y (mmHg)	5,949	38,157	0.00 (-0.09, 0.08)	0.932
	change in DBP in childhood (mmHg/y): 3 to 10y	5,949	38,157	-0.01 (-0.02, 0.01)	0.473
	change in DBP in adolescence (mmHg/y): 10 to 18y	5,949	38,157	0.00 (-0.01, 0.01)	0.577

	change in DBP in adulthood (mmHg/y): 18 to 26y	5,949	38,157	0.01 (-0.01, 0.02)	0.332
	change in DBP from childhood to early adulthood				0.614
Airport within 300m buffer	mean difference in DBP at age 3y (mmHg)	5,949	38,157	-0.73 (-4.31, 2.85)	0.690
	change in DBP in childhood (mmHg/y): 3 to 10y	5,949	38,157	0.33 (-0.26, 0.91)	0.278
	change in DBP in adolescence (mmHg/y): 10 to 18y	5,949	38,157	-0.05 (-0.42, 0.32)	0.782
	change in DBP in adulthood (mmHg/y): 18 to 26y	5,949	38,157	0.45 (-0.07, 0.96)	0.089
	change in DBP from childhood to early adulthood				0.187
Continuous Urban Fabric within 300m buffer	mean difference in DBP at age 3y (mmHg)	5,949	38,157	0.01 (-0.30, 0.31)	0.962
	change in DBP in childhood (mmHg/y): 3 to 10y	5,949	38,157	-0.01 (-0.06, 0.04)	0.612
	change in DBP in adolescence (mmHg/y): 10 to 18y	5,949	38,157	0.02 (-0.01, 0.05)	0.193
	change in DBP in adulthood (mmHg/y): 18 to 26y	5,949	38,157	-0.06 (-0.11, 0.00)	0.038
	change in DBP from childhood to early adulthood				0.207
Industrial, commercial, public, military and private units within 300m buffer	mean difference in DBP at age 3y (mmHg)	5,949	38,157	-0.19 (-0.48, 0.10)	0.192
	change in DBP in childhood (mmHg/y): 3 to 10y	5,949	38,157	0.04 (-0.01, 0.09)	0.101
	change in DBP in adolescence (mmHg/y): 10 to 18y	5,949	38,157	-0.02 (-0.06, 0.01)	0.114
	change in DBP in adulthood (mmHg/y): 18 to 26y	5,949	38,157	0.01 (-0.04, 0.06)	0.615
	change in DBP from childhood to early adulthood				0.279
Discontinuous Low Density Urban Fabric within 300m buffer	mean difference in DBP at age 3y (mmHg)	5,949	38,157	0.10 (-0.29, 0.49)	0.605
	change in DBP in childhood (mmHg/y): 3 to 10y	5,949	38,157	-0.01 (-0.08, 0.05)	0.701
	change in DBP in adolescence (mmHg/y): 10 to 18y	5,949	38,157	0.02 (-0.02, 0.06)	0.438
	change in DBP in adulthood (mmHg/y): 18 to 26y	5,949	38,157	0.00 (-0.07, 0.06)	0.934
	change in DBP from childhood to early adulthood				0.875
Forests within 300m buffer	mean difference in DBP at age 3y (mmHg)	5,949	38,157	0.09 (-0.36, 0.55)	0.685
	change in DBP in childhood (mmHg/y): 3 to 10y	5,949	38,157	-0.03 (-0.10, 0.05)	0.492
	change in DBP in adolescence (mmHg/y): 10 to 18y	5,949	38,157	0.00 (-0.05, 0.05)	0.897
	change in DBP in adulthood (mmHg/y): 18 to 26y	5,949	38,157	0.01 (-0.07, 0.09)	0.804
	change in DBP from childhood to early adulthood				0.904
Mineral extraction and dump sites, constructions within 300m buffer	mean difference in DBP at age 3y (mmHg)	5,949	38,157	-0.19 (-0.69, 0.31)	0.460
	change in DBP in childhood (mmHg/y): 3 to 10y	5,949	38,157	0.06 (-0.03, 0.14)	0.188
	change in DBP in adolescence (mmHg/y): 10 to 18y	5,949	38,157	-0.07 (-0.12, -0.01)	0.013
	change in DBP in adulthood (mmHg/y): 18 to 26y	5,949	38,157	0.06 (-0.02, 0.15)	0.154

	change in DBP from childhood to early adulthood				0.087
Port areas within 300m buffer	mean difference in DBP at age 3y (mmHg)	5,949	38,157	2.17 (-4.06, 8.40)	0.495
	change in DBP in childhood (mmHg/y): 3 to 10y	5,949	38,157	-0.15 (-1.16, 0.87)	0.775
	change in DBP in adolescence (mmHg/y): 10 to 18y	5,949	38,157	-0.47 (-1.04, 0.11)	0.114
	change in DBP in adulthood (mmHg/y): 18 to 26y	5,949	38,157	0.01 (-1.16, 1.17)	0.993
	change in DBP from childhood to early adulthood				0.262
Transport networks and other constructed hard-surfaced areas within 300m buffer	mean difference in DBP at age 3y (mmHg)	5,949	38,157	0.06 (-0.27, 0.40)	0.717
	change in DBP in childhood (mmHg/y): 3 to 10y	5,949	38,157	-0.01 (-0.06, 0.05)	0.778
	change in DBP in adolescence (mmHg/y): 10 to 18y	5,949	38,157	0.00 (-0.03, 0.04)	0.894
	change in DBP in adulthood (mmHg/y): 18 to 26y	5,949	38,157	-0.02 (-0.07, 0.04)	0.580
	change in DBP from childhood to early adulthood				0.938
Green urban areas within 300m buffer	mean difference in DBP at age 3y (mmHg)	5,949	38,157	-0.03 (-0.36, 0.30)	0.848
	change in DBP in childhood (mmHg/y): 3 to 10y	5,949	38,157	0.03 (-0.02, 0.08)	0.280
	change in DBP in adolescence (mmHg/y): 10 to 18y	5,949	38,157	0.02 (-0.01, 0.06)	0.246
	change in DBP in adulthood (mmHg/y): 18 to 26y	5,949	38,157	-0.04 (-0.09, 0.02)	0.217
	change in DBP from childhood to early adulthood				0.226
Discontinuous Very Low Density Urban Fabric within 300m buffer	mean difference in DBP at age 3y (mmHg)	5,949	38,157	-0.64 (-1.87, 0.59)	0.309
	change in DBP in childhood (mmHg/y): 3 to 10y	5,949	38,157	0.06 (-0.14, 0.26)	0.549
	change in DBP in adolescence (mmHg/y): 10 to 18y	5,949	38,157	0.04 (-0.08, 0.16)	0.518
	change in DBP in adulthood (mmHg/y): 18 to 26y	5,949	38,157	-0.17 (-0.36, 0.01)	0.067
	change in DBP from childhood to early adulthood				0.268
Population density	mean difference in DBP at age 3y (mmHg)	6,002	38,517	0.29 (-0.08, 0.65)	0.120
	change in DBP in childhood (mmHg/y): 3 to 10y	6,002	38,517	-0.07 (-0.13, -0.01)	0.023
	change in DBP in adolescence (mmHg/y): 10 to 18y	6,002	38,517	0.01 (-0.03, 0.05)	0.608
	change in DBP in adulthood (mmHg/y): 18 to 26y	6,002	38,517	0.02 (-0.03, 0.08)	0.408
	change in DBP from childhood to early adulthood				0.103
Bus lines in 300m buffer	mean difference in DBP at age 3y (mmHg)	5,590	35,802	0.03 (-0.34, 0.39)	0.893
	change in DBP in childhood (mmHg/y): 3 to 10y	5,590	35,802	0.02 (-0.04, 0.08)	0.461
	change in DBP in adolescence (mmHg/y): 10 to 18y	5,590	35,802	-0.03 (-0.07, 0.01)	0.093
	change in DBP in adulthood (mmHg/y): 18 to 26y	5,590	35,802	0.03 (-0.03, 0.09)	0.353
	change in DBP from childhood to early adulthood				0.406

Bus lines in 500m buffer	mean difference in DBP at age 3y (mmHg)	5,936	38,095	0.09 (-0.29, 0.47)	0.639
	change in DBP in childhood (mmHg/y): 3 to 10y	5,936	38,095	-0.01 (-0.07, 0.06)	0.850
	change in DBP in adolescence (mmHg/y): 10 to 18y	5,936	38,095	-0.03 (-0.07, 0.01)	0.172
	change in DBP in adulthood (mmHg/y): 18 to 26y	5,936	38,095	0.02 (-0.04, 0.09)	0.462
	change in DBP from childhood to early adulthood				0.495
Walkability index	mean difference in DBP at age 3y (mmHg)	5,928	38,016	0.12 (-0.21, 0.45)	0.479
	change in DBP in childhood (mmHg/y): 3 to 10y	5,928	38,016	-0.03 (-0.08, 0.03)	0.313
	change in DBP in adolescence (mmHg/y): 10 to 18y	5,928	38,016	0.01 (-0.03, 0.04)	0.714
	change in DBP in adulthood (mmHg/y): 18 to 26y	5,928	38,016	-0.02 (-0.08, 0.03)	0.398
	change in DBP from childhood to early adulthood				0.627
Distance to closest blue space	mean difference in DBP at age 3y (mmHg)	5,946	38,134	0.15 (-0.20, 0.50)	0.395
	change in DBP in childhood (mmHg/y): 3 to 10y	5,946	38,134	-0.02 (-0.08, 0.03)	0.405
	change in DBP in adolescence (mmHg/y): 10 to 18y	5,946	38,134	-0.02 (-0.05, 0.02)	0.400
	change in DBP in adulthood (mmHg/y): 18 to 26y	5,946	38,134	0.05 (-0.01, 0.11)	0.106
	change in DBP from childhood to early adulthood				0.279
Size of blue space	mean difference in DBP at age 3y (mmHg)	5,944	38,120	-0.04 (-0.24, 0.16)	0.715
	change in DBP in childhood (mmHg/y): 3 to 10y	5,944	38,120	0.01 (-0.02, 0.04)	0.576
	change in DBP in adolescence (mmHg/y): 10 to 18y	5,944	38,120	0.00 (-0.02, 0.02)	0.875
	change in DBP in adulthood (mmHg/y): 18 to 26y	5,944	38,120	-0.01 (-0.04, 0.02)	0.565
	change in DBP from childhood to early adulthood				0.859
Existence blue space within 300m buffer	mean difference in DBP at age 3y (mmHg)	5,946	38,134	0.29 (-0.85, 1.43)	0.619
	change in DBP in childhood (mmHg/y): 3 to 10y	5,946	38,134	-0.05 (-0.23, 0.14)	0.631
	change in DBP in adolescence (mmHg/y): 10 to 18y	5,946	38,134	0.01 (-0.10, 0.13)	0.810
	change in DBP in adulthood (mmHg/y): 18 to 26y	5,946	38,134	-0.10 (-0.28, 0.08)	0.258
	change in DBP from childhood to early adulthood				0.649
Distance to closest green space	mean difference in DBP at age 3y (mmHg)	5,946	38,134	0.08 (-0.30, 0.46)	0.686
	change in DBP in childhood (mmHg/y): 3 to 10y	5,946	38,134	0.00 (-0.07, 0.06)	0.888
	change in DBP in adolescence (mmHg/y): 10 to 18y	5,946	38,134	-0.02 (-0.06, 0.02)	0.354
	change in DBP in adulthood (mmHg/y): 18 to 26y	5,946	38,134	0.00 (-0.06, 0.07)	0.891
	change in DBP from childhood to early adulthood				0.744
Size of green space	mean difference in DBP at age 3y (mmHg)	5,946	38,134	0.08 (-0.29, 0.46)	0.661

	change in DBP in childhood (mmHg/y): 3 to 10y	5,946	38,134	0.02 (-0.04, 0.08)	0.531
	change in DBP in adolescence (mmHg/y): 10 to 18y	5,946	38,134	-0.03 (-0.07, 0.01)	0.174
	change in DBP in adulthood (mmHg/y): 18 to 26y	5,946	38,134	0.02 (-0.04, 0.08)	0.522
	change in DBP from childhood to early adulthood				0.598
Existence green space within 300m buffer	mean difference in DBP at age 3y (mmHg)	5,946	38,134	-0.06 (-0.69, 0.57)	0.850
	change in DBP in childhood (mmHg/y): 3 to 10y	5,946	38,134	0.00 (-0.10, 0.11)	0.947
	change in DBP in adolescence (mmHg/y): 10 to 18y	5,946	38,134	0.03 (-0.03, 0.10)	0.312
	change in DBP in adulthood (mmHg/y): 18 to 26y	5,946	38,134	0.02 (-0.09, 0.12)	0.754
	change in DBP from childhood to early adulthood				0.571
Average greenness within 100m buffer	mean difference in DBP at age 3y (mmHg)	6,129	39,358	0.71 (-2.15, 3.57)	0.626
	change in DBP in childhood (mmHg/y): 3 to 10y	6,129	39,358	-0.25 (-0.72, 0.21)	0.286
	change in DBP in adolescence (mmHg/y): 10 to 18y	6,129	39,358	0.21 (-0.08, 0.51)	0.157
	change in DBP in adulthood (mmHg/y): 18 to 26y	6,129	39,358	0.06 (-0.41, 0.52)	0.808
	change in DBP from childhood to early adulthood				0.363
Average greenness within 300m buffer	mean difference in DBP at age 3y (mmHg)	6,129	39,358	0.31 (-2.71, 3.32)	0.842
	change in DBP in childhood (mmHg/y): 3 to 10y	6,129	39,358	-0.10 (-0.59, 0.39)	0.678
	change in DBP in adolescence (mmHg/y): 10 to 18y	6,129	39,358	0.16 (-0.14, 0.47)	0.295
	change in DBP in adulthood (mmHg/y): 18 to 26y	6,129	39,358	0.08 (-0.41, 0.56)	0.762
	change in DBP from childhood to early adulthood				0.617
Average greenness within 500m buffer	mean difference in DBP at age 3y (mmHg)	6,129	39,358	-0.83 (-3.90, 2.24)	0.597
	change in DBP in childhood (mmHg/y): 3 to 10y	6,129	39,358	0.12 (-0.38, 0.61)	0.647
	change in DBP in adolescence (mmHg/y): 10 to 18y	6,129	39,358	-0.02 (-0.33, 0.29)	0.904
	change in DBP in adulthood (mmHg/y): 18 to 26y	6,129	39,358	0.22 (-0.27, 0.72)	0.381
	change in DBP from childhood to early adulthood				0.770
Food facilities density within 300m buffer	mean difference in DBP at age 3y (mmHg)	6,129	39,358	0.18 (-0.01, 0.37)	0.063
	change in DBP in childhood (mmHg/y): 3 to 10y	6,129	39,358	-0.04 (-0.07, -0.01)	0.011
	change in DBP in adolescence (mmHg/y): 10 to 18y	6,129	39,358	0.01 (-0.01, 0.03)	0.434
	change in DBP in adulthood (mmHg/y): 18 to 26y	6,129	39,358	0.00 (-0.03, 0.03)	0.845
	change in DBP from childhood to early adulthood				0.093
NO_2	mean difference in DBP at age 3y (mmHg)	6,095	39,139	-0.10 (-0.45, 0.24)	0.558
	change in DBP in childhood (mmHg/y): 3 to 10y	6,095	39,139	0.03 (-0.03, 0.08)	0.374

	change in DBP in adolescence (mmHg/y): 10 to 18y	6,095	39,139	0.00 (-0.04, 0.03)	0.896
	change in DBP in adulthood (mmHg/y): 18 to 26y	6,095	39,139	-0.04 (-0.10, 0.01)	0.110
	change in DBP from childhood to early adulthood				0.268
$PM_{2.5}$	mean difference in DBP at age 3y (mmHg)	6,095	39,139	-0.38 (-0.73, -0.03)	0.034
	change in DBP in childhood (mmHg/y): 3 to 10y	6,095	39,139	0.10 (0.04, 0.16)	4.77E-04
	change in DBP in adolescence (mmHg/y): 10 to 18y	6,095	39,139	-0.06 (-0.10, -0.03)	7.03E-04
	change in DBP in adulthood (mmHg/y): 18 to 26y	6,095	39,139	0.01 (-0.05, 0.06)	0.797
	change in DBP from childhood to early adulthood				2.97E-04
PM_{10}	mean difference in DBP at age 3y (mmHg)	5,777	37,100	-0.31 (-0.64, 0.03)	0.075
	change in DBP in childhood (mmHg/y): 3 to 10y	5,777	37,100	0.04 (-0.02, 0.09)	0.162
	change in DBP in adolescence (mmHg/y): 10 to 18y	5,777	37,100	0.00 (-0.03, 0.04)	0.847
	change in DBP in adulthood (mmHg/y): 18 to 26y	5,777	37,100	-0.06 (-0.12, 0.00)	0.048
	change in DBP from childhood to early adulthood				0.095

Adjusted for maternal education, age at delivery, ethnicity, area deprivation, and sex. Betas correspond to mean change in blood pressure by an interquartile range (IQR) increase in the exposure (for continuous exposures), or by change in category of exposure (for categorical exposures); the IQR and categories for each exposure are presented in Supplemental Table 4.

Supplemental Table 11. Associations of urban environmental exposures with systolic (SBP) and diastolic blood pressure (DBP) in males and females in ALSPAC.

Exposure	Parameter	Males beta (95% CI)	p-value	Females beta (95% CI)	p-value	p-value for sex interaction
**Systolic blood pressure**						
Humidity	mean difference in SBP at age 3y (mmHg)	-2.06 (-2.83, -1.30)	1.19E-07	-1.95 (-2.77, -1.13)	3.24E-06	0.852
	change in SBP in childhood (mmHg/y)	0.32 (0.19, 0.45)	1.11E-06	0.30 (0.17, 0.44)	1.33E-05	0.872
	change in SBP in adolescence (mmHg/y)	0.02 (-0.06, 0.11)	0.572	0.01 (-0.07, 0.09)	0.847	0.755
	change in SBP in adulthood (mmHg/y)	-0.18 (-0.31, -0.04)	0.009	-0.08 (-0.18, 0.02)	0.127	0.219
	change from childhood to early adulthood		1.16E-07		2.20E-05	0.658
Mean temperature	mean difference in SBP at age 3y (mmHg)	1.00 (0.17, 1.82)	0.018	0.84 (-0.07, 1.74)	0.069	0.803
	change in SBP in childhood (mmHg/y)	-0.25 (-0.39, -0.11)	4.29E-04	-0.13 (-0.28, 0.02)	0.099	0.249
	change in SBP in adolescence (mmHg/y)	0.10 (0.00, 0.20)	0.042	-0.03 (-0.12, 0.07)	0.587	0.091
	change in SBP in adulthood (mmHg/y)	0.01 (-0.15, 0.16)	0.933	0.08 (-0.03, 0.20)	0.158	0.508
	change from childhood to early adulthood		0.004		0.137	0.366
**Diastolic blood pressure**						
Humidity	mean difference in DBP at age 3y (mmHg)	-1.58 (-2.18, -0.97)	3.80E-07	-1.58 (-2.23, -0.92)	2.35E-06	0.974
	change in DBP in childhood (mmHg/y)	0.33 (0.23, 0.43)	9.51E-11	0.35 (0.24, 0.45)	3.46E-10	0.892
	change in DBP in adolescence (mmHg/y)	-0.13 (-0.20, -0.07)	5.04E-05	-0.18 (-0.24, -0.13)	2.75E-10	0.256
	change in DBP in adulthood (mmHg/y)	0.03 (-0.09, 0.14)	0.664	0.07 (-0.02, 0.15)	0.114	0.535
	change from childhood to early adulthood		4.15E-10		1.27E-12	0.708
Mean temperature	mean difference in DBP at age 3y (mmHg)	0.16 (-0.49, 0.82)	0.628	0.17 (-0.55, 0.89)	0.648	0.897
	change in DBP in childhood (mmHg/y)	-0.12 (-0.22, -0.01)	0.037	-0.06 (-0.18, 0.06)	0.305	0.491
	change in DBP in adolescence (mmHg/y)	0.11 (0.04, 0.19)	0.004	0.05 (-0.02, 0.12)	0.147	0.384
	change in DBP in adulthood (mmHg/y)	0.03 (-0.10, 0.16)	0.702	0.05 (-0.05, 0.14)	0.343	0.879
	change from childhood to early adulthood		0.005		0.152	0.810
$PM_{2.5}$	mean difference in DBP at age 3y (mmHg)	-0.30 (-0.75, 0.16)	0.197	-0.56 (-1.07, -0.05)	0.031	0.566
	change in DBP in childhood (mmHg/y)	0.09 (0.02, 0.17)	0.015	0.14 (0.05, 0.22)	0.001	0.529
	change in DBP in adolescence (mmHg/y)	-0.06 (-0.11, 0.00)	0.041	-0.07 (-0.12, -0.03)	0.002	0.548

	change in DBP in adulthood (mmHg/y)	-0.03 (-0.12, 0.07)	0.588	0.02 (-0.05, 0.08)	0.638	0.475
	change from childhood to early adulthood		0.021		0.002	0.836

Adjusted for maternal education, age at delivery, ethnicity, and area deprivation. Betas correspond to mean change in blood pressure by an interquartile range (IQR) increase in the exposure (for continuous exposures), or by change in category of exposure (for categorical exposures); the IQR and categories for each exposure are presented in Supplemental Table 4.

Supplemental Table 12. Distribution of baseline characteristics, environmental exposures and outcomes in the participants included in the analysis in GenR, EDEN, PANIC and NFBC1986

Variable	GenR (N= 6,331)	EDEN Nancy (N= 628)	EDEN Poitiers (N= 713)	PANIC (N= 369)	NFBC1986 (N= 1,220)
	N (%), mean (SD) or median (IQR)				
Sex					
Males	3,157 (49.9)	309 (49.2)	397 (55.7)	186 (50.4)	475 (38.0)
Females	3,174 (50.1)	319 (50.8)	316 (44.3)	183 (49.6)	745 (61.1)
Maternal education					
Low	582 (9.2)	21 (3.3)	42 (5.9)	107 (29.0)	457 (37.5)
Medium	2,761 (43.6)	190 (30.3)	304 (42.6)	166 (45.0)	435 (35.6)
High	2,988 (47.2)	417 (66.4)	367 (51.5)	96 (26.0)	328 (26.9)
Ethnicity					NA
White	-	-	-	368 (99.7)	-
Non-White	-	-	-	1 (0.3)	-
Western	3,847 (60.8)	537 (99.6)	635 (99.4)	-	-
Non-Western	1,860 (29.4)	-	1 (0.2)	-	-
Mixed	624 (9.9)	2 (0.4)	3 (0.5)	-	-
Area socioeconomic status				NA	NA
1st quintile (less deprived)	671 (10.6)	142 (22.6)	230 (32.3)	-	-
2nd quintile	691 (10.9)	115 (18.3)	181 (25.4)	-	-
3rd quintile	743 (11.7)	94 (15.0)	120 (16.8)	-	-
4th quintile	694 (11.0)	128 (20.4)	116 (16.3)	-	-
5th quintile (more deprived)	3,532 (55.8)	149 (23.7)	66 (9.3)	-	-
Age of mother at birth (years), mean (SD)	31.0 (5.0)	30.2 (4.7)	29.8 (4.8)	30.0 (5.2)	28.5 (5.5)
Humidity (%), median (IQR)	81.2 (1.7)	77.4 (3.7)	76.9 (4.6)	84.5 (5.6)	77.5 (9.9)
Mean temperature (Celsius), median (IQR)	10.8 (2.1)	9.8 (3.2)	12.1 (3.3)	6.7 (5.6)	1.4 (11.1)
$PM_{2.5}$ (µg/m^3), median (IQR)	19.7 (3.9)	17.8 (2.5)	16.4 (2.3)	-	-
Age at 1st assessment (years), mean (SD)	2.1 (0.10)	3.1 (0.08)	3.1 (0.07)	7.6 (0.39)	16.0 (0.38)
Age at 2nd assessment (years), mean (SD)	6.1 (0.42)	5.6 (0.18)	5.6 (0.12)	9.8 (0.43)	34.1 (0.59)

Age at 3rd assessment (years), mean (SD)	9.8 (0.38)	-	-	15.8 (0.44)	-
SBP at 1st assessment (mmHg), mean (SD)	101.6 (11.4)	97.8 (7.3)	88.2 (6.3)	100.3 (7.5)	114.4 (12.4)
SBP at 2nd assessment (mmHg), mean (SD)	103.1 (8.0)	102.1 (7.4)	101.9 (8.6)	100.2 (7.8)	112.0 (12.6)
SBP at 3rd assessment (mmHg), mean (SD)	103.8 (7.7)	-	-	113.2 (10.0)	-
DBP at 1st assessment (mmHg), mean (SD)	62.6 (11.6)	53.8 (7.1)	45.7 (6.2)	61.5 (7.04)	67.3 (7.4)
DBP at 2nd assessment (mmHg), mean (SD)	61.3 (6.7)	59.2 (6.2)	49.8 (7.4)	61.4 (7.7)	74.5 (8.8)
DBP at 3rd assessment (mmHg), mean (SD)	59.4 (6.2)	-	-	67.4 (9.52)	-

DBP: diastolic blood pressure; IQR: interquartile range; NA: not applicable; $PM_{2.5}$: particulate matter <2.5 µm; SBP: systolic blood pressure; SD: standard deviation

Supplemental Table 13. Association between confounders and urban environmental exposures in each cohort

Confounder	Humidity		Temperature		$PM_{2.5}$	
	β (95% CI)	p-value	β (95% CI)	p-value	β (95% CI)	p-value
**ALSPAC**						
Maternal education (ref high)						
Medium	0.07 (-0.01, 0.16)	0.089	-0.03 (-0.11, 0.06)	0.536	0.11 (0.06, 0.17)	<0.001
Low	0.00 (-0.11, 0.11)	0.987	0.05 (-0.06, 0.16)	0.375	0.12 (0.05, 0.19)	0.001
Ethnicity (Non-White)	0.00 (-0.20, 0.21)	0.966	-0.13 (-0.34, 0.08)	0.230	0.29 (0.15, 0.42)	<0.001
Area socioeconomic status (ref 1st quintile)						
2nd quintile	-0.05 (-0.15, 0.04)	0.284	-0.01 (-0.11, 0.09)	0.868	-0.09 (-0.16, -0.02)	0.010
3rd quintile	-0.08 (-0.17, 0.01)	0.086	-0.01 (-0.11, 0.08)	0.774	0.08 (0.02, 0.14)	0.013
4th quintile	-0.04 (-0.13, 0.06)	0.426	-0.01 (-0.10, 0.09)	0.875	0.22 (0.16, 0.28)	<0.001
5th quintile (more deprived)	-0.13 (-0.23, -0.04)	0.006	0.04 (-0.05, 0.14)	0.381	0.30 (0.24, 0.36)	<0.001
Age of mother at birth (years)	0.00 (0.00, 0.01)	0.159	0.00 (-0.01, 0.01)	0.636	-0.01 (-0.02, -0.01)	<0.001
**GenR**						
Maternal education (ref high)						
Medium	-0.06 (-0.07, -0.04)	<0.001	0.01 (-0.00, 0.01)	0.078	0.16 (0.14, 0.17)	<0.001
Low	0.06 (0.04, 0.08)	<0.001	-0.01 (-0.03, -0.00)	0.022	-0.19 (-0.22, -0.17)	<0.001
Ethnicity (Western)						
Non-Western	0.12 (0.10, 0.14)	<0.001	-0.08 (-0.09, -0.08)	<0.001	-0.19 (-0.21, -0.17)	<0.001
Mixed	0.09 (0.07, 0.10)	<0.001	-0.08 (-0.09, -0.07)	<0.001	-0.21 (-0.24, -0.19)	<0.001
Area socioeconomic status (ref 1st quintile)						
2nd quintile	-0.21 (-0.24, -0.19)	<0.001	0.18 (0.16, 0.19)	<0.001	0.62 (0.59, 0.65)	<0.001
3rd quintile	-0.08 (-0.11, -0.06)	<0.001	0.14 (0.12, 0.15)	<0.001	-0.15 (-0.18, -0.12)	<0.001
4th quintile	-0.13 (-0.15 -0.11)	<0.001	0.09 (0.08, 0.11)	<0.001	0.61 (0.58, 0.64)	<0.001
5th quintile (more deprived)	-0.12 (-0.14, -0.11)	<0.001	0.12 (0.11, 0.13)	<0.001	0.35 (0.33, 0.38)	<0.001
Age of mother at birth (years)	0.01 (0.00, 0.01)	<0.001	0.00 (0.00, 0.00)	<0.001	-0.01 (-0.01, -0.01)	<0.001
**EDEN (Nancy)**						
Maternal education (ref high)						
Medium	0.26 (-0.03, 0.56)	0.082	-0.06 (-0.31, 0.20)	0.666	-0.15 (-0.39, 0.09)	0.222

Low	-0.43 (-1.21, 0.35)	0.277	0.14 (-0.53, 0.81)	0.677	0.42 (-0.21, 1.04)	0.189
Area socioeconomic status (ref 1st quintile)						
2nd quintile	0.23 (-0.19, 0.66)	0.276	-0.08 (-0.45, 0.28)	0.648	0.02 (-0.32, 0.35)	0.914
3rd quintile	0.42 (-0.01, 0.86)	0.058	-0.22 (-0.60, 0.15)	0.245	0.72 (0.37, 1.07)	<0.001
4th quintile	0.01 (-0.39, 0.42)	0.944	0.09 (-0.26, 0.44)	0.632	0.55 (0.23, 0.88)	0.001
5th quintile (more deprived)	0.38 (-0.01, 0.78)	0.057	-0.15 (-0.49, 0.19)	0.381	0.34 (0.03, 0.66)	0.034
Age of mother at birth (years)	0.06 (0.03, 0.08)	<0.001	-0.03 (-0.06, -0.01)	0.010	-0.02 (-0.05, 0.00)	0.038
**EDEN (Poitiers)**						
Maternal education (ref high)						
Medium	-0.05 (-0.43, 0.32)	0.783	-0.10 (-0.32, 0.12)	0.366	0.05 (-0.20, 0.31)	0.692
Low	-0.04 (-0.88, 0.79)	0.919	-0.39 (-0.87, 0.10)	0.118	0.74 (0.19, 1.30)	0.009
Area socioeconomic status (ref 1st quintile)						
2nd quintile	-0.32 (-0.80, 0.16)	0.186	0.37 (0.10, 0.65)	0.008	-0.10 (-0.42, 0.22)	0.543
3rd quintile	0.37 (-0.18, 0.91)	0.189	-0.15 (-0.46, 0.17)	0.363	0.25 (-0.11, 0.62)	0.173
4th quintile	0.32 (-0.25, 0.88)	0.273	-0.19 (-0.51, 0.14)	0.261	0.80 (0.42, 1.17)	<0.001
5th quintile (more deprived)	0.38 (-0.30, 1.07)	0.270	-0.07 (-0.46, 0.32)	0.727	0.34 (-0.12, 0.80)	0.142
Age of mother at birth (years)	0.04 (0.00, 0.08)	0.040	-0.02 (-0.04, 0.00)	0.058	0.01 (-0.02, 0.03)	0.648
**PANIC**						
Maternal education (ref high)						
Medium	0.99 (0.39, 1.59)	0.001	-0.26 (-0.60, 0.07)	0.124	-	-
Low	0.87 (0.19,1.55)	0.011	0.099 (-0.28, 0.48)	0.611	-	-
Ethnicity (Non-White)	-0.90 (-2.7, 0.90)	0.328	0.98 (-0.03, 2.00)	0.057	-	-
Age of mother at birth (years)	0.07 (0.02, 0.12)	0.003	0.00 (-0.02, 0.03)	0.846	-	-
**NFBC1986**						
Maternal education (ref high)						
Medium	1.14 (0.14, 2.14)	0.026	-0.98 (-2.11, 0.15)	0.089	-	-
Low	0.23 (-0.75, 1.22)	0.641	-0.98 (-2.10, 0.13)	0.084	-	-
Age of mother at birth (years)	-0.06 (-0.13, 0.01)	0.105	-0.02 (-0.06, 0.10)	0.610	-	-

Supplemental Table 14. Associations of humidity and temperature, mutually adjusted, with changes in systolic (SBP) and diastolic blood pressure (DBP) in ALSPAC

Exposure	Parameter	N	N obs	beta (95% CI)	p-value
**Systolic blood pressure**					
Humidity	mean difference in SBP at age 3y (mmHg)	7,402	40,925	-2.29 (-2.91, -1.68)	1.73E-13
	change in SBP in childhood (mmHg/y): 3 to 10y	7,402	40,925	0.30 (0.21, 0.40)	3.19E-10
	change in SBP in adolescence (mmHg/y): 10 to 18y	7,402	40,925	0.06 (0.00, 0.12)	0.062
	change in SBP in adulthood (mmHg/y): 18 to 26y	7,402	40,925	-0.11 (-0.20, -0.03)	0.010
	change in SBP from childhood to early adulthood				9.95E-14
Mean temperature	mean difference in SBP at age 3y (mmHg)	7,413	40,988	0.74 (0.10, 1.38)	0.024
	change in SBP in childhood (mmHg/y): 3 to 10y	7,413	40,988	-0.18 (-0.28, -0.07)	0.001
	change in SBP in adolescence (mmHg/y): 10 to 18y	7,413	40,988	0.02 (-0.06, 0.09)	0.630
	change in SBP in adulthood (mmHg/y): 18 to 26y	7,413	40,988	0.02 (-0.08, 0.12)	0.653
	change in SBP from childhood to early adulthood				0.007
**Diastolic blood pressure**					6.33E-12
Humidity	mean difference in DBP at age 3y (mmHg)	7,402	40,925	-1.68 (-2.15, -1.20)	1.01E-19
	change in DBP in childhood (mmHg/y): 3 to 10y	7,402	40,925	0.34 (0.27, 0.42)	4.41E-13
	change in DBP in adolescence (mmHg/y): 10 to 18y	7,402	40,925	-0.16 (-0.20, -0.12)	0.115
	change in DBP in adulthood (mmHg/y): 18 to 26y	7,402	40,925	0.06 (-0.01, 0.13)	6.16E-22
	change in DBP from childhood to early adulthood				0.466
Mean temperature	mean difference in DBP at age 3y (mmHg)	7,413	40,988	0.19 (-0.32, 0.69)	0.033
	change in DBP in childhood (mmHg/y): 3 to 10y	7,413	40,988	-0.09 (-0.17, -0.01)	0.002
	change in DBP in adolescence (mmHg/y): 10 to 18y	7,413	40,988	0.08 (0.03, 0.13)	0.342
	change in DBP in adulthood (mmHg/y): 18 to 26y	7,413	40,988	0.04 (-0.04, 0.12)	0.001
	change in DBP from childhood to early adulthood				1.73E-13

DBP: diastolic blood pressure; SBP: systolic blood pressure

Adjusted for maternal education, age at delivery, ethnicity, area deprivation, and month of birth. Betas correspond to mean change in blood pressure by an interquartile range (IQR) increase in the exposure (for continuous exposures), or by change in category of exposure (for categorical exposures); the IQR and categories for each exposure are presented in Supplemental Table 4.

Supplemental Table 15. Association between being a complete case and trajectory of systolic and diastolic blood pressure from childhood to early adulthood in ALSPAC (n=9,234)

	**Mean (95%CI)**
**Systolic blood pressure (SBP)**	
SBP at 3y, mmHg	94.6 (93.7, 95.5)
Mean difference in SBP at 3y in complete cases, mmHg	-1.73 (-2.69, -0.77)
Change in SBP in childhood (3 to 10y), mmHg/y	1.31 (1.16, 1.47)
Change in SBP in childhood in complete cases, mmHg/y	0.25 (0.08, 0.41)
Change in SBP in adolescence (10 to 18y), mmHg/y	2.55 (2.46, 2.65)
Change in SBP in adolescence in complete cases, mmHg/y	0.01 (-0.09, 0.12)
Change in SBP in early adulthood (18 to 24y), mmHg/y	-0.88 (-1.00, -0.76)
Change in SBP in adulthood in complete cases, mmHg/y	-0.14 (-0.28, -0.01)
**Diastolic blood pressure (DBP)**	
DBP at 3y, mmHg	56.5 (55.8, 57.2)
Change in DBP in childhood (3 to 10y), mmHg/y	0.10 (-0.01, 0.22)
Change in DBP in childhood in complete cases, mmHg/y	0.06 (-0.06, 0.19)
Change in DBP in adolescence (10 to 18y), mmHg/y	1.07 (1.00, 1.14)
Change in DBP in adolescence in complete cases, mmHg/y	-0.02, (-0.09, 0.05)
Change in DBP in early adulthood (18 to 24y), mmHg/y	0.15 (0.05, 0.25)
Change in DBP in adulthood in complete cases, mmHg/y	-0.01 (-0.12, 0.09)

Complete case corresponds to having complete data in all confounders used in the analyses i.e., maternal education, age at delivery, ethnicity, and area deprivation). Analysis was adjusted for sex.
DBP: diastolic blood pressure; SBP: systolic blood pressure

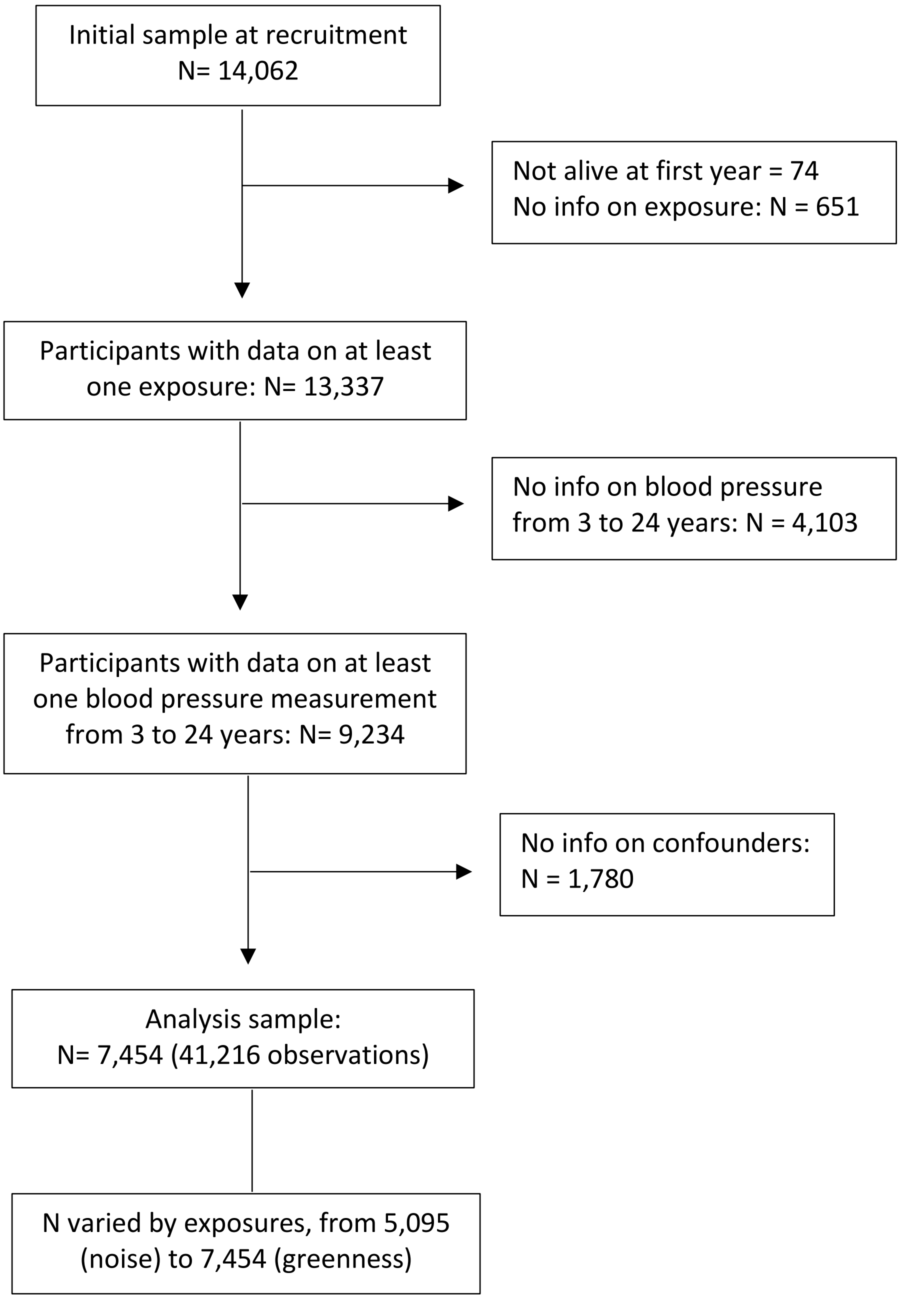

Supplemental Figure 1. Flow of participants in ALSPAC

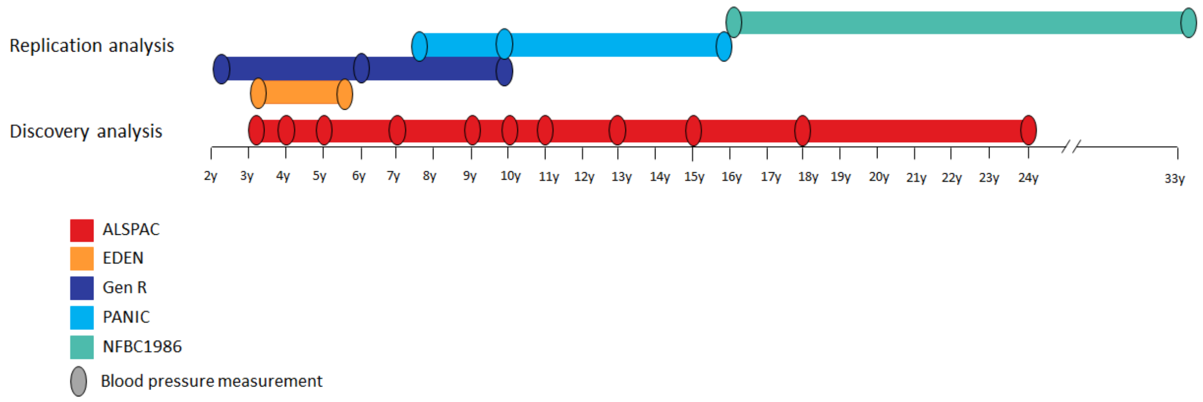

Supplemental Figure 2. Average age at the time of blood pressure measurements available in each study

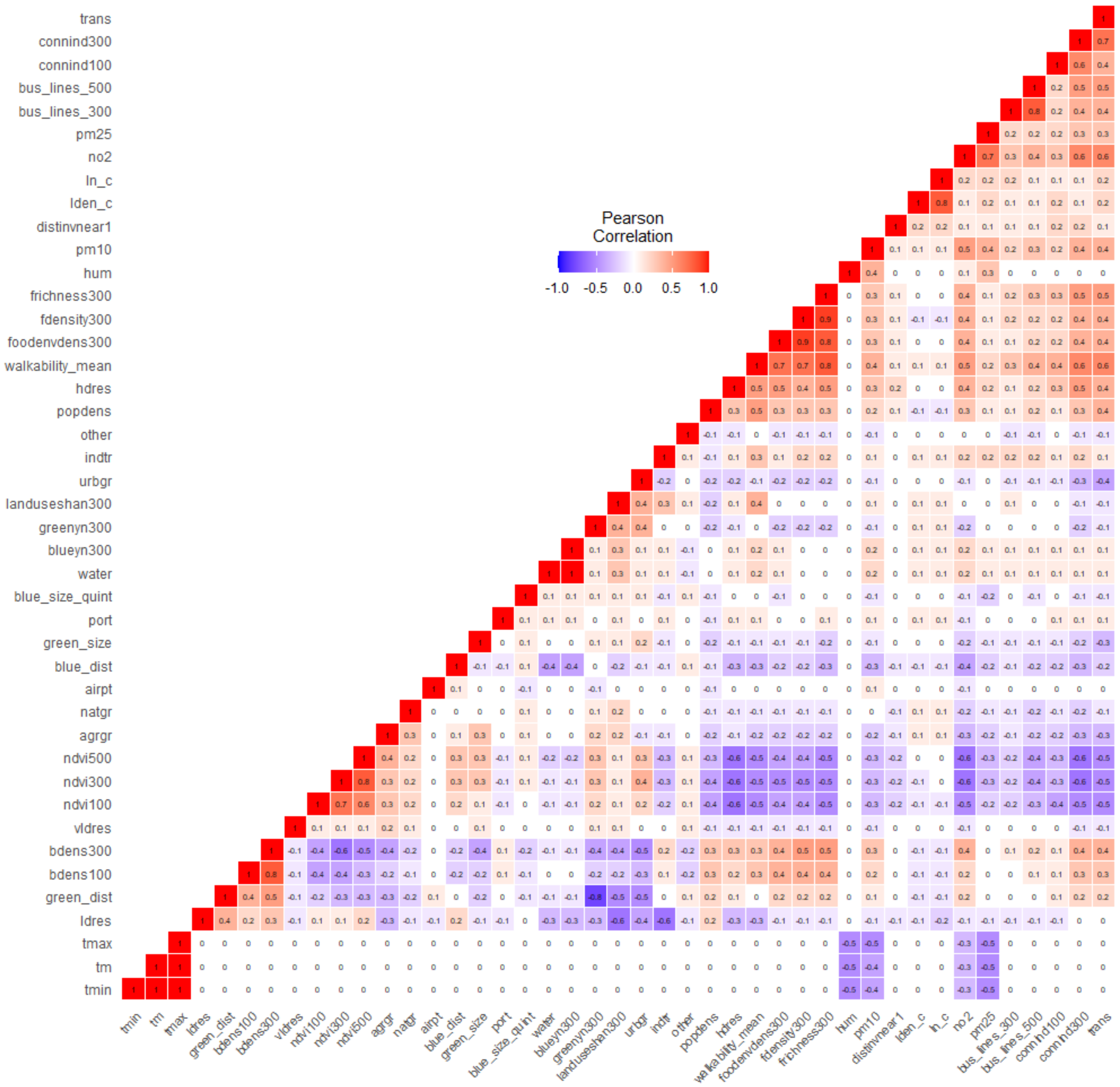

Supplemental Figure 3. Correlation matrix of the urban environmental exposures in ALSPAC.

Four exposures were excluded from the analyses due to very high correlation: minimum and maximum temperature (r=0.99 with mean temperature), % water land use within 300m buffer (r=0.99 with existence of blue area within 300m buffer), and facility richness within 300m buffer (r=0.91 with facility density within 300m buffer).

A

B

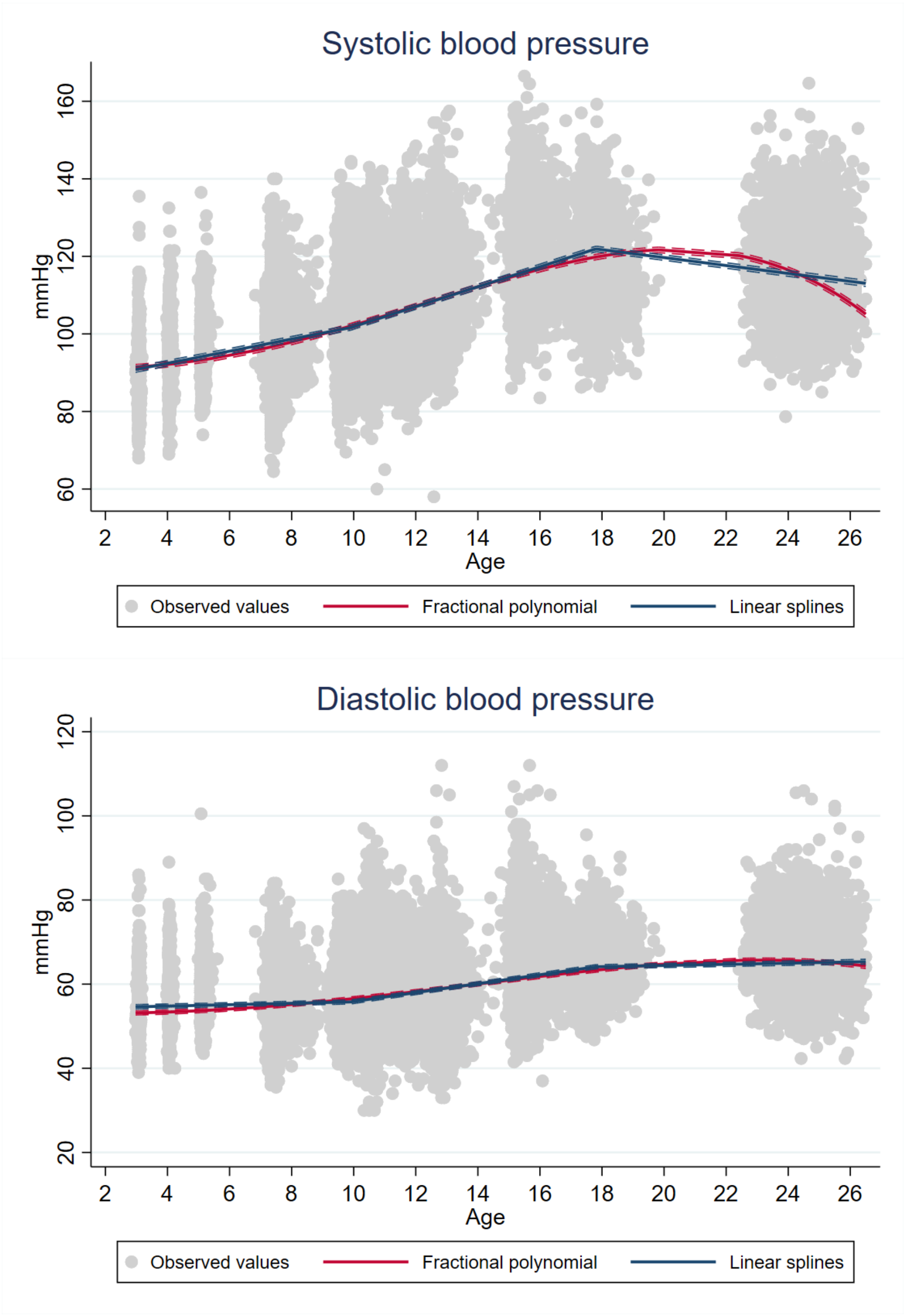

Supplemental Figure 4. Observed and average predicted (A) systolic blood pressure and (B) diastolic blood pressure trajectories in the ALSPAC.

Grey points are the observed blood pressure values, the red line is the average predicted trajectory using fractional polynomials and the blue line is the average predicted trajectory using linear splines. All covariates used in the adjustment were set to the mean value or the reference category: maternal education (high), age at delivery (28.9 years), ethnicity (White), area deprivation (least deprived), and sex (male).

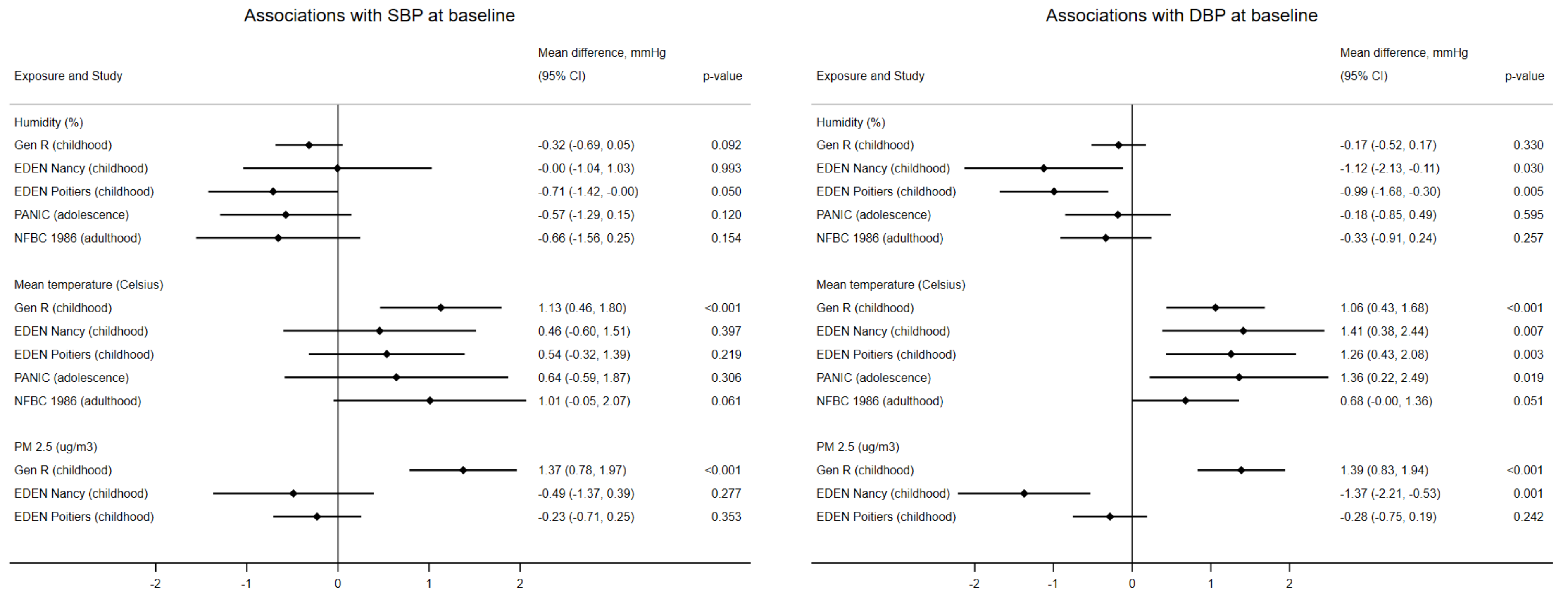

Supplemental Figure 5. Associations of humidity, mean temperature, and $PM_{2.5}$ with SBP and DBP at baseline in GenR, EDEN, PANIC and NFBC1986.

DBP: diastolic blood pressure; $PM_{2.5}$: particulate matter <2.5 µm; SBP: systolic blood pressure
